# Supplementary material for: Ablation of ERO1A induces lethal endoplasmic reticulum stress responses and immunogenic cell death to activate anti-tumor immunity
Source: Cell Rep Med. 2023 Sep 27;4(10):101206. doi: 10.1016/j.xcrm.2023.101206 (PMC10591028; doi:10.1016/j.xcrm.2023.101206)
Supplement: Document S1. Figures S1–S11 and Tables S1 [file mmc1.pdf]

Cell Reports Medicine, Volume *4*

Supplemental information

Ablation of ERO1A induces lethal endoplasmic reticulum stress responses and immunogenic cell death to activate anti-tumor immunity

Lihui Liu, Sini Li, Yan Qu, Hua Bai, Xiangyu Pan, Jian Wang, Zhijie Wang, Jianchun Duan, Jia Zhong, Rui Wan, Kailun Fei, Jiachen Xu, Li Yuan, Chao Wang, Pei Xue, Xue Zhang, Zixiao Ma, and Jie Wang

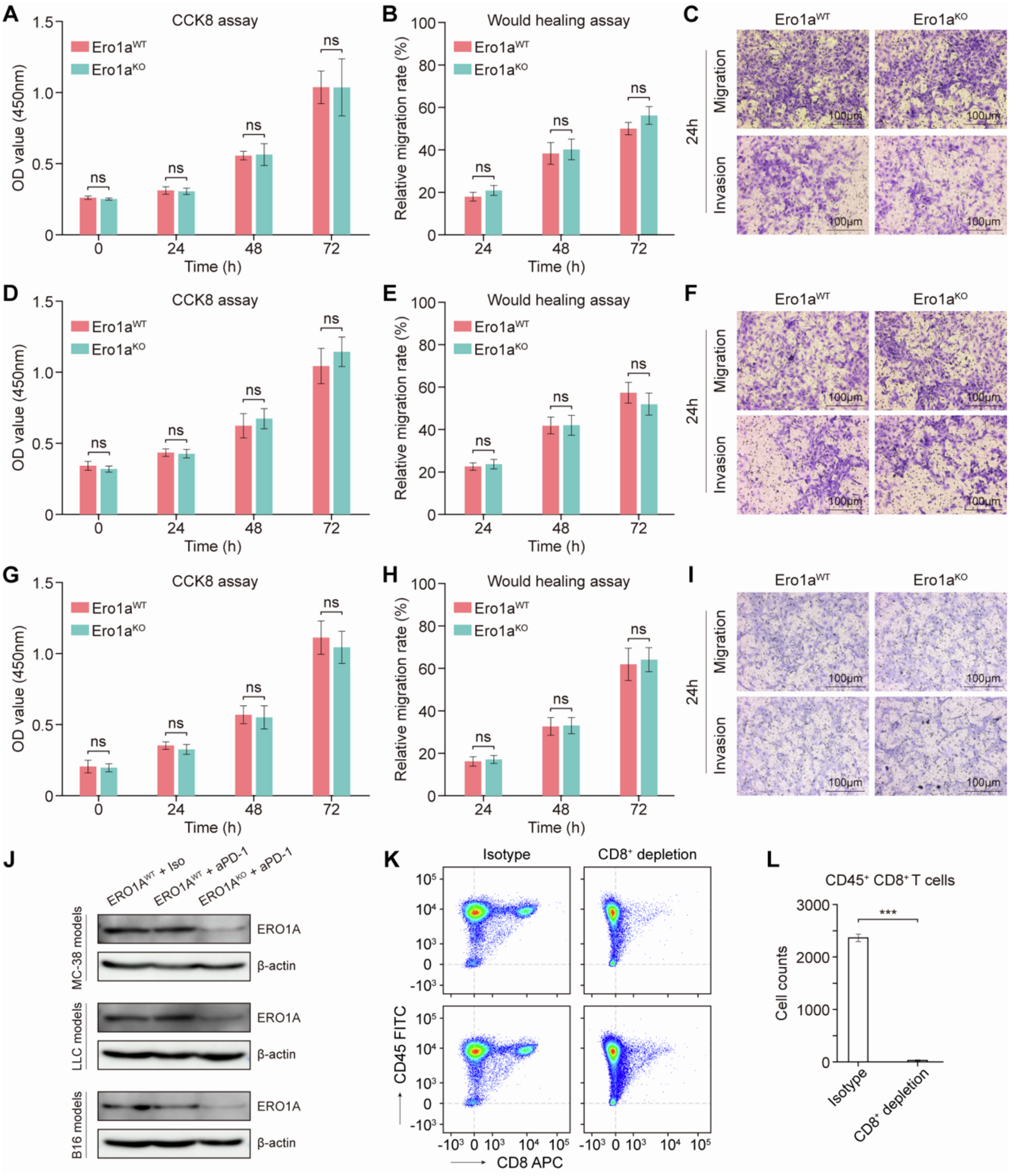

Figure S1: ERO1A deficiency affects the anti-tumor efficacy of immunotherapy but not the proliferation of tumor cells, related to Figure 1.

(A-C) Deletion of *Ero1a* in MC-38 cells did not impede cell proliferation (A), migration (B), and invasion (C) *in vitro* (n = 3 independent repeats). Data presented as means ± SEMs. ns, not significant. Two-sided Student's t test. Scale bars, 100 μm.

(D-F) Deletion of *Ero1a* in B16 cells did not impede cell proliferation (D), migration (E), and invasion (F) *in vitro* (n = 3 independent repeats). Data presented as means ± SEMs. ns, not significant. Two-sided Student's t test. Scale bars, 100 μm.

(G-I) Deletion of *Ero1a* in LLC cells did not impede cell proliferation (G), migration (H), and invasion

(I) *in vitro* (n = 3 independent repeats). Data presented as means ± SEMs. ns, not significant. Two-sided Student's t test. Scale bars, 100 μm.

(J) Western blotting plots of ERO1A expression levels in MC-38, LLC, and B16 therapeutic models. Result is a representative finding for 3 experiments.

(K and L) Flow cytometry (FCM) analysis of periphery blood from immunocompetent C57 mice after depletion of $CD8^+$ via antibody-based approaches. FCM plots of $CD8^+$ T cells in tumor-bearing mice treated with anti-$CD8^+$ antibody or isotype control (K). Box plot showing the quantitative cell numbers of $CD8^+$ T cells based on the FCM analysis (L). Representative of n = 3 mice/group. Data presented as means ± SEMs. ***$p < 0.001$. Two-sided Student's t test.

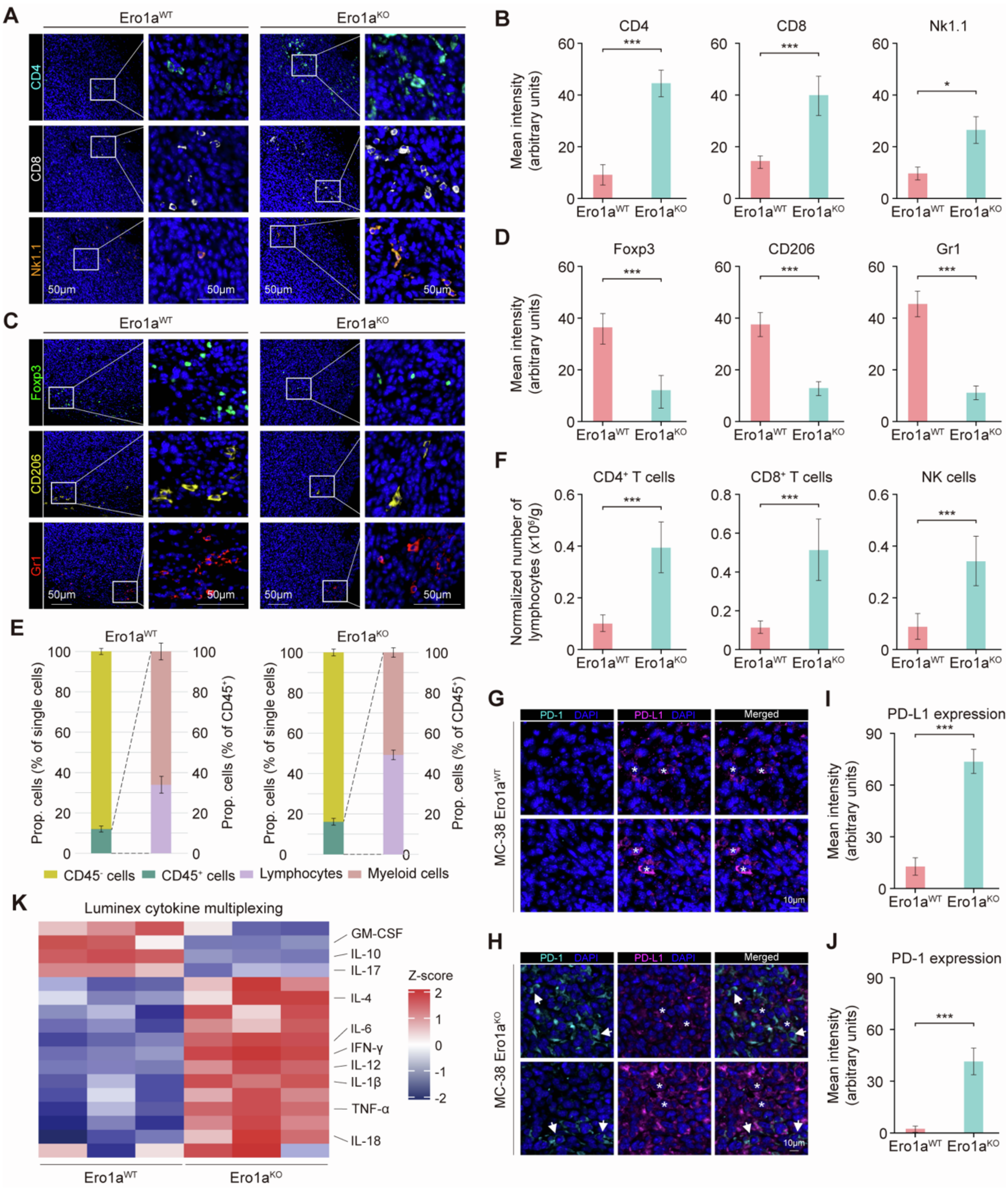

Figure S2: Immunophenotyping of ERO1A^{WT} and ERO1A^{KO} tumors in MC-38 therapeutic models, related to Figure 1.

(A and B) Immunofluorescence (IF) staining of CD4^{+} T cells (cyan), CD8^{+} T cells (white), Nk1.1^{+} NK cells (orange), and DAPI (blue) in the core region of Ero1a^{WT} and Ero1a^{KO} tumors (A). Box plots comparing the mean intensity of CD4^{+} T cells, CD8^{+} T cells, and NK cells in Ero1a^{WT} and Ero1a^{KO} tumors based on the IF staining (B). Data presented as means ± SDs from 6 randomly selected fields. *p < 0.05, ***p < 0.001. Two-sided Student's t test. Scale bars, 50 μm.

(C and D) IF staining of Foxp3^{+} Tregs (green), CD206^{+} macrophages (yellow), Gr1^{+} MDSCs (red), and DAPI (blue) in the core region of Ero1a^{WT} and Ero1a^{KO} tumors (C). Box plots comparing the mean

intensity of $Foxp3^+$ Tregs, $CD206^+$ macrophages, and $Gr1^+$ MDSCs in $Ero1a^{WT}$ and $Ero1a^{KO}$ tumors based on the IF staining (D). Data presented as means ± SDs from 6 randomly selected fields. ***p < 0.001. Two-sided Student's t test. Scale bars, 50 μm.

(E and F) FCM analysis of $CD45^+$ immunocytes, lymphocytes, and myeloid cells in $Ero1a^{WT}$ and $Ero1a^{KO}$ tumors (E). Box plots showing the quantitative cell numbers of tumor-infiltrating lymphocytes normalized by tumor weight, including $CD4^+$ T, $CD8^+$ T, and NK cells, based on FCM analysis (F). Representative of n = 6 mice/group. Data presented as means ± SEMs. ***p < 0.001. Two-sided Student's t test.

(G and H) IF staining of PD-L1 (violet), PD-1 (cyan) and DAPI (blue) in $Ero1a^{WT}$ (G) and $Ero1a^{KO}$ tumors (H). Tumors were collected after 2 rounds of aPD-1 treatment. Data represented for 3 randomly selected fields. Scale bars, 10 μm.

(I and J) Box plots comparing the mean intensity of PD-L1 (I) and PD-1 (J) in $Ero1a^{WT}$ and $Ero1a^{KO}$ tumors based on the IF staining. Data presented as means ± SDs from 6 randomly selected fields. ***p < 0.001. Two-sided Student's t test.

(K) Heatmap showing the secretion levels of cytokines/chemokines in $Ero1a^{WT}$ and $Ero1a^{KO}$ tumors, measured by Luminex-based multiplexing (n = 3 mice/group).

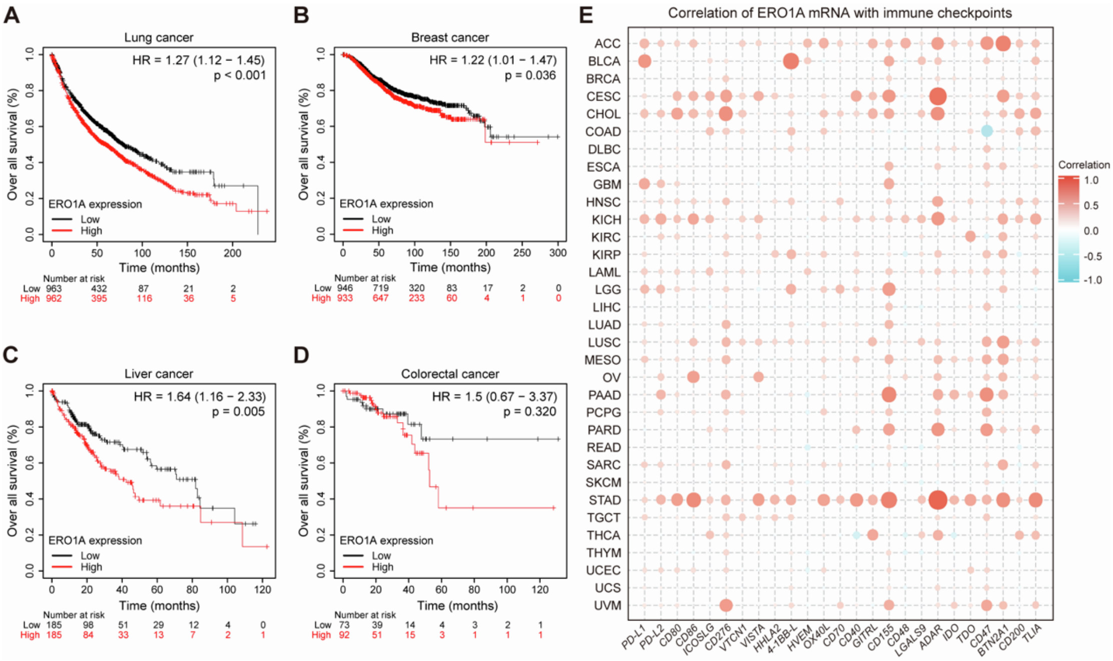

Figure S3: Correlation between ERO1A expression and clinical outcomes in patients with solid tumors, related to Figure 2.

(A-D) Survival curves showing that ERO1A was associated with worse overall survival (OS) of TCGA patients with lung cancer (A), breast cancer (B), liver cancer (C), and colorectal cancer (D). Log-rank test.

(E) Scatter plots showing the correlations of ERO1A mRNA and expressions of immune checkpoints in pan-cancers, according to the TCGA-based analyses. ACC, adrenocortical carcinoma. BLCA, bladder urothelial carcinoma. BRCA, breast invasive carcinoma. CESC, cervical squamous cell carcinoma. CHOL, cholangio carcinoma. COAD, colon adenocarcinoma. DLBC, diffuse large B-cell lymphoma. ESCA, esophageal carcinoma. GBM, glioblastoma multiforme. HNSC, head and neck squamous cell carcinoma. KICH, kidney chromophobe. KIRC, kidney renal clear cell carcinoma. KIRP, kidney renal papillary cell carcinoma. LAML, acute myeloid leukemia. LGG, brain lower grade glioma. LIHC, liver hepatocellular carcinoma. LUAD, lung adenocarcinoma. LUSC, lung squamous cell carcinoma. MESO, mesothelioma. OV, ovarian serous cystadenocarcinoma. PAAD, pancreatic adenocarcinoma. PCPG, pheochromocytoma and paraganglioma. PRAD, prostate adenocarcinoma. READ, rectum adenocarcinoma. SARC, sarcoma. SKCM, skin cutaneous melanoma. STAD, stomach adenocarcinoma. TGCT, testicular germ cell tumors. THCA, thyroid carcinoma. THYM, thymoma. UCEC, uterine corpus endometrial carcinoma. UCS, uterine carcinosarcoma. UVM, uveal melanoma. Pearson correlation test.

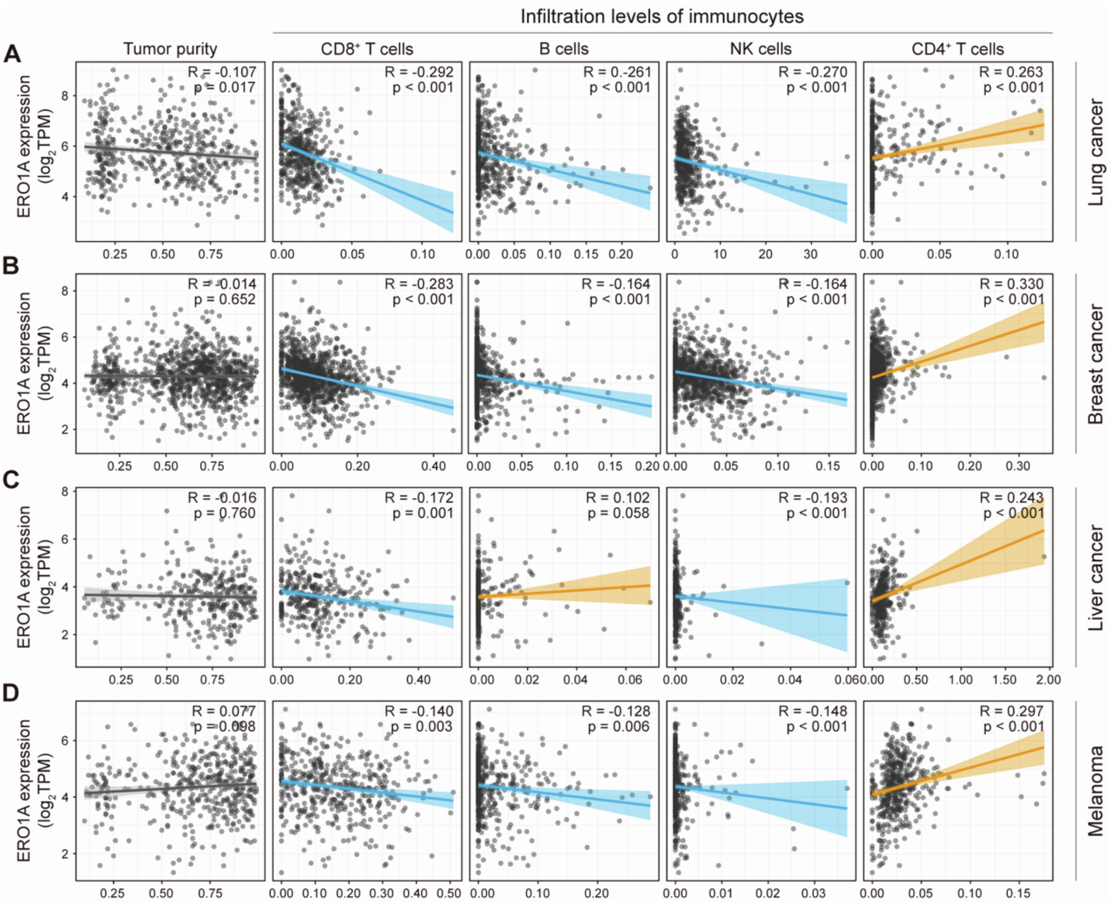

Figure S4: ERO1A is correlated with immune-suppressive tumor microenvironment in patients with solid tumors, related to Figure 2.

(A) Scatter plots showing correlations of ERO1A expression with $CD4^+$ T cells, $CD8^+$ T cells, B cells, and NK cells in TCGA LUAD data based on their signature gene expressions. Pearson correlation test.

(B) Scatter plots showing correlations of ERO1A expression with $CD4^+$ T cells, $CD8^+$ T cells, B cells, and NK cells in TCGA BRCA data based on their signature gene expressions. Pearson correlation test.

(C) Scatter plots showing correlations of ERO1A expression with $CD4^+$ T cells, $CD8^+$ T cells, B cells, and NK cells in TCGA LIHC data based on their signature gene expressions. Pearson correlation test.

(D) Scatter plots showing correlations of ERO1A expression with $CD4^+$ T cells, $CD8^+$ T cells, B cells, and NK cells in TCGA SKCM data based on their signature gene expressions. Pearson correlation test.

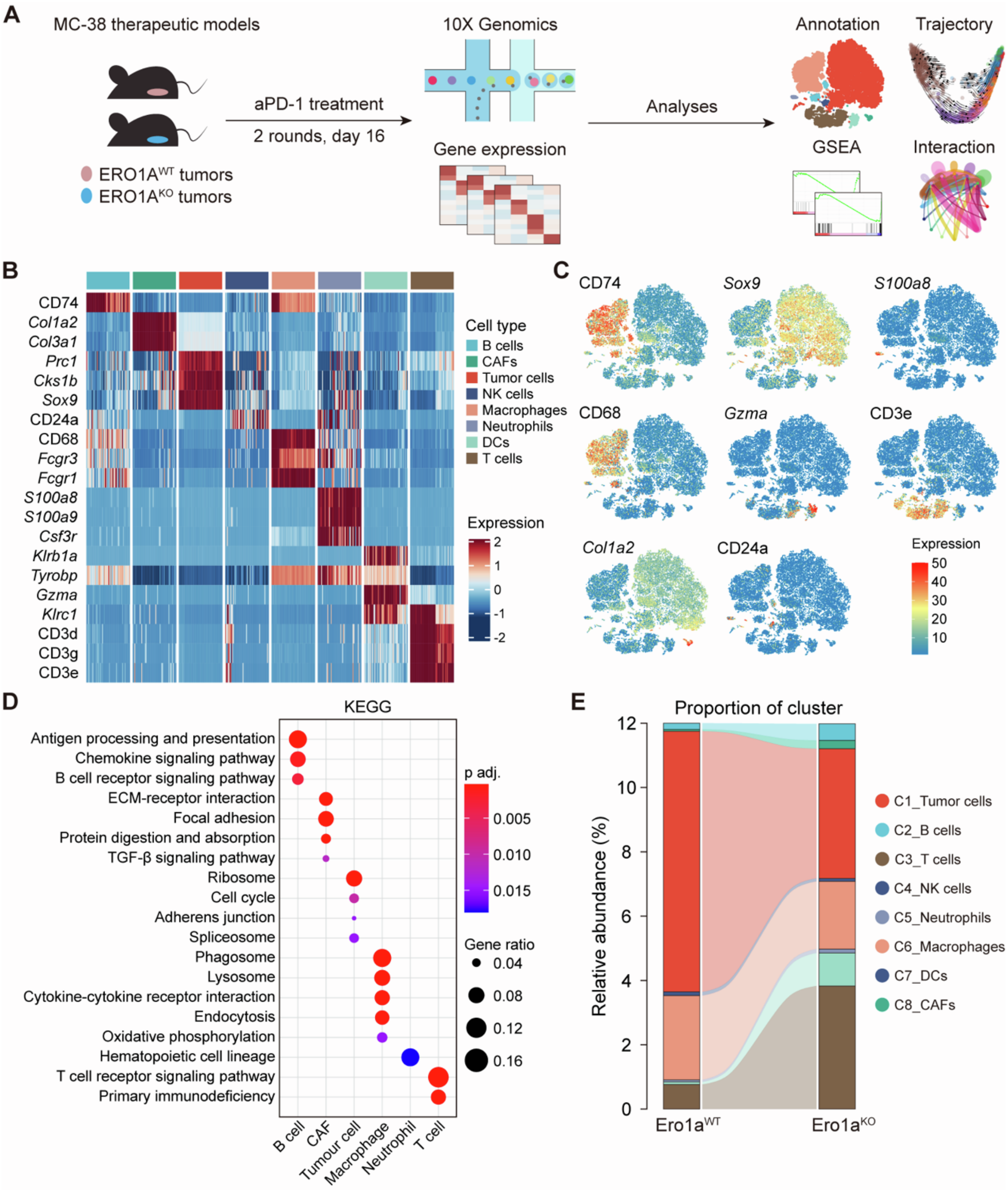

Figure S5: Single-cell RNA-seq analyses of cell populations in ERO1A^{WT} and ERO1A^{KO} tumors treated with aPD-1 blockade, related to Figure 2.

(A) Schematic overview of the scRNA-seq experimental design. Tumors were transplanted on day 0 and aPD-1 treatment was administered every 3 days from day 10. Ero1a^{WT} and Ero1a^{KO} tumors from MC-38 therapeutic models were collected after 2 rounds of aPD-1 treatment on day 16.

(B) Heatmap showing the expression levels of marker genes across major cell types, measured by scRNA-seq. CAFs, cancer-associated fibroblasts. DCs, dendritic cells. NK, natural killer.

(C) t-SNE map showing the marker gene expressions of major cell types based on the scRNA-seq data. Colored by cell subtypes.

(D) KEGG enrichment plot of major cell types based on scRNA-seq data.

(E) Sankey plot comparing the relative abundance (%) of major cell types between Ero1a^{WT} and Ero1a^{KO} tumors based on scRNA-seq data, colored by cell subtypes. CAFs, cancer-associated fibroblasts. DCs, dendritic cells. NK, natural killer.

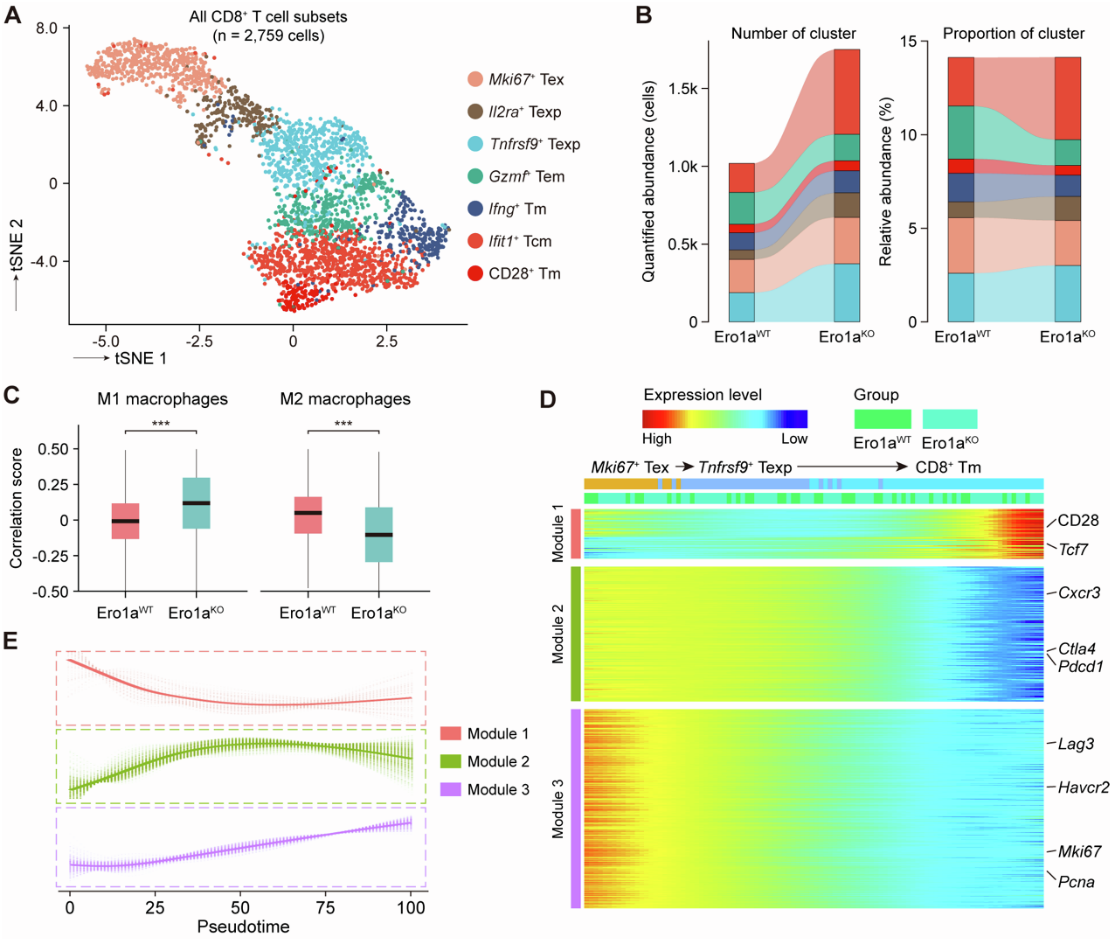

Figure S6: Immunophenotyping of ERO1A-associated tumor microenvironment remodeling in response to immunotherapy, related to Figure 2.

(A) t-SNE map showing the $CD8^+$ T cell clusters based on the scRNA-seq data. Colored by cell subtypes.

(B) Sankey plots comparing the quantified numbers (cells) relative abundance (%) of major cell types between $ERO1A^{WT}$ and $ERO1A^{KO}$ tumors based on scRNA-seq data, colored by cell subtypes.

(C) Box plots comparing the correlation scores of M1 and M2 macrophage signatures in $Ero1a^{WT}$ and $Ero1a^{KO}$ tumors based on their signature gene expressions, measured by scRNA-seq. Data presented as means ± SEMs. ***$p < 0.001$. Two-sided Student's t test.

(D and E) Trajectory analysis of $CD8^+$ T cells based on the scRNA-seq data in MC-38 therapeutic models. The dynamically expressed gene on the trajectory of $CD8^+$ T cells in $Ero1a^{WT}$ and $Ero1a^{KO}$ tumors were identified and group into 3 modules (D). The pseudotime expression changes of each module in $CD8^+$ T cells (E).

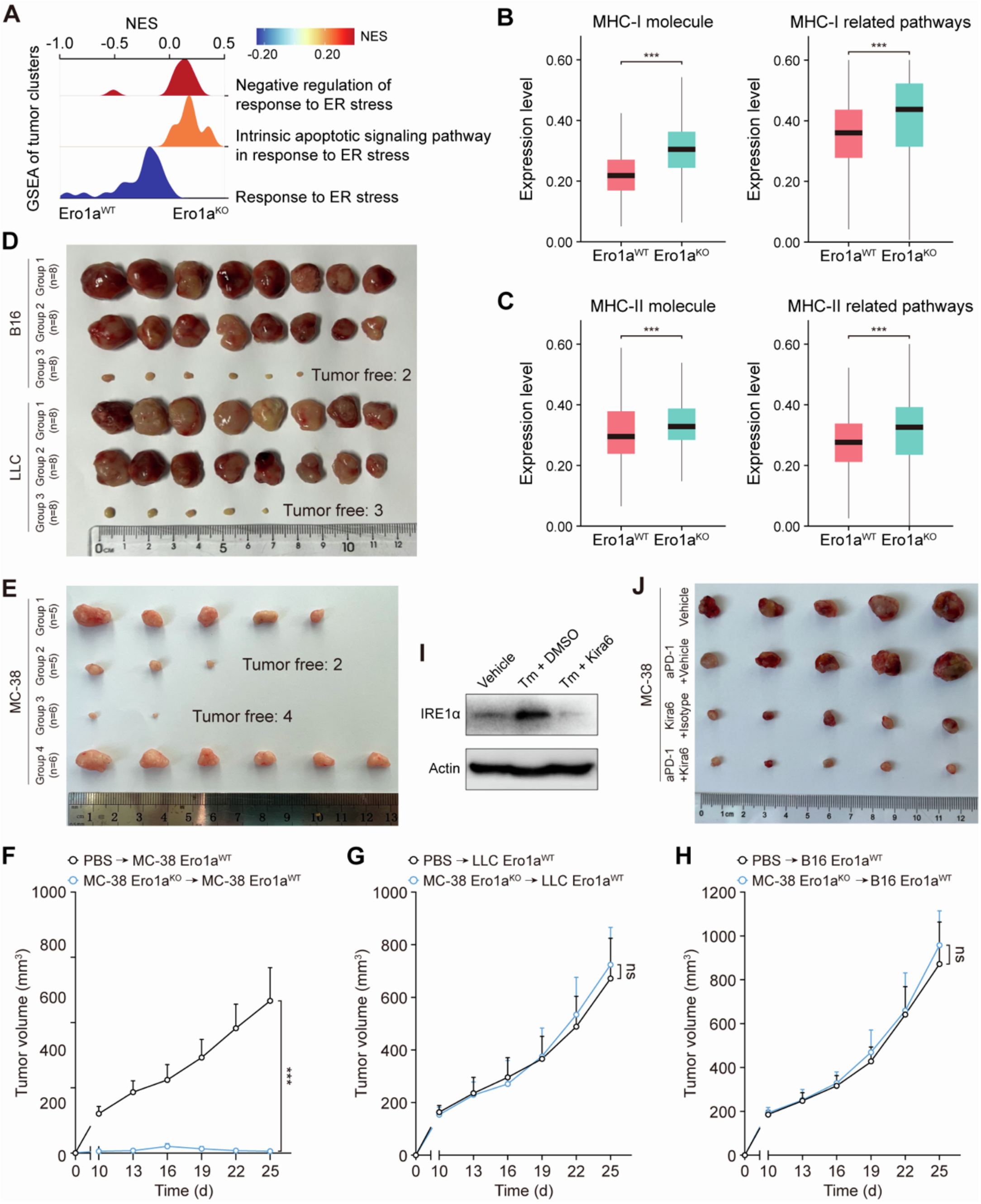

Figure S7: ERO1A deficiency triggers immunogenic cell death due to unresolved ER stress, related to Figures 3 and 4.

(A) GSEA of tumor cell subsets showing higher enrichment of negative regulation of ER stress response and apoptotic signaling pathway in Ero1a^{KO} tumors, compared to those in Ero1a^{WT} tumors.

(B) Box plots showing the comparisons of MHC-I molecule expression and MHC-I related pathways in Ero1a^{WT} and Ero1a^{KO} tumors, based on the scRNA-seq data in MC-38 therapeutic models. Data presented as means ± SEMs. ***p < 0.001. Two-sided Student's t test.

(C) Box plots showing the comparisons of MHC-II molecule expression and MHC-II related pathways in Ero1a^{WT} and Ero1a^{KO} tumors, based on the scRNA-seq data in MC-38 therapeutic models. Data

presented as means ± SEMs. ***$p < 0.001$. Two-sided Student's t test.
(D) Right flank tumor growth from rechallenged mice bearing B16 $Ero1a^{WT}$ (upper) or LLC $Ero1a^{WT}$ tumors (bottom). First challenge was with PBS (Group 1), $Ero1a^{WT}$ (Group 2), or $Ero1a^{KO}$ (Group 3) tumor cells on the left flank of C57CL/6 mice and rechallenge was performed after 10 days with $Ero1a^{WT}$ tumor cells on the right flank (n = 8 mice/group).
(E) Right flank tumor growth from rechallenged mice bearing MC-38 $Ero1a^{WT}$ tumors. First challenge was with PBS (Group 1, n = 5), $Ero1a^{WT}$ (Group 2, n = 5), $Ero1a^{KO}$ (Group 3, n = 6), or $Ero1a^{OE}$ (Group 4, n = 6) tumor cells on the left flank of C57CL/6 mice and rechallenge was performed after 10 days with MC-38 $Ero1a^{WT}$ tumor cells on the right flank.
(F) Tumor volume in mice bearing MC-38 $Ero1a^{WT}$ tumors in naïve mice (PBS pre-treated) or mice that had previously injected with MC-38 $Ero1a^{KO}$ cells (n = 6 mice/group). Data presented as means ± SEMs. ***$p < 0.001$. Two-sided Student's t test.
(G and H) Tumor volumes in mice bearing LLC $Ero1a^{WT}$ (G) and B16 $Ero1a^{WT}$ (H) tumors in naïve mice (PBS pre-treated) or mice that had previously injected with MC-38 $Ero1a^{KO}$ cells (n = 6 mice/group). Data presented as means ± SEMs. ns, not significant. Two-sided Student's t test.
(I) Western blotting plots of IRE1α in MC-38 $Ero1a^{WT}$ tumor cells treated with Kira6 *in vitro*. Result is a representative finding for 3 experiments.
(J) Tumor growth in C57BL/6 mice bearing MC-38 $Ero1a^{WT}$ tumors, treated with vehicle, aPD-1 plus vehicle, Kira6 plus isotype, or Kira6 plus aPD-1 blockade (n = 5 mice/group).

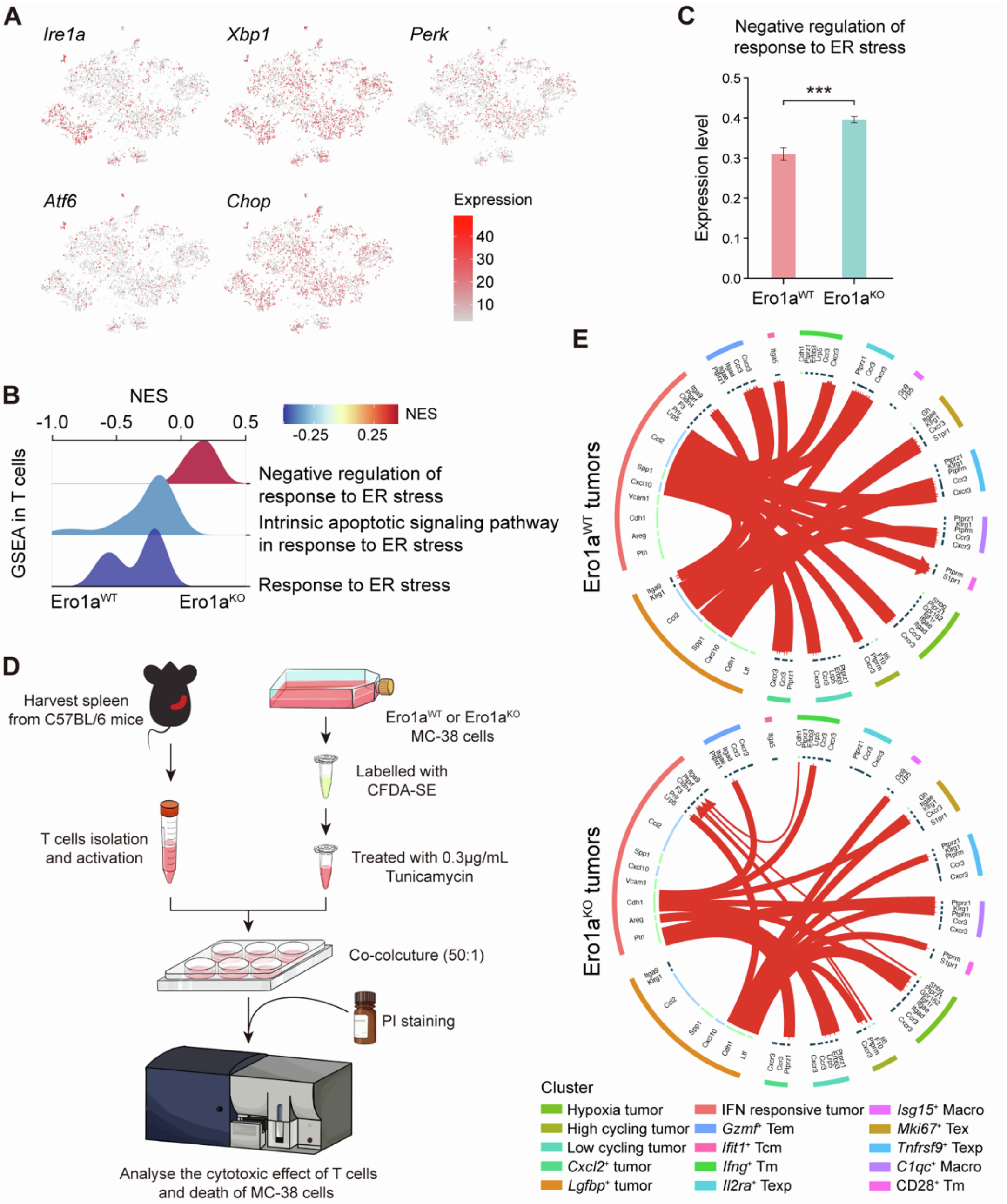

Figure S8: ERO1A induces T cell dysfunction by transmissible ER stress, related to Figure 5.

(A) t-SNE map of unfolded protein response-related genes in T cells, based on the scRNA-seq data in MC-38 therapeutic models. Colored by expression levels.

(B) GSEA of T cell subsets showing lower enrichment of response to ER stress response and intrinsic apoptotic signaling pathway in T cells from Ero1a^{KO} tumors compared with counterpart from WT controls, based on the scRNA-seq data in MC-38 therapeutic models.

(C) Bar plot comparing the signature of negative regulation of response to ER stress in Ero1a^{WT} and Ero1a^{KO} tumors, based on the scRNA-seq data in MC-38 therapeutic models. Data presented as means ± SDs. ***p < 0.001. Two-sided Student's t test.

(D) Schematic diagram of the T cell cytotoxic functional assay.

(E) Cell-to-cell interaction analyses within Ero1a^{WT} or Ero1a^{KO} tumors by iTALK algorithm, based on the scRNA-seq data in MC-38 therapeutic models.

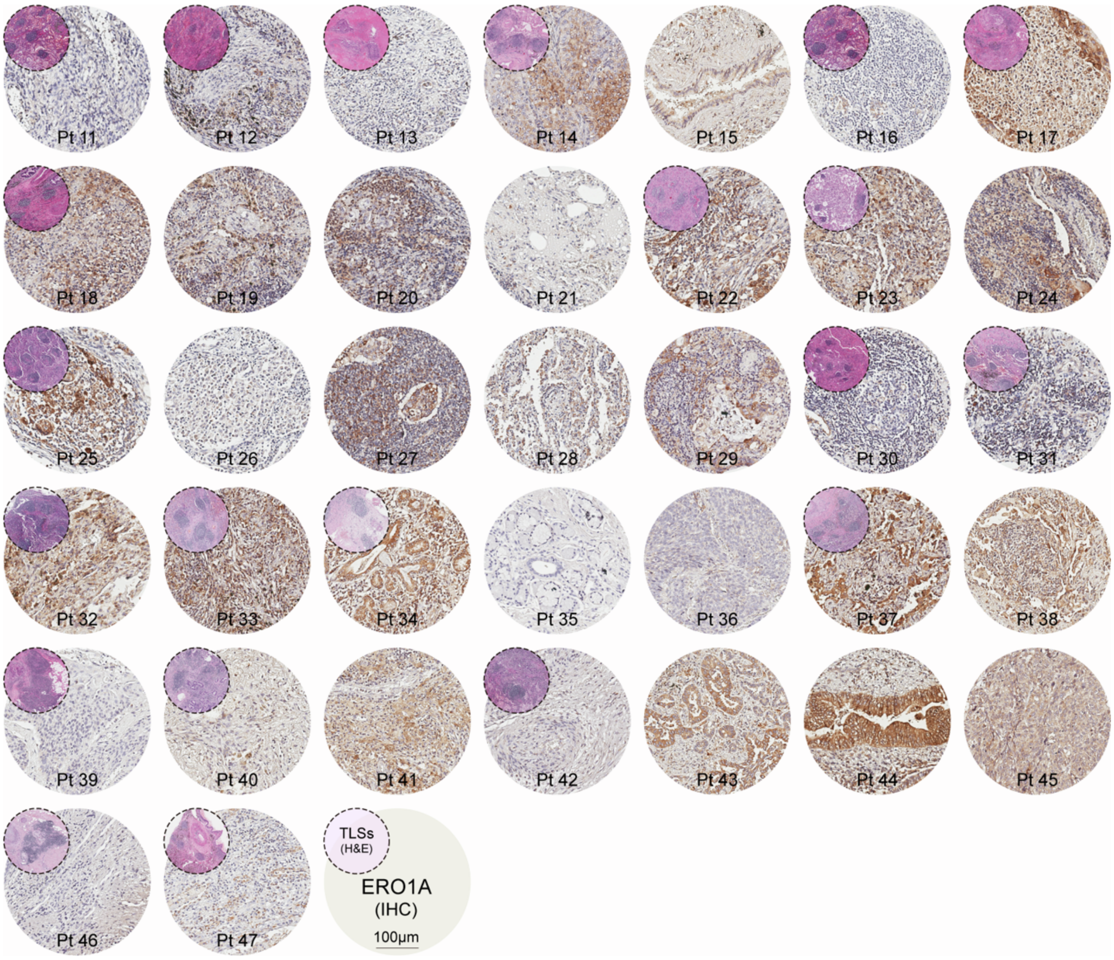

Figure S9: ERO1A expression levels in the included NSCLC patients, related to Figure 6. Representative immunohistochemistry staining of ERO1A in the 37 lung tumors treated with neoadjuvant immunotherapy. IHC staining distinguished 15 patients with low ERO1A expression (Pt 11, Pt 12, Pt 13, Pt 16, Pt 21, Pt 26, Pt 30, Pt 31, Pt 35, Pt 36, Pt 39, Pt 40, Pt 42, Pt 46, and Pt 47) and 22 patients with high ERO1A expression (Pt 14, Pt 15, Pt 17, Pt 18, Pt 19, Pt 20, Pt 22, Pt 23, Pt 24, Pt 25, Pt 27, Pt 28, Pt 29, Pt 32, Pt 33, Pt 34, Pt 37, Pt 38, Pt 41, Pt 43, Pt 44, and Pt 45). Tertiary lymphoid structures are identified by H&E staining and denoted by dotted line. IHC, immunohistochemistry. TLSs, tertiary lymphoid structures. Scale bar = 100μm.

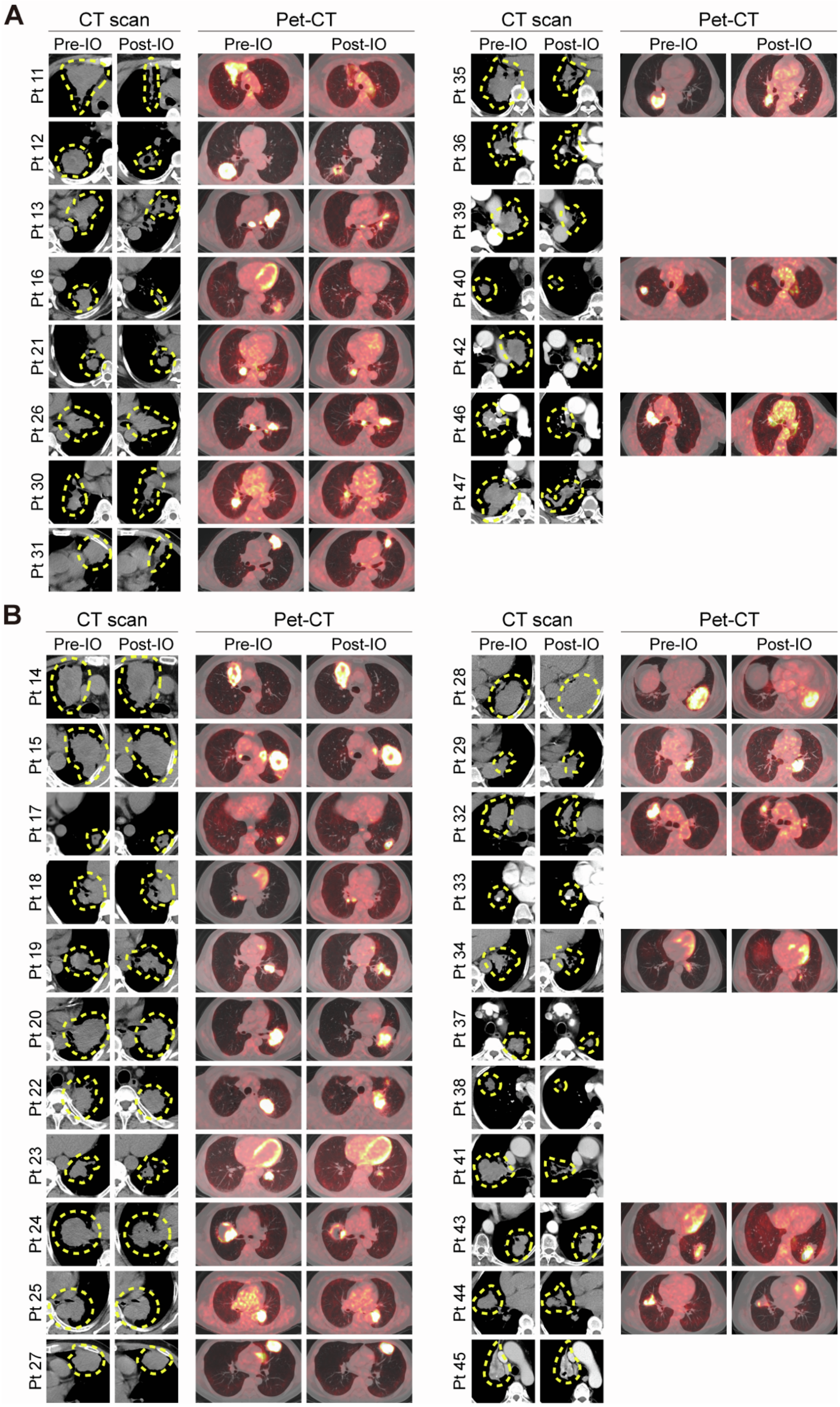
A
CT scan
Pet-CT
Pre-IO
Post-IO
Pre-IO
Post-IO
CT scan
Pet-CT
Pre-IO
Post-IO
Pre-IO
Post-IO
Pt 11
Pt 12
Pt 13
Pt 16
Pt 21
Pt 26
Pt 30
Pt 31
Pt 35
Pt 36
Pt 39
Pt 40
Pt 42
Pt 46
Pt 47
B
CT scan
Pet-CT
Pre-IO
Post-IO
Pre-IO
Post-IO
CT scan
Pet-CT
Pre-IO
Post-IO
Pre-IO
Post-IO
Pt 14
Pt 15
Pt 17
Pt 18
Pt 19
Pt 20
Pt 22
Pt 23
Pt 24
Pt 25
Pt 27
Pt 28
Pt 29
Pt 32
Pt 33
Pt 34
Pt 37
Pt 38
Pt 41
Pt 43
Pt 44
Pt 45

Figure S10: ERO1A attenuates anti-tumor efficacy of aPD-1 blockade in patients with lung cancer, related to Figure 6.

(A) Computed tomography (CT) scan images and available corresponding PET-CT scan images of the 15 patients in ERO1A^{low} group. CT scan was performed before (pre-IO) and after immunotherapy (pos-IO), respectively. IO, immunotherapy. Tumor is denoted by dotted line.

(B) Computed tomography (CT) scan images and available corresponding PET-CT scan images of the 22 patients in ERO1A^{high} group. CT scan was performed before (pre-IO) and after immunotherapy (pos-IO), respectively. IO, immunotherapy. Tumor is denoted by dotted line.

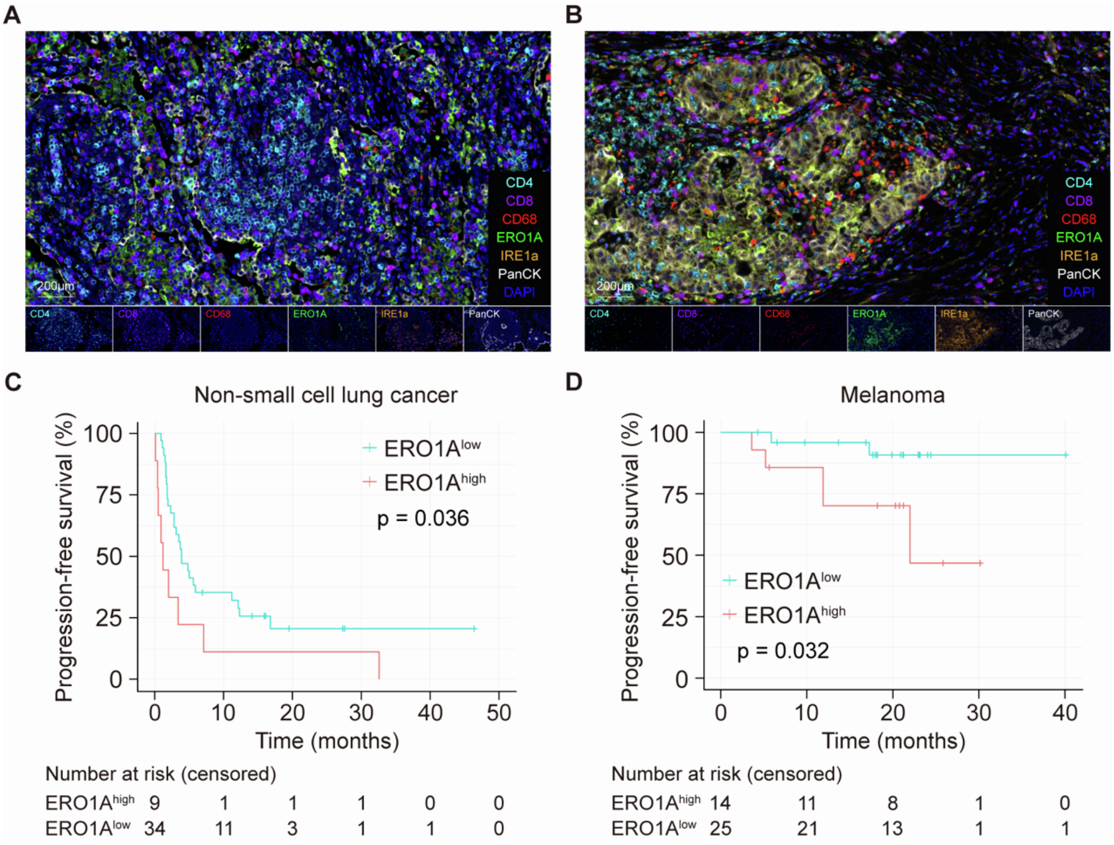

Figure S11: ERO1A as a biomarker in patients treated with immunotherapy, related to Figure 6. (A and B) Multiplex IHC (mIHC) staining of CD4 (cyan), CD8 (violet), CD68 (red), ERO1A (green), IRE1a (orange), PanCK (white), and DAPI (blue) of lung tumors treated with neoadjuvant immunotherapy. Representative mIHC staining of resected tumor samples from patients with ERO1A low (A) or ERO1A high (B) expression (n = 3 patients). Scale bars, 200 μm.

(C) Progression-free survival (PFS) for NSCLC patients stratified by the ERO1A expression based on the GEO: GSE190265 dataset. Kaplan-Meier curves of PFS for ERO1A high (n = 9 patients) and low (n = 34 patients). Log-rank test.

(D) PFS for melanoma patients stratified by the ERO1A expression based on the ENA: PRJEB23709 dataset. Kaplan-Meier curves of PFS for ERO1A high (n = 14 patients) and low (n = 25 patients). Log-rank test.

Table S1. Primers used for RT-qPCR, related to the STAR Methods.

Gene	NCBI ID	Size (BP)	Sequence	Tm (℃)	GC (%)
β-actin	NM_007393.3	287	F: GTGACGTTGACATCCGTAAAGA	58.7	45.5
			R: GTAACAGTCCGCCTAGAAGCAC	61	54.6
Ire1a	NM_023913.2	166	F: GAGAATCAGACGAGCACCCAA	60.07	52.38
			R: GTCTGATGAAGCAGGGTGATGG	61	54.55
Xbp1	NM_001271730.1	178	F: ATTCTGAGTCTGATATCCTTTTGGG	58.4	40
			R: TCCAGCTTGGCTGATGAGGT	61.2	55
Atf4	NM_001287180.1	126	F: AGACACCGGCAAGGAGGATG	61.9	60
			R: AAGAGCTCATCTGGCATGGTTT	60.29	45.45
Atf6	M_001081304.1	133	F: CAAATAGCCAACAGAAAGCCC	57.76	47.62
			R: TAATACACTTGCAGCTCACTCCC	60.37	47.83
Ddit3	NM_001290183.1	133	F: CTGCCTTTCACCTTGGAGACG	61.21	57.14
			R: TTGATTCTTCCTCTTCGTTTCCTG	59	41.67
Perk	NM_001313918.1	85	F: AGTGGGATTTGGACGTGGG	59.62	57.89
			R: TCTTTTGAGGAAGTTTTGTGGGTG	59.84	41.67
