# Supplementary material for: Ablation of ERO1A induces lethal endoplasmic reticulum stress responses and immunogenic cell death to activate anti-tumor immunity
Source: Cell Rep Med. 2023 Sep 27;4(10):101206. doi: 10.1016/j.xcrm.2023.101206 (PMC10591028; doi:10.1016/j.xcrm.2023.101206)
Supplement: Document S2. Article plus supplemental information [file mmc2.pdf]

Cell Reports
Medicine

Article

Ablation of ERO1A induces lethal endoplasmic reticulum stress responses and immunogenic cell death to activate anti-tumor immunity

Graphical abstract

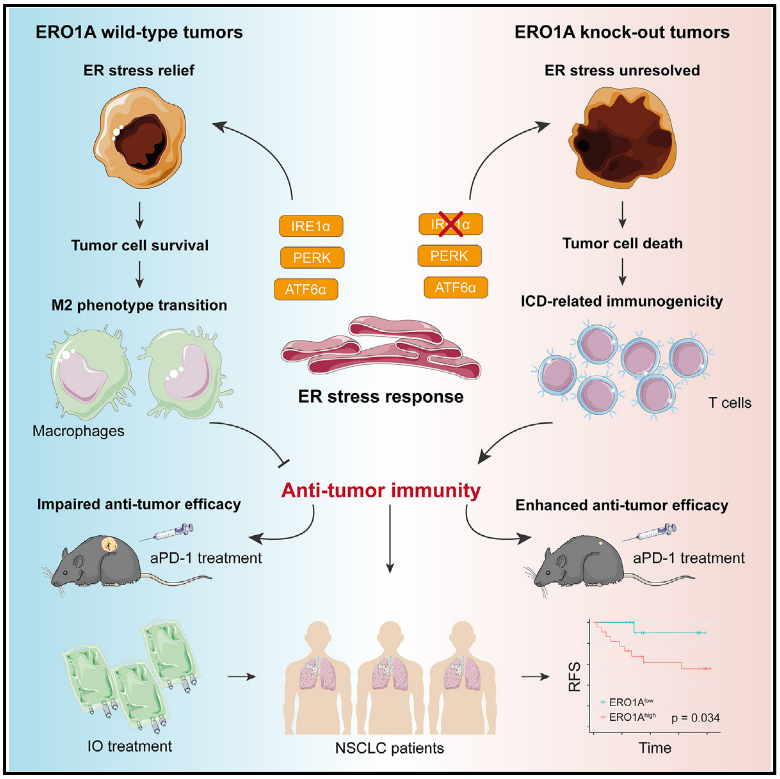

Highlights

- ERO1A induces immunosuppression and resistance to PD-1 blockade
- ERO1A impairs the balance between IRE1α and PERK signaling activities
- Ablation of tumor ERO1A enhances anti-tumor immunity via immunogenic cell death
- ERO1A is a potential target for therapeutic strategies in cancer immunotherapy

Authors

Lihui Liu, Sini Li, Yan Qu, ..., Xue Zhang, Zixiao Ma, Jie Wang

Correspondence

zlhuxi@163.com

In brief

Liu et al. reveal that ERO1A mediates an immune-suppressive TME and attenuates the response to PD-1 blockade by impairing the balance between IRE1α and PERK signaling activities and induces lethal unfolded protein responses. They find that targeting ERO1A can incite anti-tumor immunity and enhance aPD-1 efficacy in therapeutic models.

Liu et al., 2023, Cell Reports Medicine *4*, 101206
October 17, 2023 © 2023 The Author(s).
https://doi.org/10.1016/j.xcrm.2023.101206

CellPress

Cell Reports Medicine

CellPress
OPEN ACCESS

Article

Ablation of ERO1A induces lethal endoplasmic reticulum stress responses and immunogenic cell death to activate anti-tumor immunity

Lihui Liu,[1,2,7] Sini Li,[1,2,3,7] Yan Qu,[1,2,4,7] Hua Bai,[1,2,7] Xiangyu Pan,[5] Jian Wang,[5] Zhijie Wang,[1,2] Jianchun Duan,[1,2] Jia Zhong,[1,2] Rui Wan,[1,2] Kailun Fei,[1,2] Jiachen Xu,[1,2] Li Yuan,[1,2] Chao Wang,[1,2] Pei Xue,[6] Xue Zhang,[1,2] Zixiao Ma,[1,2] and Jie Wang[1,2,8,*]

[1]State Key Laboratory of Molecular Oncology, Department of Medical Oncology, National Cancer Center/National Clinical Research Center for Cancer/Cancer Hospital, Chinese Academy of Medical Sciences and Peking Union Medical College, Beijing 100021, China

[2]CAMS Key Laboratory of Translational Research on Lung Cancer, State Key Laboratory of Molecular Oncology, Department of Medical Oncology, National Cancer Center/National Clinical Research Center for Cancer/Cancer Hospital, Chinese Academy of Medical Sciences and Peking Union Medical College, Beijing 100021, China

[3]Department of Medical Thoracic Oncology, Zhejiang Cancer Hospital, Hangzhou Institute of Medicine (HIM), Chinese Academy of Sciences, Hangzhou, Zhejiang 310022, China

[4]Department of Radiotherapy, Shandong Provincial Hospital Affiliated to Shandong First Medical University, Jinan, Shandong 250021, China

[5]State Key Laboratory of Biotherapy and Cancer Center, West China Hospital, Sichuan University, Chengdu, Sichuan 610041, China

[6]Department of Surgical Sciences, Sleep Science Laboratory (BMC), Uppsala University, Uppsala, Sweden

[7]These authors contributed equally

[8]Lead contact

*Correspondence: zlhuxi@163.com

https://doi.org/10.1016/j.xcrm.2023.101206

SUMMARY

Immunophenotyping of the tumor microenvironment (TME) is essential for enhancing immunotherapy efficacy. However, strategies for characterizing the TME exhibit significant heterogeneity. Here, we show that endoplasmic reticular oxidoreductase-1α (ERO1A) mediates an immune-suppressive TME and attenuates the response to PD-1 blockade. Ablation of ERO1A in tumor cells substantially incites anti-tumor T cell immunity and promotes the efficacy of aPD-1 in therapeutic models. Single-cell RNA-sequencing analyses confirm that ERO1A correlates with immunosuppression and dysfunction of $CD8^+$ T cells along anti-PD-1 treatment. In human lung cancer, high ERO1A expression is associated with a higher risk of recurrence following neoadjuvant immunotherapy. Mechanistically, ERO1A ablation impairs the balance between IRE1α and PERK signaling activities and induces lethal unfolded protein responses in tumor cells undergoing endoplasmic reticulum stress, thereby enhancing anti-tumor immunity via immunogenic cell death. These findings reveal how tumor ERO1A induces immunosuppression, highlighting its potential as a therapeutic target for cancer immunotherapy.

INTRODUCTION

Immune checkpoint inhibitors (ICIs) have shifted the paradigm of cancer treatment; however, many patients have experienced low or no clinical response after immunotherapeutic interventions.[1] Better biomarkers for predicting clinical response and novel immunotherapeutic targets for more effective combination treatments are needed to overcome immune resistance in the era of "immunotherapy 2.0".

It has recently been proposed that tumors act as an ecosystem, where the host extensively interacts with the tumor microenvironment (TME), the distal metastatic environment, and its internal environment.[2] With increasing knowledge about TME, the influence of its complexity and diversity on immunotherapy response has been fully evaluated.[3,4] Clinical trials have revealed that "hot" tumors (immune inflamed with high $CD8^+$ T cell infiltration) respond to ICIs, whereas "cold" tumors (immune desert/exclusive with low $CD8^+$ T cell infiltration) require co-treatment to improve the anti-tumor efficacy.[3,5] However, the diversity of immune evasion mechanisms is the primary obstacle in converting non-responsive cold tumors into responsive hot tumors.[6] Therefore, exploring the mechanisms of such transitions and tumor immunotyping can provide significant insights into designing precision strategies against tumors.

The hostile TME conditions, including hypoxia, nutrient deprivation, exposure to therapeutic agents, and immune responses, perturb the protein-folding capacity of the endoplasmic reticulum (ER), thereby provoking ER stress in cells.[7] To reinstate ER homeostasis and its protein-folding capacity transcriptionally and translationally, tumor cells activate an adaptive ER stress response known as the unfolded protein response (UPR).[7,8] ER stress *in situ*, coordinated by the activation of inositol-requiring

© 2023 The Author(s).
This is an open access article under the CC BY license (http://creativecommons.org/licenses/by/4.0/).

enzyme-1α (IRE1α) and PRKR-like ER kinase (PERK), facilitates tumor growth and drug resistance and governs multiple pro-tumoral attributes by reprogramming the function of immune infiltrates.[7–9] By contrast, unresolved or extreme ER stress induced by drugs or immune responses has been shown to trigger immunogenic cell death (ICD), thereby evoking protective anti-tumor immunity.[10] However, the association of ER-stress-induced tumor cell signaling with immune-regulatory programs remains poorly understood.

During ER stress, the expression of endoplasmic reticular oxidoreductase-1α (ERO1A) increases, contributing to tumor cell survival.[11,12] Furthermore, PERK activation upregulates ERO1A and facilitates protein folding, alleviating ER stress and thus maintaining tumor survival.[13,14] Our previous study showed that intrinsic activation of tumor ERO1A drives immunosuppression by recruiting regulatory T cells, cancer-associated fibroblasts, and myeloid-derived suppressor cells (MDSCs) in lung cancer.[15] However, the mechanism by which ERO1A regulates host immunity remains undetermined.

Here, we investigate the crosstalk between ERO1A activation and TME remodeling in solid tumors. Disruption of ERO1A impairs the balance between IRE1α and PERK signaling activities and triggers lethal UPR in ER-stressed tumor cells, thereby promoting host anti-tumor immunity via ICD. Furthermore, a high expression level of ERO1A is associated with higher recurrence risk after neoadjuvant immunotherapy in patients with lung cancer. Our study highlights the potential of ERO1A as a therapeutic target in cancer immunotherapy.

RESULTS

ERO1A in tumor cells deters host-protective anti-tumor T cell immunity

To determine whether ERO1A in tumor cells has an impact on host anti-tumor immunity and response to immunotherapy, three ERO1A-null (Ero1a^{KO}) cell lines, including MC-38, LLC, and B16 tumors, were introduced using CRISPR-Cas9-based genetic ablation of the *Ero1a* (Figure 1A). Deletion of *Ero1a* revealed no inhibition of cell proliferation, migration, or invasion under standard culturing conditions compared with the respective wild-type (WT) cell lines transfected with Scramble single-guide RNAs (Figures S1A–1I). Furthermore, tumor growth in C57BL/6 mice engrafted with Ero1a^{KO} LLC or B16 tumors was repressed compared with Ero1a^{WT} controls (Figures 1B and 1C). Moreover, mice were randomized to establish therapeutic tumor models by treatment with 2 mg/kg anti-PD-1 (aPD-1) every 3 days for a total of six rounds. Treatment of Ero1a^{KO} tumor-bearing mice with aPD-1 consistently resulted in augmented anti-tumor effects compared with aPD-1-treated Ero1a^{WT} controls in all therapeutic models (Figures 1B–1D and S1J). In addition, Ero1a^{KO} MC-38 tumors were markedly repressed in response to PD-1 blockade (Figures 1D–1F). Transduction of mouse ERO1A cDNA completely rescued the growth defects of Ero1a^{KO} MC-38 tumors during six rounds of aPD-1 treatment compared with those transfected with empty vector (Figure 1G), suggesting the *in vivo* synergistic anti-tumor role of ERO1A ablation. To test whether T cell activity was required for the regression of Ero1a^{KO} tumors, we implanted Ero1a^{WT} or Ero1a^{KO} MC-38 tumors into immunodeficient hosts (BALB/c nude) or depleted CD8^{+} cells in immunocompetent mice via antibody-based approaches (Figures S1K and S1L). The anti-tumor effect triggered by ERO1A ablation and aPD-1 treatment was not observed in BALB/c nude mice or anti-CD8 antibody-treated immunocompetent mice (Figures 1H and 1I), suggesting the anti-tumor role of CD8^{+} T cells in Ero1a^{KO} tumors.

To quantify intratumoral lymphocytes in therapeutic models, flow cytometry (FCM) analyses and immunofluorescence (IF) staining of MC-38 tumors were performed after two rounds of aPD-1 treatment. Compared with Ero1a^{WT} counterparts, tumors from mice bearing Ero1a^{KO} MC-38 cells exhibited a higher abundance of tumor-infiltrating CD4^{+} T, CD8^{+} T, and natural killer (NK) cells but not regulatory T cells (Tregs), macrophages, or MDSCs (Figures S2A–S2D). Furthermore, IF staining revealed that Ero1a^{KO} tumors had a higher abundance of tumor-infiltrating lymphocytes (TILs) in the tumor core region than control tumors (Figure 1J). After normalizing for tumor weight, Ero1a^{KO} tumors showed higher proportions of CD4^{+} T, CD8^{+} T, and NK cells (Figures S2E and S2F), suggesting ERO1A impedes lymphocyte infiltration. Also, PD-L1 expression was markedly increased in Ero1a^{KO} tumors with a focal expression pattern compared with WT counterparts (Figures S2G and S2H), which might be the response to IFN-γ produced by activated TILs.[16,17] Moreover, in accordance with the recovery of T cell anti-tumor immunity response to PD-1 blockade,[18] higher PD-1 expression was detected in intratumoral T cells from Ero1a^{KO} tumors than in control tumors (Figures S2I and S2J). We next tested lymphocyte activity using Luminex-based multiplexing. Interestingly, increased IFN-γ, TNF-α, IL-1β, and IL-6 secretion levels were detected in Ero1a^{KO} tumors compared with controls, while GM-CSF, IL-10, and IL-17 levels were not (Figure S2K). Taken together, ERO1A deletion enhances protective T cell immunity in TME and response to PD-1 blockade, which can be therapeutically addressed.

ERO1A reshapes the tumor microenvironment in response to PD-1 blockade

Whether ERO1A signaling affects anti-tumor immunity and clinical outcomes in patients with solid tumors remains unknown. To assess the relationship between ERO1A expression and clinical outcomes, the TCGA database was searched to acquire data regarding ERO1A expression in primary cancers for transcriptome profiling. We observed a significant association between high ERO1A expression and shorter overall survival in patients with several solid tumors, including lung cancer, breast cancer, liver cancer, and colorectal cancer (Figures S3A–S3D). ERO1A expression was also found to correlate with multiple immune checkpoints in pan-cancers (Figure S3E). In addition, ERO1A mRNA level was negatively correlated with genes that define anti-tumor immunity and immune infiltrates, including CD8^{+} T, B, and NK cells, whereas it was positively correlated with CD4^{+} T cells (Figures S4A–S4D). Thus, ERO1A signaling limits clinical outcomes and anti-tumor immunity in human cancers.

To quantitatively dissect the cellular and molecular changes in TME associated with ERO1A ablation, single-cell RNA-sequencing (scRNA-seq) analyses were performed using Ero1a^{WT} and Ero1a^{KO} tumors from MC-38 therapeutic models

CellPress
OPEN ACCESS

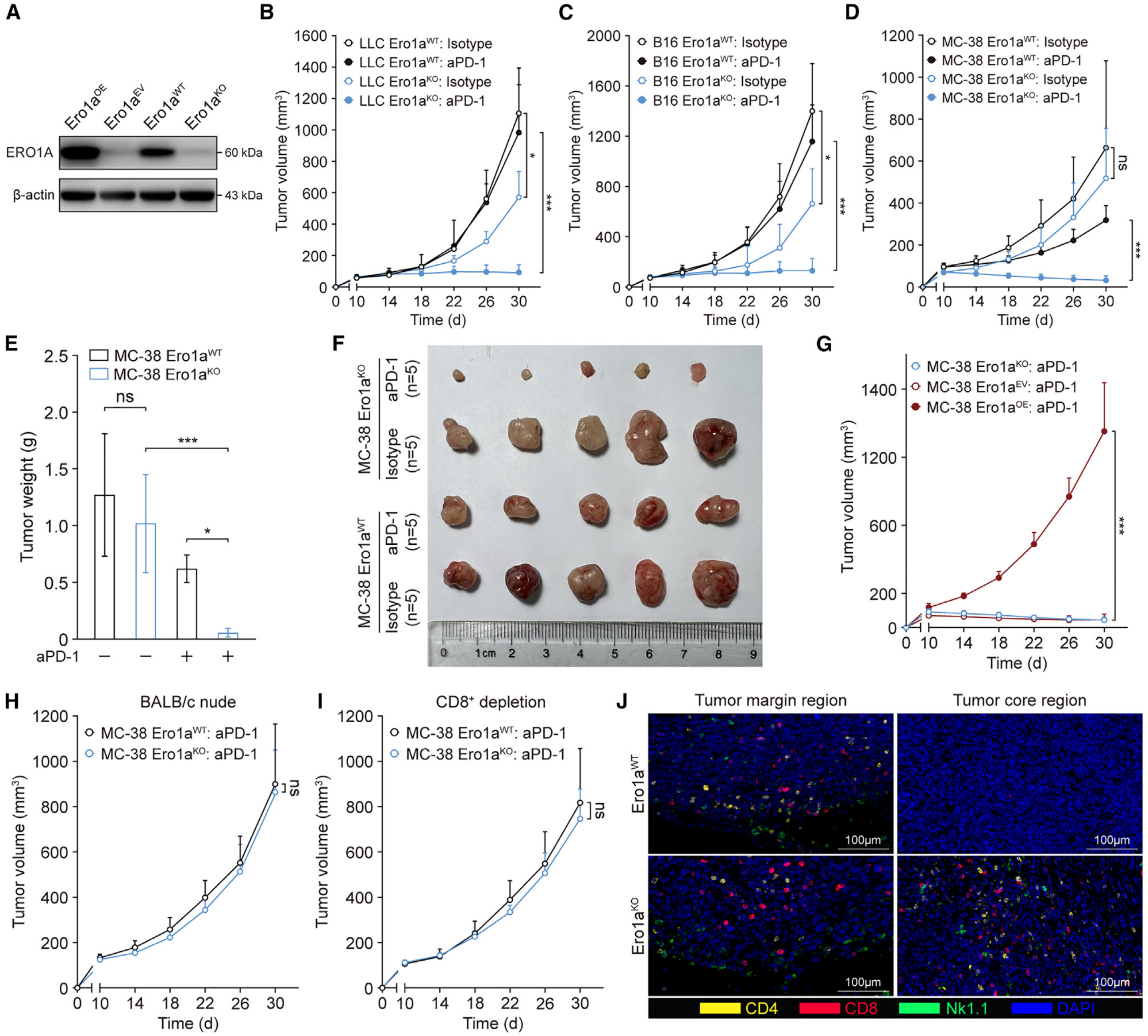

Figure 1. ERO1A attenuates the anti-tumor efficacy of aPD-1 treatment in immunocompetent hosts

(A) Western blot of ERO1A in MC-38 cells. Ero1a^{WT} cells were integrated with non-targeting CRISPR-Cas9 vector. Ero1a^{KO} cells were rescued by transduction with lentiviruses expressing empty vector (Ero1a^{EV}) or the full length of mouse ERO1A cDNA (Ero1a^{OE}). Result is a representative of three experiments.

(B–D) Tumor volume in C57BL/6 mice bearing LLC Ero1a^{WT} or LLC Ero1a^{KO} tumors (B) or B16 (C) and MC-38 counterparts (D), treated with isotype or aPD-1 blockade (n = 5 mice/group). Data presented as means ± SEMs. *$p < 0.05$, ***$p < 0.001$. ns, not significant. Two-sided Student's t test.

(E and F) Tumor weight (E) and tumor growth (F) of MC-38 Ero1a^{WT} or Ero1a^{KO} tumors in C57BL/6 hosts with or without aPD-1 treatment (n = 5 mice/group). Data presented as means ± SDs. *$p < 0.05$, ***$p < 0.001$. ns, not significant. Two-sided Student's t test.

(G) Rescue of MC-38 Ero1a^{KO} tumor growth in C57BL/6 hosts during six rounds of aPD-1 treatment (n = 5 mice/group). MC-38 Ero1a^{KO} cells were rescued by transduction with lentiviruses expressing empty vector (Ero1a^{EV}) or the full length of mouse ERO1A cDNA (Ero1a^{OE}). Data presented as means ± SEMs. ***$p < 0.001$. Two-sided Student's t test.

(H and I) Tumor volume in immunodeficient BALB/c nude mice (H) or CD8$^+$ depleted C57BL/6 mice (I) bearing MC-38 Ero1a^{WT} or Ero1a^{KO} tumors (n = 5 mice/group). Data presented as means ± SEMs. ns, not significant. Two-sided Student's t test.

(J) Multiplex immunofluorescent staining of CD4, CD8, and Nk1.1 using MC-38 Ero1a^{WT} and Ero1a^{KO} tumor samples (representative of n = 3 mice/group). Tumors were collected after two rounds of aPD-1 treatment. Scale bars, 100 μm.

after two rounds of aPD-1 treatment (Figure S5A). Each cell population was recognized using conventional marker genes (Figures 2A and S5B–S5D). A combined t-distributed stochastic neighbor embedding (t-SNE) plot distinguished eight clusters; while all the clusters from the Ero1a^{WT} and Ero1a^{KO} tumors were largely overlapped, the subtypes of lymphocytes and

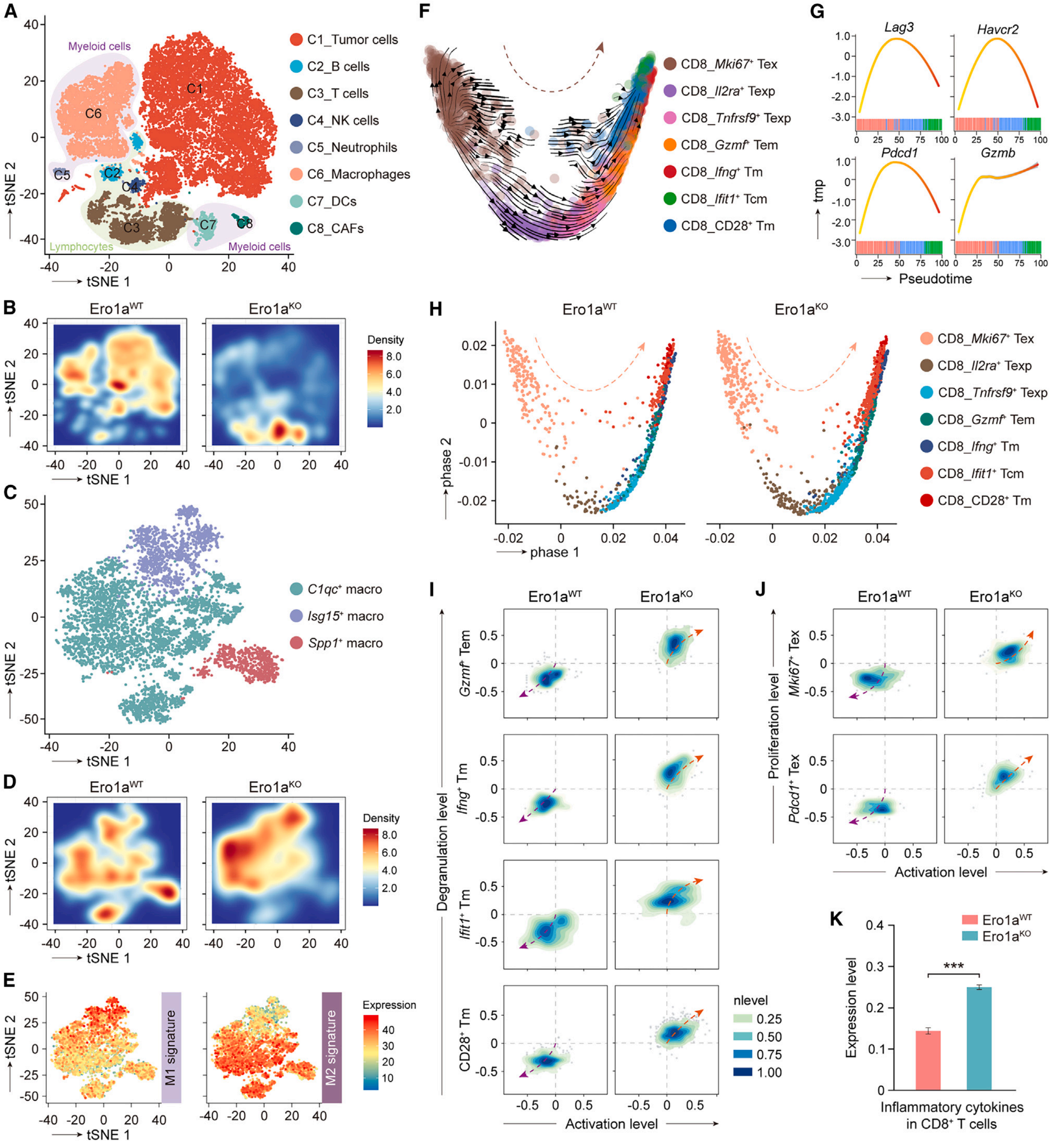

Figure 2. ERO1A induces T cell dysfunction in response to aPD-1 treatment

(A) t-distributed stochastic neighbor embedding (t-SNE) visualization of major cell clusters, colored by cell subtype. Tumors were collected after two rounds of aPD-1 treatment and processed for scRNA-seq (n = 5 mice/group). CAFs, cancer-associated fibroblasts. DCs, dendritic cells. NK, natural killer.

(B) t-SNE map of major cell types in MC-38 Ero1a^{WT} and Ero1a^{KO} tumors. Colored by cell subtype.

(C) t-SNE map indicating the macrophage clusters based on the scRNA-seq data. Colored by cell subtype.

(D) t-SNE map showing the sample origins of macrophages in MC-38 Ero1a^{WT} and Ero1a^{KO} tumors. Colored by cell subtype.

(E) t-SNE map depicting the M1 and M2 macrophage signatures based on the scRNA-seq data.

(F) Uniform manifold approximation and projection (UMAP) plot showing the RNA velocity of CD8$^+$ T cell subsets. RNA velocities were visualized on the UMAP of *Mki67*$^+$ Tex, *Il2ra*$^+$ Texp, *Tnfrsf9*$^+$ Texp, *Gzmf*$^+$ Tem, *Ifng*$^+$ Tm, *Ifit1*$^+$ Tcm, and CD28$^+$ Tm using Gaussian smoothing on a regular grid.

(legend continued on next page)

myeloid cells were dominant in the $Ero1a^{KO}$ and $Ero1a^{WT}$ tumors, respectively (Figures 2B and S5E). Moreover, after PD-1 blockade, a higher abundance of $CD8^+$ T cells was observed in $Ero1a^{KO}$ tumors compared with WT controls, and this increase was particularly prominent for $CD28^+$ Tm, *Mki67*$^+$ Tex, and *Tnfrsf9*$^+$ Texp cells (Figures S6A and S6B). In contrast, the proportion of tumor cells was markedly repressed in $Ero1a^{KO}$ tumors compared with controls (Figure 2B). We next interrogated the heterogeneous phenotypes of tumor-associated macrophages (TAMs) and explored their expression patterns. The *Spp1*$^+$ TAMs, a subtype of TAMs that provokes immunosuppression, were dominantly enriched in $Ero1a^{WT}$ tumors compared with $Ero1a^{KO}$ counterparts (Figures 2C and 2D), in accordance with the view that tumor cells were the primary driver for *SPP1*$^+$ TAMs.[19] Furthermore, we examined TAM signatures, which showed that the $Ero1a^{KO}$ and $Ero1a^{WT}$ tumors were significantly associated with M1 and M2 TAM signatures, respectively (Figures 2E and S6C), suggesting that ERO1A promotes a phenotype transition of TAMs. Thus, these results suggest the immunosuppressive role of ERO1A in TME remodeling in response to immunotherapy.

ERO1A in tumor cells instigates T cell dysfunction in response to immunotherapy

Given the anti-tumor role of $CD8^+$ T cells in $Ero1a^{KO}$ tumors, we next examined the proliferation and cytotoxic function of $CD8^+$ T cells in therapeutic models. A revival trajectory of $CD8^+$ T cells by RNA velocity analysis was calculated from the *Mki67*$^+$ Tex cells, followed by the *Il2ra*$^+$ Texp and *Tnfrsf9*$^+$ Texp cells, to the *Gzmf*$^+$ Tem, *Ifng*$^+$ Tm, *Ifit1*$^+$ Tcm, and $CD28^+$ Tm cells (Figure 2F). *Tnfrsf9*$^+$ Texp cells were located at the center of the trajectory, suggesting that a fraction of Texp cells responded to aPD-1 treatment. Moreover, marker gene expression for exhausted $CD8^+$ T cells, including *Lag3*, *Havcr2*, and *Pdcd1*, was increased during the resting stage and reduced during the activated stage (Figure 2G). Thus, aPD-1 blockade *in vivo* rescued the dysfunction of cytotoxic effector $CD8^+$ T cells. The dynamically expressed genes along the trajectory were identified and grouped into three modules (Figures S6D and S6E). Interestingly, it was revealed that *Mki67*$^+$ T cells in both $Ero1a^{KO}$ and $Ero1a^{WT}$ tumors could be reinvigorated by PD-1 blockade (Figure 2H). To quantitatively compare the anti-tumor cytotoxicity of $CD8^+$ T cells in $Ero1a^{KO}$ and $Ero1a^{WT}$ tumors, we next performed dimensionality reduction and projected each single $CD8^+$ T cell by scoring activation, proliferation, and degranulation levels. The contour plot showed that the four effective $CD8^+$ T cell clusters ($CD28^+$, *Ifit1*$^+$, *Ifng*$^+$, and *Gzmf*$^+$ T cells) exhibited significantly higher anti-tumor capacity in $Ero1a^{KO}$ tumors compared with WT counterparts (Figure 2I). Similarly, the *Pdcd1*$^+$ and *Mki67*$^+$ clusters of $CD8^+$ T cells in $Ero1a^{KO}$ tumors had significantly higher proliferative capacity than in WT tumors (Figure 2J). Consistently, a higher signature of inflammatory cytokines was observed in $CD8^+$ T cells from $Ero1a^{KO}$ tumors compared with counterparts from controls (Figure 2K). These results suggest that tumor ERO1A instigates $CD8^+$ T cell dysfunction during PD-1 blockade.

Ablation of tumor ERO1A promotes anti-tumor immunity via ICD

To understand the synergistic anti-tumor effects induced by the deletion of ERO1A in tumors, we compared the transcriptomes of identified tumor clusters from $Ero1a^{WT}$ and $Ero1a^{KO}$ tumors based on scRNA-seq analyses (Figure 3A). The $Ero1a^{KO}$ tumor cells showed upregulation of pathways such as "negative regulation of response to ER stress" and "intrinsic apoptotic signaling pathways in response to ER stress," suggesting a higher ER stress burden and increased cell deaths compared with WT counterparts (Figure S7A). Interestingly, RNA velocity analysis revealed transitions from the T2, T3, and T4 to the T1 subtype, characterized as hypoxia tumor cells (Figure 3B). Because oxygen deprivation yields ER stress in tumor cells, we next performed gene set enrichment analysis (GSEA) within T1 tumor cells. Similarly, GSEA showed upregulation of "hallmark of apoptosis," "response to ER stress," and "intrinsic apoptotic signaling pathways in response to ER stress" in T1 tumor clusters from $Ero1a^{KO}$ tumors compared with WT counterparts (Figure 3C). Moreover, genes associated with MHC-I and MHC-II protein binding were significantly enriched in the T1 population from $Ero1a^{KO}$ tumors compared with counterparts from controls (Figures S7B and S7C), consistent with the results that ERO1A mediates immunosuppression.

The ability to tolerate persistent, non-lethal ER stress enhances tumor cell survival and mediates immunosuppression. Since $Ero1a^{KO}$ tumor cells were associated with apoptotic signaling pathways in response to ER stress, we interrogated the role of ERO1A in the cell survival of tumors undergoing ER stress. We introduced three *in vitro* models of ER stress, including hypoxia-induced, metabolic-induced, and tunicamycin-induced ER-stress conditions. $Ero1a^{KO}$ tumor cells showed increased susceptibility to ER stress compared with WT tumors, suggested by the lower cell viability of tumor cells under ER-stressed conditions (Figure 3D). Increased lactate dehydrogenase (LDH) release was also observed in $Ero1a^{KO}$ tumors compared with WT controls (Figure 3E), indicating a terminal ER stress response that led to cell death. Of note, ICD-associated immunogenicity can be evoked by lethal ER stress response and subsequently enhances host anti-tumor immunity.[20,21] To determine whether $Ero1a^{KO}$ tumors underwent ER stress-triggered ICD *in vivo*, we first explored the expression of damage-associated molecular pattern (DAMP)-related genes, which were identified as mediators of the immunogenic characteristics of ICD,[22] based on the scRNA-seq data in MC-38

(G and H) Diffusion map of $CD8^+$ T cell clusters shows a resting-to-activated trajectory. The pseudotime expression changes in *Lag3*, *Pdcd1*, *Havcr2*, and *Gzmb* in $CD8^+$ T cells (G). Pseudotime trajectory of $CD8^+$ T cell subsets in MC-38 $Ero1a^{WT}$ and $Ero1a^{KO}$ tumors (H). Colored by cell subtype.

(I and J) Projection of effective $CD8^+$ T cells (I) and proliferative $CD8^+$ T cells (J) based on cell activation, degranulation, and proliferation levels in MC-38 $Ero1a^{WT}$ and $Ero1a^{KO}$ tumors.

(K) Bar plot showing the inflammatory cytokines of $CD8^+$ T cells in MC-38 $Ero1a^{WT}$ and $Ero1a^{KO}$ tumors, assessed by scRNA-seq. Data presented as means ± SDs. ***$p < 0.001$. Two-sided Student's t test.

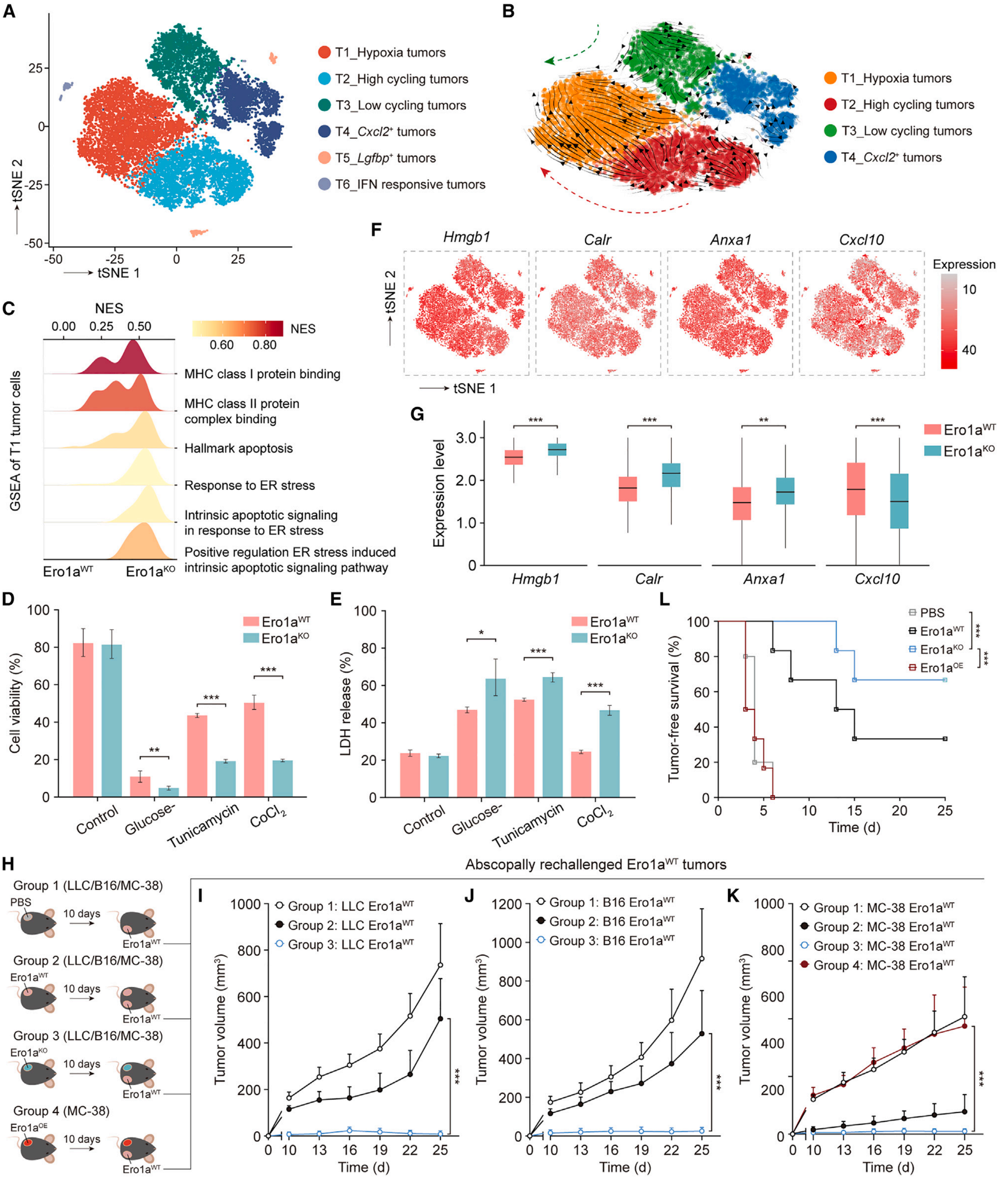

Figure 3. Ablation of ERO1A promotes anti-tumor immunity via ICD

(A) t-SNE map showing the tumor cell clusters based on the scRNA-seq data. Colored by cell subtype.

(B) UMAP plot showing the RNA velocity of tumor cell subsets. RNA velocities were visualized on the UMAP of the T1-hypoxia, T2-high cycling, T3-low cycling, and T4-*Cxcl2*$^{+}$ tumor cell subtypes using Gaussian smoothing on a regular grid.

(legend continued on next page)

therapeutic models. We found that several DAMP-related genes, including *Hmgb1*, *Calr*, and *Anxa1*, were significantly overexpressed in Ero1a^{KO} tumor cells compared with WT controls, suggesting the intracellular danger signaling pathways that govern ICD in Ero1a^{KO} tumors (Figures 3F and 3G).

To confirm the activation of ICD-associated immunogenicity in Ero1a^{KO} tumor-bearing mice, we tested the systemic anti-tumor protection from secondary-tumor challenge at distant sites. Mice were subcutaneously implanted with PBS, Ero1a^{WT}, or Ero1a^{KO} tumor cells on the left flank and were further challenged with Ero1a^{WT} control cells on the right flank 10 days later (Figure 3H). Initial transplantation with Ero1a^{WT} LLC or B16 tumors did not confer tumor growth from the rechallenge with WT tumor cells (group 2, Figures 3I and 3J). In contrast, injection with Ero1a^{KO} tumors markedly repressed the growth of contralaterally injected Ero1a^{WT} tumors in both LLC and B16 models (group 3, Figures 3I and 3J), suggesting that ERO1A ablation triggered ICD *in vivo*. Of the eight mice pre-injected with WT B16 or LLC cells, all developed palpable tumors on the contralateral side, while only six of eight with Ero1a^{KO} B16 and five of eight with LLC cells developed tumors (Figure S7D). This abscopal anti-tumor effect was not significantly observed in Ero1a^{KO} MC-38 tumors compared with WT controls (Figures 3K and S7E). However, compared with mice injected with Ero1a^{OE} MC-38 tumors (group 4), those previously injected with Ero1a^{KO} MC-38 tumors showed substantially reduced tumor growth upon rechallenge with Ero1a^{WT} MC-38 tumors (Figures 3K and S7E), leading to the improved tumor-free survival (Figure 3L). Moreover, we examined the acquisition of memory anti-tumor responses against Ero1a^{KO} tumors. Immunocompetent mice initially injected with Ero1a^{KO} MC-38 tumors acquired resistance to WT MC-38 tumors, in contrast to the PBS-pre-treated mice (Figure S7F). Furthermore, this protective effect was not observed against unrelated LLC or B16 tumors (Figures S7G and S7H), indicating the protective tumor-specific immune memory induction. Collectively, these results suggest that ERO1A deletion triggers lethal ER stress responses in tumors and promotes host anti-tumor immunity via ICD.

ERO1A ablation in tumors leads to defects in ER stress response

Eukaryotic cells under ER stress will activate the UPR to restore protein homeostasis, which involves three main pathways: IRE1α, PERK, and ATF6α.[7] To directly test whether ERO1A contributes to the UPR, we examined the expression levels of IRE1α, PERK, and ATF6α proteins in tunicamycin-treated MC-38 cells. The PERK pathway was more activated in Ero1a^{KO} MC-38 tumor cells compared with WT counterparts, as shown by the phosphorylation of PERK and EIF2α (Figure 4A). In contrast, the IRE1α pathway was activated in Ero1a^{WT} cells but not in Ero1a^{KO} cells, as demonstrated by the phosphorylation of IRE1α and the spliced form of downstream Xbox binding protein-1 (XBP1), which promotes protein folding in the ER (Figure 4B). In addition, impaired activation of the IRE1α pathway in Ero1a^{KO} cells was confirmed by scRNA-seq based on MC-38 therapeutic models showing decreased expression levels of XBP1 target genes relative to WT controls (Figure 4C). Consistently, the scRNA-seq analysis revealed that the expression levels of *Perk*, *Eif2a*, *Chop*, and *Casp12* were significantly upregulated in the T1 tumor cluster from Ero1a^{KO} tumors compared with WT counterparts (Figure 4D), suggesting they were more susceptible to death. Thus, ERO1A-disrupted tumor cells may not be able to resolve the ER stress due to impaired activation of the IRE1α pathway, resulting in an imbalance between the IRE1α activity and PERK activation that governs cell fate.[23–25] To determine whether the IRE1α pathway contributes to the *in vivo* therapeutic phenotype, we treated ER-stressed Ero1a^{WT} MC-38 cells with Kira6, an IRE1α-specific inhibitor. Treatment with Kira6 markedly decreased the cell viability compared with vehicle treatment (Figures 4E and S7I). We also assessed the effect of Kira6 on the proliferation of Ero1a^{WT} MC-38 cells by incubation with EdU (thymidine analog 5-ethynyl-2'-deoxyuridine) overnight in the presence or absence of Kira6. Inhibition of IRE1α with Kira6 significantly decreased the proportion of cells incorporating EdU in Ero1a^{WT} cells pretreated with tunicamycin (Figures 4F and 4G). We next tested the anti-tumor effects of Kira6 in therapeutic models. Consistently, administration of Kira6 to tumor-bearing mice significantly affected the growth of MC-38 Ero1a^{WT} tumors compared with those treated with vehicle (Figures 4H and, S7J).

ERO1A in tumor cells promotes transmissible ER stress in the TME

ER stress induces tumor cells to release unknown soluble factors that activate the UPR and pro-inflammatory cytokines in responder immune infiltrates, thereby remodeling an

(C) GSEA of T1-hypoxia tumor cell subsets showing higher enrichment of ER stress response and apoptotic signaling pathway in Ero1a^{KO} tumors, compared with those in Ero1a^{WT} tumors.

(D and E) Comparison of cell viability (D) and LDH release-based (E) cell death in MC-38 Ero1a^{WT} and Ero1a^{KO} tumors treated with glucose-deprived medium, 0.3 μg/mL tunicamycin, or 100 μmol $CoCl_2$. Tumor cells were harvested after 24-h incubation under ER-stressed conditions. Data presented as means ± SDs from 10 technical replicates. *$p < 0.05$, **$p < 0.01$, ***$p < 0.001$. Two-sided Student's t test.

(F) t-SNE plots showing expression of damage-associated molecular pattern (DAMP)-related genes as identified by the scRNA-seq analysis of therapeutic models.

(G) Boxplots showing the relative expression levels of DAMP-related genes in MC-38 Ero1a^{WT} and Ero1a^{KO} tumors, measured by scRNA-seq. Boxplots show the interquartile range (IQR) divided by the median. **$p < 0.01$, ***$p < 0.001$. Wilcoxon signed-rank test.

(H) Diagram of tumor rechallenge experiment. The first challenge was with PBS (group 1), Ero1a^{WT} (group 2), Ero1a^{KO} (group 3), or MC-38 Ero1a^{OE} (group 4) tumor cells on the left flank of C57BL/6 mice, and the rechallenge was performed after 10 days with Ero1a^{WT} tumor cells on the right flank.

(I–K) Right-flank tumor growth from rechallenged mice bearing LLC Ero1a^{WT} (I), B16 Ero1a^{WT} (J), or MC-38 Ero1a^{WT} (K) tumors. Data presented as means ± SEMs. Representative of n = 8 mice in LLC and B16 models, n = 5/6 mice in MC-38 models. ***$p < 0.001$. Two-sided Student's t test.

(L) Tumor-free survival for MC-38 Ero1a^{WT} rechallenged mice. Mice were initially transplanted with PBS (n = 5 mice), Ero1a^{WT} (n = 5 mice), Ero1a^{KO} (n = 6 mice), or Ero1a^{OE} MC-38 cells (n = 6 mice). Kaplan-Meier curves of tumor-free survival for mice after secondary tumor rechallenge. ***$p < 0.001$. Log rank test.

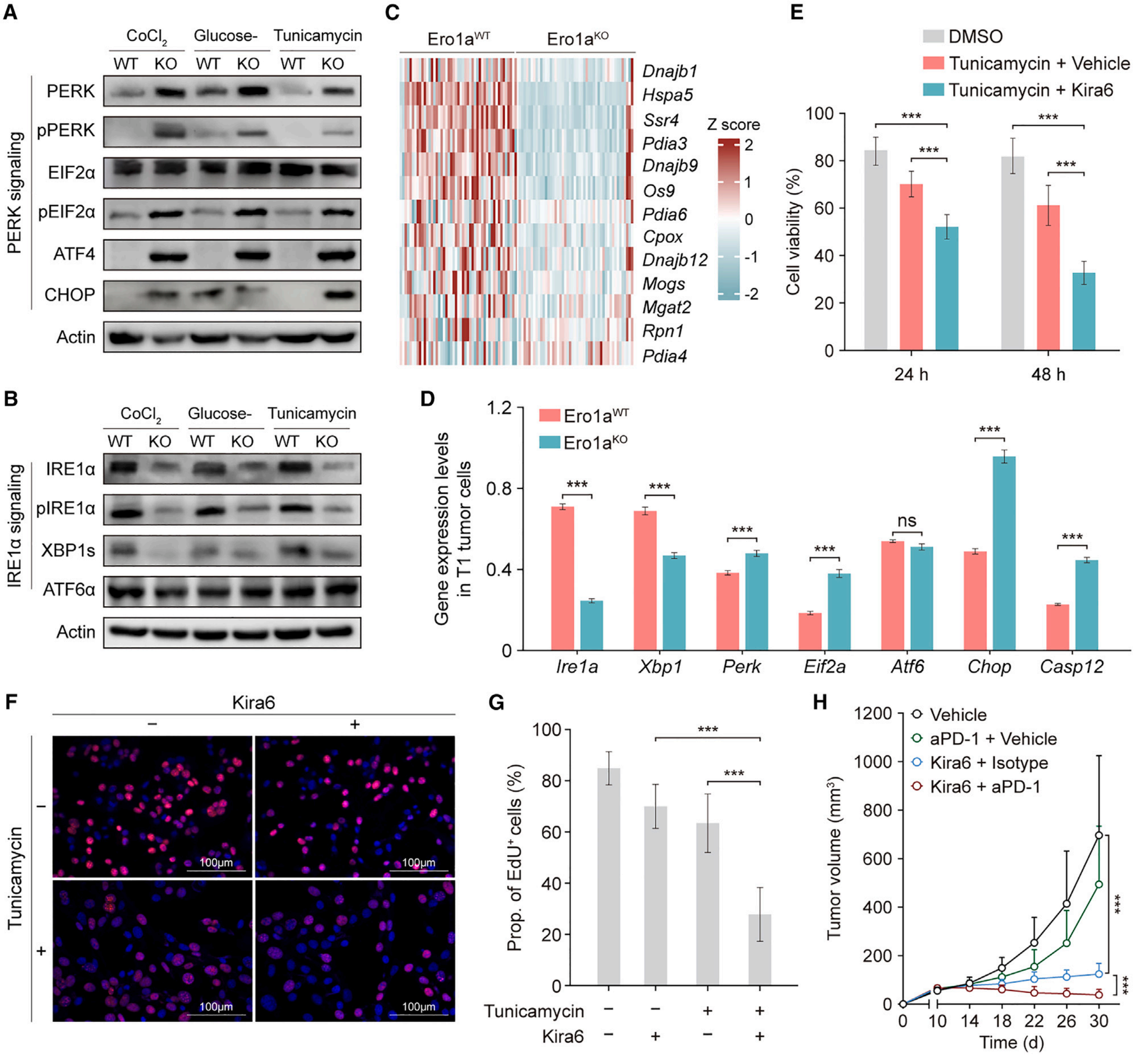

Figure 4. ERO1A ablation in tumors leads to defects in ER stress response

(A and B) Western blots of PERK, pPERK, EIF2α, pEIF2α, ATF4, and CHOP (A) and IRE1α, pIRE1α, XBP1s, and ATF6α (B) in MC-38 Ero1a^{WT} and Ero1a^{KO} tumor cells treated with glucose-deprived medium, 0.3 μg/mL tunicamycin, or 100 μmol $CoCl_2$. Tumor cells were harvested after 24 h of ER-stress induction (n = 3 independent repeats).

(C) Heatmap representing the expression levels of XBP1 target genes in Ero1a^{KO} tumors compared with those in Ero1a^{WT} tumors, based on the scRNA-seq data in MC-38 therapeutic models.

(D) Bar plot showing the gene expression levels of *Ire1a*, *Xbp1*, *Perk*, *Eif2a*, *Atf6*, *Chop*, and *Casp12* in T1-hypoxia tumor cell cluster as analyzed by scRNA-seq data in MC-38 therapeutic models. Data presented as means ± SDs. ***p < 0.001. ns, not significant. Two-sided Student's t test.

(E) Comparison of cell viability in ER-stressed MC-38 Ero1a^{WT} cells treated with vehicle or Kira6. Tumor cells were treated with 0.3 μg/mL tunicamycin plus vehicle (Veh) or 0.3 μg/mL tunicamycin plus Kira6 for 24 or 48 h. Data presented as means ± SDs from eight technical replicates. ***p < 0.001. Two-sided Student's t test.

(F and G) EdU staining (F) and quantification (G) of MC-38 Ero1a^{WT} tumor cells treated with tunicamycin or Kira6 (representative of n = 3 mice). Data presented as means ± SDs from 12 randomly selected fields. ***p < 0.001. Two-sided Student's t test. Scale bars, 100 μm.

(H) Tumor volume in C57BL/6 mice bearing MC-38 Ero1a^{WT} tumors treated with vehicle, aPD-1 plus vehicle, Kira6 plus isotype, or Kira6 plus aPD-1 blockade (n = 6 mice/group). Data presented as means ± SEMs. ***p < 0.001. Two-sided Student's t test.

immunosuppressive TME.[26,27] We next interrogated whether infiltrating T cells were compelled to initiate the UPR through this ‘‘transmissible ER stress’’ process, thus affecting the cytotoxic functions. Based on the scRNA-seq data of MC-38 therapeutic models, it was noticed that UPR-related genes were highly expressed in T cell populations (Figure S8A), suggesting the ER-stressed condition in lymphocytes. Interestingly, we noticed that ‘‘response to ER stress’’ and ‘‘intrinsic apoptotic signaling pathways in response to ER stress’’ pathways were significantly enriched in T cells of Ero1a^{WT} samples compared with those of Ero1a^{KO} samples (Figure S8B). Also, GSEA revealed that gene sets related to ‘‘programmed cell death’’ and ‘‘response to ER stress’’ were positively enriched in CD8^{+} T cells from Ero1a^{WT} tumors (Figures 5A and S8C), suggesting more UPR-related cell death compared with Ero1a^{KO} tumors. To assess the ER-stressed condition of CD8^{+} T cells, we performed qRT-PCR analyses of UPR-related genes in MC-38 therapeutic models. Reduced mRNA levels of UPR-related genes were detected in CD8^{+} T cells isolated from Ero1a^{KO} MC-38 tumors treated with aPD-1, compared with Ero1a^{WT} counterparts (Figure 5B). CD8^{+} T cells in Ero1a^{KO} tumors exhibited subtracted susceptibility to ER-stress-induced cell death compared with controls, as indicated by the lower expression levels of *Chop* and *Casp12* (Figure 5B). In addition, co-culturing of CD8^{+} T cells with tunicamycin pre-treated Ero1a^{KO} tumor cells showed significant increase in the release of IFN-γ, TNF-α, and GzmB compared with controls (Figure 5C), indicating that tumor ERO1A induces T cell dysfunction. The nature of cell death was also confirmed by FCM analyses of CFDA-SE and propidium iodide (PI) staining (Figure S8D). An increased proportion of CFDA-SE and PI dual-positive cells was noted in ERO1A^{KO} tumors pre-treated with tunicamycin after exposure to activated CD8^{+} T cells (Figures 5D and 5E), suggesting enhanced cytotoxic activity of CD8^{+} T cells when co-cultured with ERO1A^{KO} tumors undergoing ER stress. Based on the scRNA-seq data of MC-38 therapeutic models, a pseudotime trajectory was calculated during the aPD-1 treatment, which revealed significantly reduced apoptosis gene signature in MC-38 ERO1A^{KO} tumors compared with ERO1A^{WT} tumors (Figure 5F), suggesting the susceptibility of CD8^{+} T cells to death in Ero1a^{WT} tumors. To demonstrate the potential crosstalk between ER-stressed tumor cells and CD8^{+} T cells, we performed a cell-to-cell interaction analysis. The CellChat algorithm identified SPP1 as the specific signaling pathway involved in the intercellular crosstalk between tumor cells and CD8^{+} T cells associated with Ero1a^{WT} tumors (Figure 5G), which was also confirmed using the iTALK algorithm (Figure S8E).

ERO1A as a biomarker in patients treated with immunotherapy

We next wondered whether ERO1A-induced TME remodeling exists in patients treated with immunotherapy. Thirty-seven tumor samples from patients with non-small cell lung cancer (NSCLC) who received neoadjuvant aPD-1 treatment were collected from the NCC cohort. Immunohistochemistry (IHC) staining distinguished 15 patients with low ERO1A expression (IHC −/+) and 22 patients with high ERO1A expression (IHC ++/+++, Figures 6A and S9). The baseline patient demographic and disease characteristics were dichotomized by ERO1A expression in Table 1. No statistically significant differences were observed related to age, gender, smoking status, histology, tumor stage, tertiary lymphoid structures (TLSs), or treatment regimen (Figures 6B and 6C). Of the 11 patients with lung adenocarcinoma (LUAD), 6 (54.6%) had at least one actionable mutation, of which 3 (27.3%) were with EGFR mutations, 3 (27.3%) showed KRAS mutations, and none were with BRAF mutations. However, Spearman’s correlation analysis indicated no significant relationship between mutation status and ERO1A expression ($R = 0.149$, $p = 0.662$) or clinical response ($R = 0.128$, $p = 0.708$).

Radiographic results demonstrated a statistically significant difference in terms of the clinical response between the two groups (Figure 6D). Of the 15 ERO1A^{low} patients, 13 (87%) had a partial response (PR), and 2 (13%) had stable disease (SD). In contrast, 4 (18%) of 22 patients in the ERO1A^{high} group achieved PR, 14 (64%) had SD, and 4 (18%) had disease progression (PD). Although the pathological response rate $\geq$90% was higher in the ERO1A^{low} group (7 of 15) compared with the ERO1A^{high} group (6 of 22), the difference between the pathological response rate and ERO1A expression was not statistically significant (Figure 6E). Radiographic results are provided in Figures 6F–6H and S10. Overall, 93.3% (14 of 15) of evaluable patients in the ERO1A^{low} group experienced a reduction from baseline in target lesion size, compared with 63.6% (14 of 22) in the ERO1A^{high} group (Figure 6I). There was also a statistically significant decrease in tumor burden in the ERO1A^{low} group between baseline and after neoadjuvant immunotherapy, which was not observed in the ERO1A^{high} group (Figure 6J). Furthermore, relapse-free survival (RFS) was significantly longer in the ERO1A^{low} group than in the ERO1A^{high} group (hazard ratio, 0.26; 95% CI, 0.07–0.91; $p = 0.034$, Figure 6K).

To characterize TME features in the two groups along the treatment, we quantified the presence of CD4^{+} T cells, CD8^{+} T cells, and CD68^{+} macrophages in patients’ tumor samples using multiplex IHC. The density of infiltrating CD4^{+} T cells and CD8^{+} T cells was significantly higher in the ERO1A^{low} group, while CD68^{+} macrophages were significantly more abundant in the ERO1A^{high} group (Figures 6L, S11A, and S11B). In addition, the ERO1A^{low} group was associated with lower IRE1α expression levels compared with their ERO1A^{high} counterparts. To further investigate whether ERO1A expression was associated with better clinical outcomes in patients treated with immunotherapy, we collected transcriptome profiling combined with corresponding clinical data of ICI-treated patients with NSCLC (GEO: GSE190265) and melanoma (ENA: PRJEB23709). Using progression-free survival (PFS) as the endpoint, it was observed that low ERO1A mRNA expression was associated with better PFS in both cohorts (Figures S11C and S11D). Taken together, these results suggest that ERO1A expression is associated with the efficacy of ICI treatment in patients with NSCLC and melanoma.

DISCUSSION

The concept of effector immune cell deployment (EICD) has been proposed for tumor immune phenotyping and includes

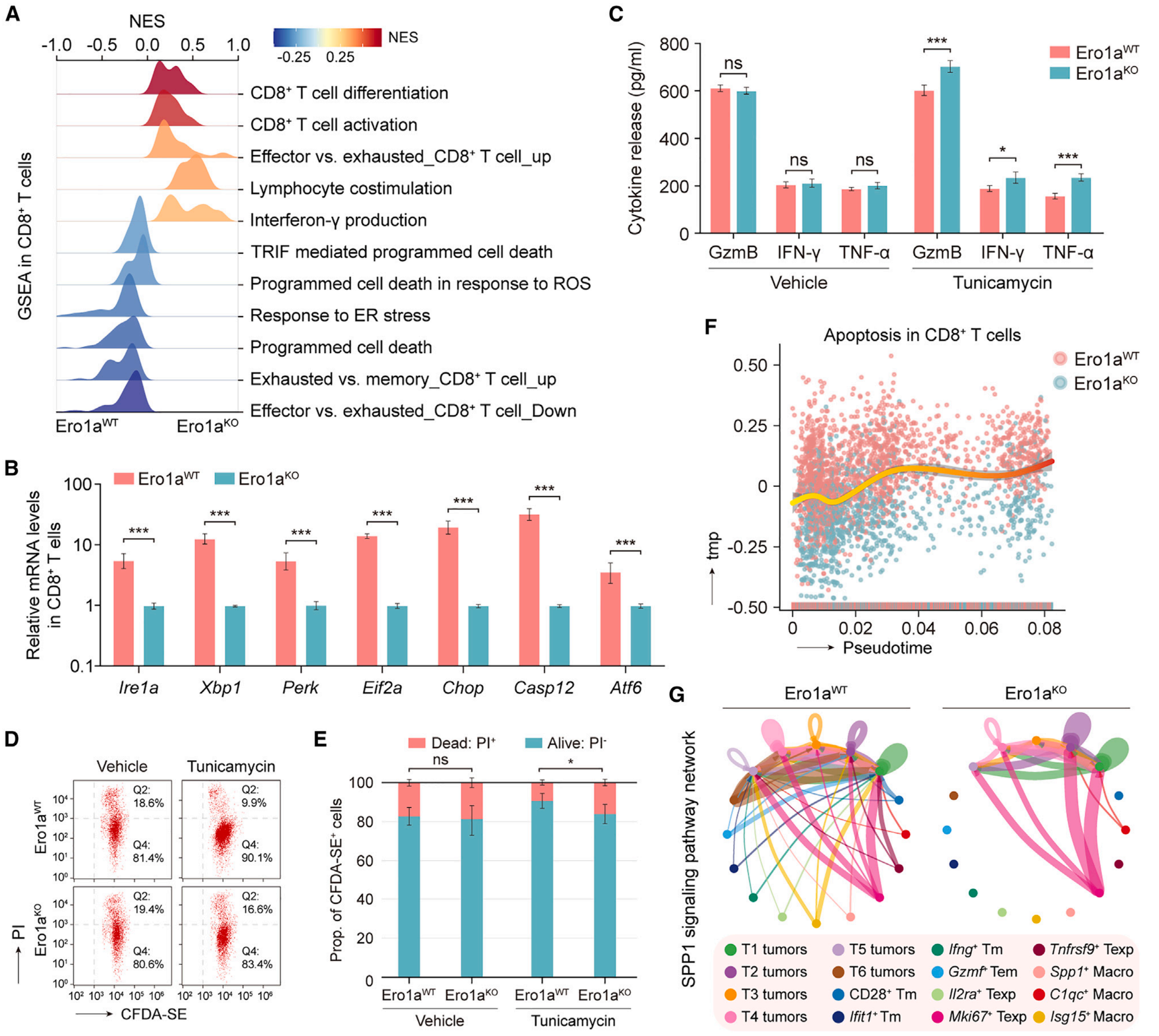

Figure 5. ERO1A in tumor cells promotes transmissible ER stress in TME

(A) GSEA showing higher enrichment of ER stress response and cell death in $CD8^+$ T cells of $Ero1a^{WT}$ tumors than in $Ero1a^{KO}$ tumors, based on the scRNA-seq data in MC-38 therapeutic models.

(B) Bar plot showing the relative mRNA expression levels of *Ire1a*, *Xbp1*, *Perk*, *Eif2a*, *Chop*, *Casp12*, and *Atf6* in $CD8^+$ T cells by qRT-PCR. $CD8^+$ T cells were isolated from MC-38 $Ero1a^{WT}$ or $Ero1a^{KO}$ therapeutic models. Data presented as means ± SDs from six technical replicates. ***$p < 0.001$. Two-sided Student's t test.

(C) Bar plot indicating the cytokine release of granzyme B, IFN-γ, and TNF-α from T cells when co-cultured with MC-38 $Ero1a^{WT}$ or $Ero1a^{KO}$ tumor cells under ER stress. The ER-stress condition was induced by treatment with 0.3 μg/mL tunicamycin. Data presented as means ± SDs from four technical replicates. *$p < 0.05$, ***$p < 0.001$. ns, not significant. Two-sided Student's t test.

(D and E) Flow cytometry of propidium iodide (PI) and CFDA-SE-stained MC-38 cells (D). Quantification of dead (CFDA-SE^+ and PI^+) or alive tumor cells (CFDA-SE^+ and PI^-) by T cell cytotoxic functional assay (E). MC-38 $Ero1a^{WT}$ or $Ero1a^{KO}$ cells with or without 0.3 μg/mL tunicamycin treatment were co-cultured with activated $CD8^+$ T cells for 24 h. Data presented as means ± SEM from three technical replicates. *$p < 0.05$. ns, not significant. Chi-squared test.

(F) Diffusion map of $CD8^+$ T cell clusters shows an apoptotic trajectory. The pseudotime expression changes in apoptotic signatures in $CD8^+$ T cells of MC-38 $Ero1a^{WT}$ and $Ero1a^{KO}$ tumors, based on the scRNA-seq data in MC-38 therapeutic models.

(G) Cellular crosstalk within SPP1 signaling pathway in MC-38 $Ero1a^{WT}$ (left) or $Ero1a^{KO}$ (right) tumors using CellChat algorithm, measured by scRNA-seq. Colored by cell subtype.

CellPress
OPEN ACCESS

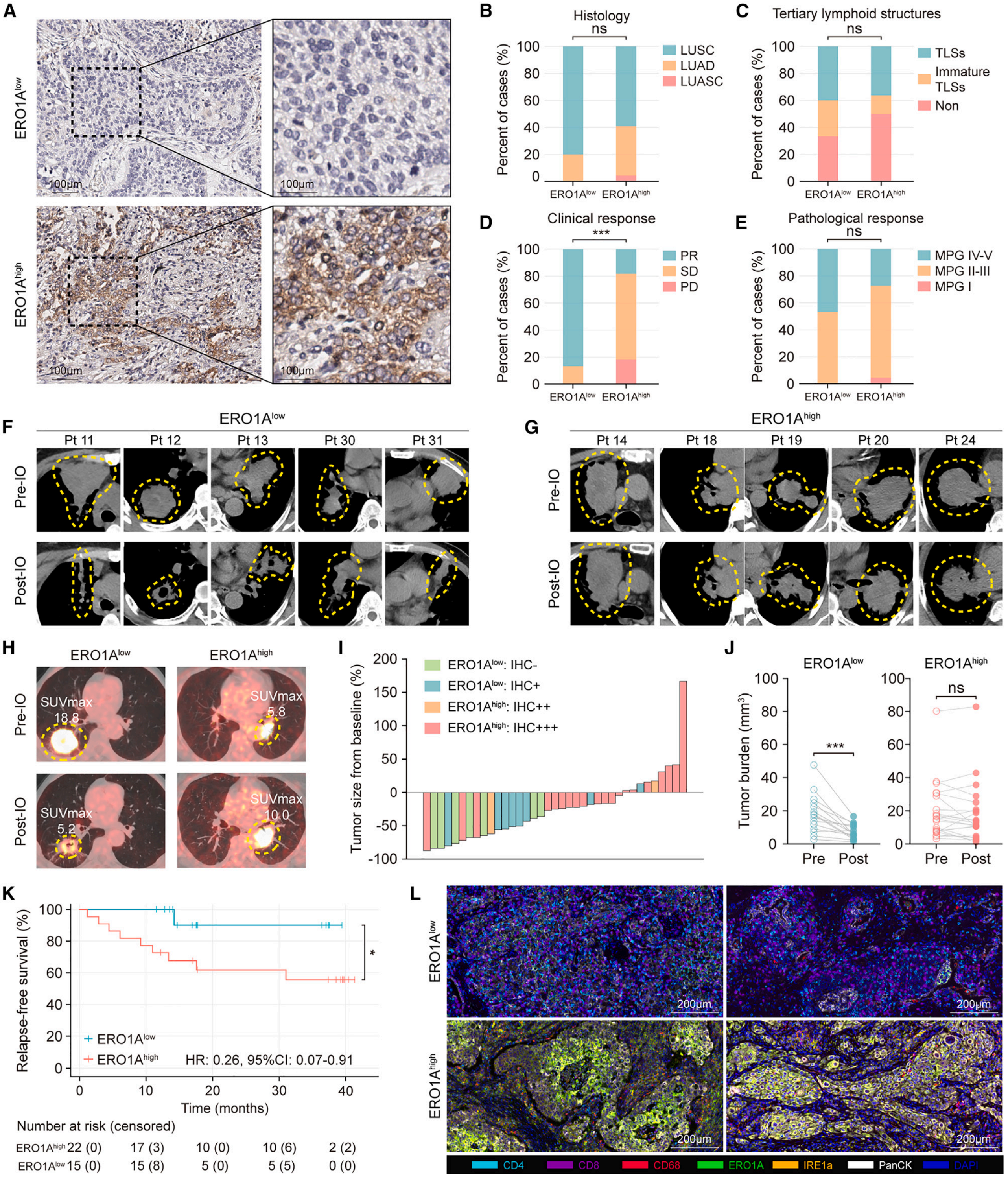

Figure 6. ERO1A as a biomarker in patients treated with immunotherapy

(A) Immunohistochemistry (IHC) staining of ERO1A in NSCLC tumors that received neoadjuvant immunotherapy. IHC plots represent ERO1A^{low} tumors (upper) and ERO1A^{high} tumors (bottom). Scale bars, 100 μm.

(B–E) Comparisons of baseline demographic and disease characteristics between ERO1A^{low} (n = 15 patients) and ERO1A^{high} groups (n = 22 patients), including histology (B), tertiary lymphoid structures (C), clinical response (D), and pathological response (E). PR, partial response; SD, stable disease; PD, progression of

(legend continued on next page)

the infiltration, activity, and fate of anti-tumor effector immunocytes.[2] Here, we showed that ERO1A reshapes the TME to drive persistent immunosuppression maintained by the ER stress response, thus affecting immune context and response to PD-1 blockade. Notably, tumors with high ERO1A expression are characterized as immune-suppressive phenotypes, and patients with these tumors have poor clinical outcomes. Our study uncovers a TME-based mechanism of ERO1A-induced immunosuppression and resistance to PD-1 blockade. These finding shed light on our understanding of the EICD hypothesis regarding TME plasticity and heterogeneity.[28]

There is growing evidence that intrinsic ER stress responses in tumors promote malignant progression by altering immune cell functions, which co-exist in the TME.[29–32] The ER stress responses in the tumor cells have been proposed to alter NK cell-mediated recognition of tumors.[33] The IRE1α-XBP1 pathway suppresses expression of NKG2D, thus attenuating NK cell-driven anti-tumor toxicity in melanoma cell lines. Furthermore, ER stress responses in tumor cells can promote the recruitment and immunosuppressive activity of MDSCs.[34] We show that depletion of ERO1A in tumor cells may not be able to resolve ER stress due to impaired activation of the IRE1α pathway, resulting in an imbalance between IRE1α activity and PERK activation, which governs cell fate. PERK activation has been reported to attenuate IRE1α phosphorylation and RNase activity through the phosphatase RNA polymerase II-associated protein 2.[25] Activation of the IRE1α pathway has been shown to suppress mitochondrial activity and IFN-γ production in T cells[32] and drive immunosuppressive reprogramming of intratumoral myeloid cells by promoting cholesterol production.[35]

Immunotherapies and checkpoint inhibitors have revolutionized the field of cancer treatment yet are effective in only a fraction of patients with solid tumors.[36,37] We show that disruption of ERO1A may trigger a lethal ER stress response in tumor cells and promote host anti-tumor immunity via ICD. Our study will inform the identification of responders to immunotherapy and development of therapeutic agents that overcome the immunosuppressed status. For example, inhibition of ERO1A/IRE1α in tumors might have a synergetic anti-tumor effect with immune checkpoint blockade by turning the tumor immunogenic and removing immune-suppressive signals, thereby restoring the anti-tumor capacity of the T cells in tumor hosts. Further study is warranted to test the safety and efficacy of anti-ERO1A therapy in various cancers.

Limitations of the study

Our study provides a method of converting non-responsive cold tumors into hot ones. Whether this or a similar tactic could be applied to other solid tumors, especially in the case of immunophenotyping, is an interesting question for future studies. The therapeutic models employed in this study utilized subcutaneous tumor models for *in vivo* experiments. However, considering the impact of tumor site on the heterogeneity of the TME, orthotopic tumor models would be more optimal for recapitulating TME features. Furthermore, we identified SPP1 as the specific signaling pathway involved in the intercellular crosstalk between tumor cells and $CD8^+$ T cells associated with $Ero1a^{WT}$ tumors; however, the rigid cell-to-cell interactions within the ERO1A-associated TME remodeling require more functional studies.

STAR★METHODS

Detailed methods are provided in the online version of this paper and include the following:

- KEY RESOURCES TABLE
- RESOURCE AVAILABILITY
    - Lead contact
    - Materials availability
    - Data and code availability
- EXPERIMENTAL MODEL AND STUDY PARTICPANT DETAILS
    - Human lung cancer tissue imaging and specimens
    - Mouse models and treatment
    - Cell culture
- METHOD DETAILS
    - IHC and mIHC staining
    - Construction of ERO1A knockout and expression plasmids
    - *In vitro* treatment and cell-death assays
    - Cell proliferation and migration assays
    - T cell isolation and cytotoxic analysis
    - Western blotting
    - Antibody staining and flow cytometry
    - Quantitative RT-PCR
    - Enzyme-linked immunosorbent assay
    - Luminex-based multiplexing of cytokines/chemokines
    - Single-cell RNA-seq and bioinformatic analyses
- QUANTIFICATION AND STATISTICAL ANALYSIS

disease; MPG, Miller-Payne grades; LUSC, lung squamous cell carcinoma; LUAD, lung adenocarcinoma; LUASC, lung adenosquamous carcinoma; TLSs, tertiary lymphoid structures. ***$p < 0.001$. ns, not significant. One-way ANOVA.

(F and G) Computed tomography (CT) scan images of NSCLC patients in $ERO1A^{low}$ (F) and $ERO1A^{high}$ groups (G). CT scan was performed before (top) and after immunotherapy (bottom), respectively. IO, immunotherapy. Tumor is denoted by dotted line.

(H) PET-CT scan images of NSCLC patients in $ERO1A^{low}$ and $ERO1A^{high}$ groups before (top) and after immunotherapy (bottom). Tumor is denoted by dotted line. IO, immunotherapy; SUV, standardized uptake value.

(I) Changes in tumor size after neoadjuvant immunotherapy in $ERO1A^{low}$ (n = 15 patients) and $ERO1A^{high}$ groups (n = 22 patients). Tumor size was measured by CT scan and calculated by referring to the corresponding baseline.

(J) Changes in tumor burden after neoadjuvant immunotherapy between $ERO1A^{low}$ (n = 15 patients) and $ERO1A^{high}$ groups (n = 22 patients). Tumor burden is measured as tumor volume. ***$p < 0.001$. ns, not significant. Paired Student's t test.

(K) Relapse-free survival (RFS) for NSCLC patients stratified by the ERO1A expression. Kaplan-Meier curves of RFS for patients in $ERO1A^{low}$ (n = 15) and $ERO1A^{high}$ groups (n = 22). *$p < 0.05$. Log rank test.

(L) Multiplex IHC (mIHC) staining of CD4 (cyan), CD8 (violet), CD68 (red), ERO1A (green), IRE1a (orange), PanCK (white), and DAPI (blue) of lung tumors treated with immunotherapy (representative of n = 3 patients). The ERO1A expression stimulated different downstream tumor immune phenotypes. Scale bars, 200 μm.

Table 1. Baseline demographic and disease characteristics of the included patients with NSCLC

Clinical characteristic	ERO1A low	ERO1A high	p value
	(n = 15)	(n = 22)	
Gender (n, %)			0.431
Female	2 (13.3%)	6 (27.3%)	
Male	13 (86.7%)	16 (72.7%)	
Age (years)	62.5 ± 7.9	62.4 ± 9.1	0.891
Smoking			0.484
Current or former	8 (53.3%)	18 (81.8%)	
Never	7 (46.7%)	4 (18.2%)	
Pack/day	0.93 ± 0.46	1.05 ± 0.70	
Histology			0.357
Adenocarcinoma	3 (20.0%)	8 (36.4%)	
Squamous carcinoma	12 (80.0%)	13 (59.1%)	
Adenosquamous carcinoma	0 (0%)	1 (4.5%)	
Stage			0.126
IIA	2 (13.3%)	5 (22.7%)	
IIB	6 (40.0%)	2 (9.1%)	
IIIA	5 (33.3%)	13 (59.1%)	
IIIB	2 (13.3%)	2 (9.1%)	
Treatment			0.495
aPD-1	8 (53.3%)	13 (59.1%)	
aPD-1 + chemotherapy	7 (46.7%)	9 (40.9%)	
Clinical response			0.002
Partial response	13 (86.7%)	4 (18.2%)	
Stable disease	2 (13.3%)	14 (63.6%)	
Progression of disease	0 (0%)	4 (18.2%)	
Pathological response rate			0.158
≤30%	4 (26.7%)	7 (31.8%)	
30%–90%	4 (26.7%)	9 (40.9%)	
≥90%	7 (46.6%)	6 (27.3%)	
Tertiary lymphoid structures			0.232
Yes	6 (40.0%)	8 (36.4%)	
Immature	4 (26.7%)	3 (13.6%)	
None	5 (33.3%)	11 (50.0%)	

Thirty-seven NSCLC patients who received neoadjuvant immunotherapy were classified into two groups according to the immunohistochemistry (IHC) staining of ERO1A expression from resected tumor samples. IHC staining distinguished 15 patients with low ERO1A expression (IHC −/+) and 22 patients with high ERO1A expression (IHC ++/+++), which was evaluated by three pathologists. Two-sided Student's t test, chi-squared test, or one-way ANOVA.

SUPPLEMENTAL INFORMATION

Supplemental information can be found online at https://doi.org/10.1016/j.xcrm.2023.101206.

ACKNOWLEDGMENTS

We thank all the Wang lab members for technical support, the CAMS Key Laboratory of Translational Research on Lung Cancer of the National Cancer Center/National Clinical Research Center for Cancer/Cancer Hospital, Chinese Academy of Medical Sciences, and Peking Union Medical College. We thank all the doctors in the Medical Oncology and Pathological Oncology departments for their technical support. We appreciate the Chen and Liu labs at the Core Facilities of West China Hospital, Sichuan University, for their generous help in scRNA-seq. We also thank Jiayu Xu at Tongji University for her help in preparing figures. This study was supported by the National Key R&D Program of China (2022YFC2505000), the National Natural Science Foundation of China (82272796, 82241229, 82303317), the CAMS Innovation Fund for Medical Science (CIFMS 2022-I2M-1-009), and the CAMS Key Laboratory of Translational Research on Lung Cancer (2018PT31035).

AUTHOR CONTRIBUTIONS

Conceptualization, Jie Wang, H.B., and L.L.; methodology, L.L., S.L., Y.Q., X.P., and Jian Wang; investigation, L.L., S.L., Y.Q., X.P., Jian Wang, Z.W., J.D., J.Z., R.W., K.F., J.X., L.Y., P.X., and C.W.; resources, Z.W. and J.D.; data curation, L.L., X.P., X.Z., and Z.M.; writing – original draft, L.L., S.L., Y.Q., and X.P.; writing – review & editing, L.L., S.L., Y.Q., and X.P.; visualization, L.L. and X.P.; supervision, Jie Wang and H.B.; funding acquisition, L.L., Z.W., and Jie Wang.

DECLARATION OF INTERESTS

The authors declare no competing interests.

Received: March 21, 2023
Revised: July 24, 2023
Accepted: September 4, 2023
Published: September 27, 2023

REFERENCES

1. Friedrich, M.J. (2019). Immunotherapy 2.0: Improving the Response to Checkpoint Inhibitors. JAMA *321*, 131–133. https://doi.org/10.1001/jama.2018.18306.
2. Zhang, J., Huang, D., Saw, P.E., and Song, E. (2022). Turning cold tumors hot: from molecular mechanisms to clinical applications. Trends Immunol. *43*, 523–545. https://doi.org/10.1016/j.it.2022.04.010.
3. Binnewies, M., Roberts, E.W., Kersten, K., Chan, V., Fearon, D.F., Merad, M., Coussens, L.M., Gabrilovich, D.I., Ostrand-Rosenberg, S., Hedrick, C.C., et al. (2018). Understanding the tumor immune microenvironment (TIME) for effective therapy. Nat. Med. *24*, 541–550. https://doi.org/10.1038/s41591-018-0014-x.
4. Huang, A.C., and Zappasodi, R. (2022). A decade of checkpoint blockade immunotherapy in melanoma: understanding the molecular basis for immune sensitivity and resistance. Nat. Immunol. *23*, 660–670. https://doi.org/10.1038/s41590-022-01141-1.
5. O'Donnell, J.S., Teng, M.W.L., and Smyth, M.J. (2019). Cancer immunoediting and resistance to T cell-based immunotherapy. Nat. Rev. Clin. Oncol. *16*, 151–167. https://doi.org/10.1038/s41571-018-0142-8.
6. Waldman, A.D., Fritz, J.M., and Lenardo, M.J. (2020). A guide to cancer immunotherapy: from T cell basic science to clinical practice. Nat. Rev. Immunol. *20*, 651–668. https://doi.org/10.1038/s41577-020-0306-5.
7. Chen, X., and Cubillos-Ruiz, J.R. (2021). Endoplasmic reticulum stress signals in the tumour and its microenvironment. Nat. Rev. Cancer *21*, 71–88. https://doi.org/10.1038/s41568-020-00312-2.
8. Bettigole, S.E., and Glimcher, L.H. (2015). Endoplasmic reticulum stress in immunity. Annu. Rev. Immunol. *33*, 107–138. https://doi.org/10.1146/annurev-immunol-032414-112116.
9. Lin, J.H., Li, H., Yasumura, D., Cohen, H.R., Zhang, C., Panning, B., Shokat, K.M., Lavail, M.M., and Walter, P. (2007). IRE1 signaling affects cell fate during the unfolded protein response. Science *318*, 944–949. https://doi.org/10.1126/science.1146361.
10. Mandula, J.K., Chang, S., Mohamed, E., Jimenez, R., Sierra-Mondragon, R.A., Chang, D.C., Obermayer, A.N., Moran-Segura, C.M., Das, S.,

Vazquez-Martinez, J.A., et al. (2022). Ablation of the endoplasmic reticulum stress kinase PERK induces paraptosis and type I interferon to promote anti-tumor T cell responses. Cancer Cell *40*, 1145–1160.e9. https://doi.org/10.1016/j.ccell.2022.08.016.

11. Hurst, K.E., Lawrence, K.A., Essman, M.T., Walton, Z.J., Leddy, L.R., and Thaxton, J.E. (2019). Endoplasmic Reticulum Stress Contributes to Mitochondrial Exhaustion of CD8(+) T Cells. Cancer Immunol. Res. *7*, 476–486. https://doi.org/10.1158/2326-6066.Cir-18-0182.

12. Zhang, J., Yang, J., Lin, C., Liu, W., Huo, Y., Yang, M., Jiang, S.H., Sun, Y., and Hua, R. (2020). Endoplasmic Reticulum stress-dependent expression of ERO1L promotes aerobic glycolysis in Pancreatic Cancer. Theranostics *10*, 8400–8414. https://doi.org/10.7150/thno.45124.

13. May, D., Itin, A., Gal, O., Kalinski, H., Feinstein, E., and Keshet, E. (2005). Ero1-L alpha plays a key role in a HIF-1-mediated pathway to improve disulfide bond formation and VEGF secretion under hypoxia: implication for cancer. Oncogene *24*, 1011–1020. https://doi.org/10.1038/sj.onc.1208325.

14. Lei, Y., Zang, R., Lu, Z., Zhang, G., Huang, J., Liu, C., Wang, Z., Mao, S., Che, Y., Wang, X., et al. (2020). ERO1L promotes IL6/sIL6R signaling and regulates MUC16 expression to promote CA125 secretion and the metastasis of lung cancer cells. Cell Death Dis. *11*, 853. https://doi.org/10.1038/s41419-020-03067-8.

15. Liu, L., Wang, C., Li, S., Qu, Y., Xue, P., Ma, Z., Zhang, X., Bai, H., and Wang, J. (2021). ERO1L Is a Novel and Potential Biomarker in Lung Adenocarcinoma and Shapes the Immune-Suppressive Tumor Microenvironment. Front. Immunol. *12*, 677169. https://doi.org/10.3389/fimmu.2021.677169.

16. Pistillo, M.P., Carosio, R., Banelli, B., Morabito, A., Mastracci, L., Ferro, P., Varesano, S., Venè, R., Poggi, A., and Roncella, S. (2020). IFN-gamma upregulates membranous and soluble PD-L1 in mesothelioma cells: potential implications for the clinical response to PD-1/PD-L1 blockade. Cell. Mol. Immunol. *17*, 410–411. https://doi.org/10.1038/s41423-019-0245-x.

17. Ingram, J.R., Dougan, M., Rashidian, M., Knoll, M., Keliher, E.J., Garrett, S., Garforth, S., Blomberg, O.S., Espinosa, C., Bhan, A., et al. (2017). PD-L1 is an activation-independent marker of brown adipocytes. Nat. Commun. *8*, 647. https://doi.org/10.1038/s41467-017-00799-8.

18. Kumagai, S., Togashi, Y., Kamada, T., Sugiyama, E., Nishinakamura, H., Takeuchi, Y., Vitaly, K., Itahashi, K., Maeda, Y., Matsui, S., et al. (2020). The PD-1 expression balance between effector and regulatory T cells predicts the clinical efficacy of PD-1 blockade therapies. Nat. Immunol. *21*, 1346–1358. https://doi.org/10.1038/s41590-020-0769-3.

19. Liu, Y., Zhang, Q., Xing, B., Luo, N., Gao, R., Yu, K., Hu, X., Bu, Z., Peng, J., Ren, X., and Zhang, Z. (2022). Immune phenotypic linkage between colorectal cancer and liver metastasis. Cancer Cell *40*, 424–437.e5. https://doi.org/10.1016/j.ccell.2022.02.013.

20. Fucikova, J., Kepp, O., Kasikova, L., Petroni, G., Yamazaki, T., Liu, P., Zhao, L., Spisek, R., Kroemer, G., and Galluzzi, L. (2020). Detection of immunogenic cell death and its relevance for cancer therapy. Cell Death Dis. *11*, 1013. https://doi.org/10.1038/s41419-020-03221-2.

21. Park, S.J., Ye, W., Xiao, R., Silvin, C., Padget, M., Hodge, J.W., Van Waes, C., and Schmitt, N.C. (2019). Cisplatin and oxaliplatin induce similar immunogenic changes in preclinical models of head and neck cancer. Oral Oncol. *95*, 127–135. https://doi.org/10.1016/j.oraloncology.2019.06.016.

22. Galluzzi, L., Buqué, A., Kepp, O., Zitvogel, L., and Kroemer, G. (2017). Immunogenic cell death in cancer and infectious disease. Nat. Rev. Immunol. *17*, 97–111. https://doi.org/10.1038/nri.2016.107.

23. Lam, M., Marsters, S.A., Ashkenazi, A., and Walter, P. (2020). Misfolded proteins bind and activate death receptor 5 to trigger apoptosis during unresolved endoplasmic reticulum stress. Elife *9*, e52291. https://doi.org/10.7554/eLife.52291.

24. Lu, M., Lawrence, D.A., Marsters, S., Acosta-Alvear, D., Kimmig, P., Mendez, A.S., Paton, A.W., Paton, J.C., Walter, P., and Ashkenazi, A. (2014). Opposing unfolded-protein-response signals converge on death receptor 5 to control apoptosis. Science *345*, 98–101. https://doi.org/10.1126/science.1254312.

25. Chang, T.K., Lawrence, D.A., Lu, M., Tan, J., Harnoss, J.M., Marsters, S.A., Liu, P., Sandoval, W., Martin, S.E., and Ashkenazi, A. (2018). Coordination between Two Branches of the Unfolded Protein Response Determines Apoptotic Cell Fate. Mol. Cell *71*, 629–636.e5. https://doi.org/10.1016/j.molcel.2018.06.038.

26. Mahadevan, N.R., Rodvold, J., Sepulveda, H., Rossi, S., Drew, A.F., and Zanetti, M. (2011). Transmission of endoplasmic reticulum stress and pro-inflammation from tumor cells to myeloid cells. Proc. Natl. Acad. Sci. USA *108*, 6561–6566. https://doi.org/10.1073/pnas.1008942108.

27. Mahadevan, N.R., Anufreichik, V., Rodvold, J.J., Chiu, K.T., Sepulveda, H., and Zanetti, M. (2012). Cell-extrinsic effects of tumor ER stress imprint myeloid dendritic cells and impair CD8⁺ T cell priming. PLoS One *7*, e51845. https://doi.org/10.1371/journal.pone.0051845.

28. Zheng, Y., Chen, Z., Han, Y., Han, L., Zou, X., Zhou, B., Hu, R., Hao, J., Bai, S., Xiao, H., et al. (2020). Immune suppressive landscape in the human esophageal squamous cell carcinoma microenvironment. Nat. Commun. *11*, 6268. https://doi.org/10.1038/s41467-020-20019-0.

29. Tanaka, T., Kutomi, G., Kajiwara, T., Kukita, K., Kochin, V., Kanaseki, T., Tsukahara, T., Hirohashi, Y., Torigoe, T., Okamoto, Y., et al. (2016). Cancer-associated oxidoreductase ERO1-α drives the production of VEGF via oxidative protein folding and regulating the mRNA level. Br. J. Cancer *114*, 1227–1234. https://doi.org/10.1038/bjc.2016.105.

30. Liu, J., Fan, L., Yu, H., Zhang, J., He, Y., Feng, D., Wang, F., Li, X., Liu, Q., Li, Y., et al. (2019). Endoplasmic Reticulum Stress Causes Liver Cancer Cells to Release Exosomal miR-23a-3p and Up-regulate Programmed Death Ligand 1 Expression in Macrophages. Hepatology *70*, 241–258. https://doi.org/10.1002/hep.30607.

31. Cubillos-Ruiz, J.R., Silberman, P.C., Rutkowski, M.R., Chopra, S., Perales-Puchalt, A., Song, M., Zhang, S., Bettigole, S.E., Gupta, D., Holcomb, K., et al. (2015). ER Stress Sensor XBP1 Controls Anti-tumor Immunity by Disrupting Dendritic Cell Homeostasis. Cell *161*, 1527–1538. https://doi.org/10.1016/j.cell.2015.05.025.

32. Song, M., Sandoval, T.A., Chae, C.S., Chopra, S., Tan, C., Rutkowski, M.R., Raundhal, M., Chaurio, R.A., Payne, K.K., Konrad, C., et al. (2018). IRE1alpha-XBP1 controls T cell function in ovarian cancer by regulating mitochondrial activity. Nature *562*, 423–428. https://doi.org/10.1038/s41586-018-0597-x.

33. Obiedat, A., Seidel, E., Mahameed, M., Berhani, O., Tsukerman, P., Voutetakis, K., Chatziioannou, A., McMahon, M., Avril, T., Chevet, E., et al. (2019). Transcription of the NKG2D ligand MICA is suppressed by the IRE1/XBP1 pathway of the unfolded protein response through the regulation of E2F1. Faseb. J. *33*, 3481–3495. https://doi.org/10.1096/fj.201801350RR.

34. Lhomond, S., Avril, T., Dejeans, N., Voutetakis, K., Doultsinos, D., McMahon, M., Pineau, R., Obacz, J., Papadodima, O., Jouan, F., et al. (2018). Dual IRE1 RNase functions dictate glioblastoma development. EMBO Mol. Med. *10*, e7929. https://doi.org/10.15252/emmm.201707929.

35. Yang, Z., Huo, Y., Zhou, S., Guo, J., Ma, X., Li, T., Fan, C., and Wang, L. (2022). Cancer cell-intrinsic XBP1 drives immunosuppressive reprogramming of intratumoral myeloid cells by promoting cholesterol production. Cell Metabol. *34*, 2018–2035.e8. https://doi.org/10.1016/j.cmet.2022.10.010.

36. Wolchok, J.D., Chiarion-Sileni, V., Gonzalez, R., Grob, J.J., Rutkowski, P., Lao, C.D., Cowey, C.L., Schadendorf, D., Wagstaff, J., Dummer, R., et al. (2022). Long-Term Outcomes With Nivolumab Plus Ipilimumab or Nivolumab Alone Versus Ipilimumab in Patients With Advanced Melanoma. J. Clin. Oncol. *40*, 127–137. https://doi.org/10.1200/JCO.21.02229.

37. Dammeijer, F., van Gulijk, M., Mulder, E.E., Lukkes, M., Klaase, L., van den Bosch, T., van Nimwegen, M., Lau, S.P., Latupeirissa, K., Schetters, S., et al. (2020). The PD-1/PD-L1-Checkpoint Restrains T cell Immunity in

Tumor-Draining Lymph Nodes. Cancer Cell *38*, 685–700.e8. https://doi.org/10.1016/j.ccell.2020.09.001.

38. Limagne, E., Nuttin, L., Thibaudin, M., Jacquin, E., Aucagne, R., Bon, M., Revy, S., Barnestein, R., Ballot, E., Truntzer, C., et al. (2022). MEK inhibition overcomes chemoimmunotherapy resistance by inducing CXCL10 in cancer cells. Cancer Cell *40*, 136–152.e12. https://doi.org/10.1016/j.ccell.2021.12.0092.

39. Gide, T.N., Quek, C., Menzies, A.M., Tasker, A.T., Shang, P., Holst, J., Madore, J., Lim, S.Y., Velickovic, R., Wongchenko, M., et al. (2019). Distinct Immune Cell Populations Define Response to Anti-PD-1 Monotherapy and Anti-PD-1/Anti-CTLA-4 Combined Therapy. Cancer Cell *35*, 238–255.e6. https://doi.org/10.1016/j.ccell.2019.01.0033.

40. Jin, S., Guerrero-Juarez, C.F., Zhang, L., Chang, I., Ramos, R., Kuan, C.H., Myung, P., Plikus, M.V., and Nie, Q. (2021). Inference and analysis of cell-cell communication using CellChat. Nat. Commun. *12*, 1088. https://doi.org/10.1038/s41467-021-21246-9.

41. Love, M.I., Huber, W., and Anders, S. (2014). Moderated estimation of fold change and dispersion for RNA-seq data with DESeq2. Genome Biol. *15*, 550. https://doi.org/10.1186/s13059-014-0550-84.

42. Wang, Y., Wang, R., Zhang, S., Song, S., Jiang, C., Han, G., Wang, M., Ajani, J., Futreal, A., and Wang, L. (2019). iTALK: an R Package to Characterize and Illustrate Intercellular Communication. Preprint at bioRxiv507871. https://doi.org/10.1101/507871.

43. Ritchie, M.E., Phipson, B., Wu, D., Hu, Y., Law, C.W., Shi, W., and Smyth, G.K. (2015). limma powers differential expression analyses for RNA-sequencing and microarray studies. Nucleic Acids Res *43*, e47. https://doi.org/10.1093/nar/gkv0075.

44. Cao, J., Spielmann, M., Qiu, X., et al. (2019). The single-cell transcriptional landscape of mammalian organogenesis. Nature *566* (*7745*), 496–502. https://doi.org/10.1038/s41586-019-0969-x6.

45. R Core Team (2008). The R project for statistical computing (Vienna, Austria: T Team), Available online at https://www.R-project.org/.

46. Stuart, T., Butler, A., Hoffman, P., Hafemeister, C., Papalexi, E., Mauck, W.M., 3rd, Hao, Y., Stoeckius, M., Smibert, P., and Satija, R. (2019). Comprehensive Integration of Single-Cell Data. Cell *177*, 1888–1902.e21. https://doi.org/10.1016/j.cell.2019.05.031.

STAR★METHODS

KEY RESOURCES TABLE

REAGENT or RESOURCE	SOURCE	IDENTIFIER
**Antibodies**		
Anti-ERO1L	Abcam	Cat# ab177156; RRID: AB_2941804
Anti-IRE1α	Abcam	Cat# ab37073; RRID: AB_775780
Anti-phospho-IRE1α (S274)	Abcam	Cat# ab48187; RRID: AB_873899
Anti-PERK	Abcam	Cat# ab229912; RRID: AB_2941805
Anti-pan-Cytokeratin	Abcam	Cat# ab7753; RRID: AB_306047
Anti-human CD4	Abcam	Cat# ab133616; RRID: AB_2750883
Anti-human CD8	Abcam	Cat# ab4055; RRID: AB_304247
Anti-human CD68	Abcam	Cat# ab213363; RRID: AB_2801637
Anti-mouse CD4	Abcam	Cat# ab183685; RRID: AB_2686917
Anti-mouse CD8	Abcam	Cat# ab217344; RRID: AB_2890649
Anti-mouse CD161	Abcam	Cat# ab234107; RRID: AB_2922430
Anti-mouse FOXP3	Abcam	Cat# ab215206; RRID: AB_2860568
Anti-mouse Mannose Receptor	Abcam	Cat# ab64693; RRID: AB_1523910
Anti-mouse Ly6g	Abcam	Cat# ab238132; RRID: AB_2923218
Anti-mouse PD1	Abcam	Cat# ab214421; RRID: AB_2941806
Anti-mouse PD-L1	Abcam	Cat# ab213480; RRID: AB_2773715
Anti-IRE1α	Cell Signaling Technology	Cat# 3294; RRID: AB_823545
Anti-XBP1s	Cell Signaling Technology	Cat# 12782; RRID: AB_778939
Anti-phospho-PERK (Thr980)	Cell Signaling Technology	Cat# 3179; RRID: AB_2095853
Anti-eIF2α	Cell Signaling Technology	Cat# 9722; RRID: AB_ 2230924
Anti-phospho-eIF2α (Ser51)	Cell Signaling Technology	Cat# 9721; RRID: AB_330951
Anti-ATF4	Cell Signaling Technology	Cat# 11815; RRID: AB_2616025
Anti-GADD 153	Santa Cruz	Cat# sc-7351; RRID: AB_627411
Anti-ATF6α	Santa Cruz	Cat# sc-166659; RRID: AB_2058901
Anti-mouse CD3 FITC	Tonbo	Cat# 35-0032; RRID: AB_2621660
Anti-mouse CD4 APC	Tonbo	Cat# 20-0041; RRID: AB_2621543
Anti-mouse CD4 FITC	Tonbo	Cat# 35-0041; RRID: AB_2621665
Anti-mouse CD8a APC	Tonbo	Cat# 20-1886; RRID: AB_2621595
Anti-human/mouse CD11b APC	Tonbo	Cat# 20-0112; RRID: AB_2621556
Anti-human/mouse CD11b APC-Cyanine7	Tonbo	Cat# 25-0112; RRID: AB_2314132
Anti-mouse CD11c APC	Tonbo	Cat# 20-0114; RRID: AB_2621557
Anti-mouse CD11c PerCP-Cyanine5.5	Tonbo	Cat# 65-0114; RRID: AB_2621886
Anti-mouse CD19 APC	Tonbo	Cat# 20-0193; RRID: AB_2621562
Anti-mouse CD25 PE	Tonbo	Cat# 50-0251; RRID: AB_2621757
Anti-mouse CD45 PE	Tonbo	Cat# 50-0451; RRID: AB_2621763
Anti-mouse CD45 PE-Cyanine7	Tonbo	Cat# 60-0451; RRID: AB_2621848
Anti-mouse CD86 APC	Tonbo	Cat# 20-0862; RRID: AB_2621584
Anti-mouse Foxp3 APC	Tonbo	Cat# 20-0191; RRID: AB_2621561
Anti-mouse NK1.1 PE	Tonbo	Cat# 50-5941; RRID: AB_2621804
Anti-mouse Ly-6C PE	Tonbo	Cat# 50-5932; RRID: AB_2941807
Anti-mouse Ly-6G PerCP-Cyanine5.5	Tonbo	Cat# 65-1276; RRID: AB_2621899
Anti-mouse MHC Class II FITC	Tonbo	Cat# 35-5321; RRID: AB_2621715
Anti-mouse F4/80 Antigen PE	Tonbo	Cat# 50-4801; RRID: AB_2621795

(*Continued on next page*)

CellPress
OPEN ACCESS

Continued

REAGENT or RESOURCE	SOURCE	IDENTIFIER
Rat IgG2b isotype control APC	Tonbo	Cat# 20-4031; RRID: AB_2621596
Rat IgG2b isotype control FITC	Tonbo	Cat# 35-4031; RRID: AB_2621708
Rat IgG2b isotype control PE	Tonbo	Cat# 50-4031; RRID: AB_2621789
Rat IgG2b isotype control PE-Cyanine5	Tonbo	Cat# 55-4031; RRID: AB_2621822
Rat IgG2b isotype control PE-Cyanine7	Tonbo	Cat# 60-4031; RRID: AB_2621863
Rat IgG2b isotype control APC-Cyanine7	Tonbo	Cat# 25-4031; RRID: AB_2621633
Anti-mouse CD16/CD32 (Fc Shield)	Tonbo	Cat# 70-0161; RRID: AB_2621487
Anti-mouse CD206 APC	Biolegend	Cat# 141708; RRID: AB_10900231
Anti-mouse Ly-6G/Ly-6C (Gr-1) APC/Cyanine7	Biolegend	Cat# 108424; RRID: AB_2137485
Ultra-LEAF^{TM} Purified anti-mouse CD3ε	Biolegend	Cat# 100340; RRID: AB_11149115
Ultra-LEAF^{TM} Purified anti-mouse CD28	Biolegend	Cat# 102116; RRID: AB_11147170
*InVivo*Plus anti-mouse PD-1 (CD279)	BioXCell	Cat# BP0273; RRID: AB_2687796
*InVivo*Plus anti-mouse CD8α	BioXCell	Cat# BP0117; RRID: AB_10950145
*InVivo*Plus rat IgG2a isotype control	BioXCell	Cat# BP0089; RRID: AB_1107769
Bacterial and virus strains		
DH5α	TsingKe	Cat# TSC-C14
Biological samples		
Mouse samples	This paper	N/A
Human NSCLC samples	This paper	Cancer Hospital; Table 1
Chemicals, peptides, and recombinant proteins		
Cell Counting Kit-8	Dojindo	Cat# CK04
Cobalt chloride 0.1 M solution	Sigma-Aldrich	Cat# 15862-1ML-F
Collagenase	Sigma-Aldrich	Cat# C5138
Deoxyribonuclease I	Sigma-Aldrich	Cat# D4263
DMEM medium	Biological Industries	Cat# 06-1055-57-1A
DMSO	Sigma-Aldrich	Cat# D2650
DNase I	Sigma-Aldrich	Cat# D4263
DPBS	Gibco	Cat# 14190250
Fetal bovine serum	Gibco	Cat# 12662029
Kira6	Selleck Chemicals	Cat# S8658
Lipofectamine^{TM} 200 transfection reagent	Invitrogen	Cat# 11668500
Matrigel	Corning	Cat# 356237
Mouse IL-2 recombinant protein	Gibco	Cat# 212-12-1MG
PageRuler^{TM} prestained protein ladder	Thermo Fisher Scientific	Cat# 26616
Penicillin/Streptomycin	Gibco	Cat# 15140-122
PMSF 100mM	Beyotime	Cat# ST506
Phosphatase inhibitor cocktail A (50X)	Beyotime	Cat# P1081
Propidium Iodide solution	BD Bioscience	Cat# 556463; RRID: AB_2869075
Protease inhibitor	Beyotime	Cat# P1045
Protein kinase K	Solarbio	Cat# P9460
Puromycin	Selleck Chemicals	Cat# S7417
QuickBlock^{TM} primary antibody dilution buffer	Beyotime	Cat# P0256
QuickBlock^{TM} secondary antibody dilution buffer	Beyotime	Cat# P0258
RIPA buffer	Beyotime	Cat# P0013B
RPMI 1640 medium	Biological Industries	Cat# 01-100-1A
RPMI 1640 medium, no glucose	Gibco	Cat# 11879020
SDS-PAGE sample loading buffer (5X)	Beyotime	Cat# P0015
SuperSignal^{TM} West Pico PLUS chemiluminescent substrate	Thermo Fisher Scientific	Car# 34580

(*Continued on next page*)

Continued		
REAGENT or RESOURCE	SOURCE	IDENTIFIER
PowerUp^{TM} SYBR^{TM} Green master mix	Applied Biosystems	Cat# A25741
T7 Endonuclease I	Vazyme	Cat# EN303-01
Triton X-100 solution (10%)	Beyotime	Cat# ST797
Tris buffered saline tween	Solarbio	Cat# T1081
TRIzol	Invitrogen	Cat# 15596026
Trypsin EDTA solution	Gibco	Cat# 25200072
Tunicamycin	Sigma-Aldrich	Cat# HY-A0098
ProcartaPlex^{TM} Cell Lysis Buffer	Invitrogen	Cat# EPX-99999-000
Critical commercial assays		
17-Plex ProcartaPlex^{TM} immunoassay	Invitrogen	Cat# EPX170-26087-901
AlphaTSA® Multiplex IHC Kit	AlphaX	Cat# AXT36100031
Chromium Single Cell 3' Library and Bead Kit v2	10X Genomics	Cat# PN-120237
Chromium Single Cell 3' Chip Kit v2	10X Genomics	Cat# PN-120236
Chromium i7 Multiplex Kit	10X Genomics	Cat# PN-120262
Click-iT^{TM} EdU Cell Proliferation Kit	Invitrogen	Cat# C10340
CytoTox 96® Non-Radioactive Cytotoxicity Assay	Promega	Cat# G1780
EasySep^{TM} Mouse CD8^{+} T Cell Isolation Kit	STEMCELL	Cat# 19853_C
Mouse IFNγ ELISA Kit	Tianjin ANRC Bioscience	Cat# TAE-366m
Mouse TNFαELISA Kit	Tianjin ANRC Bioscience	Cat# TAE-569m
Mouse Gz-B ELISA Kit	Tianjin ANRC Bioscience	Cat# TAE-318h
Pierce^{TM} BCA Protein Assay Kit	Thermo Fisher Scientific	Cat# 23227
SDS-PAGE Gel Preparation Kit	Beyotime	Cat# P0012A
Vybrant^{TM} CFDA SE Cell Tracer Kit	Invitrogen	Cat# V12883
Deposited data		
ICI treated NSCLC cohort	Limagne et al. (2022)[38]	GEO: GSE190265
ICI treated melanoma cohort	Gide et al. (2019)[39]	ENA: PRJEB23709
scRNA-seq data of MC-38 tumors treated with aPD-1	This paper	GEO: GSE224525
TCGA LUAD, LUSC, BRCA, SKCM, LIHC, and READ datasets	TCGA data portal	https://www.cancer.gov/tcga
Experimental models: Cell lines		
B16-F10	ATCC	Cat# CRL-6475; RRID: CVCL_0159
Lewis lung carcinoma (LLC)	ATCC	Cat# CRL-1642; RRID: CVCL_4358
MC-38	Nanjing COBIOER Bioscience	Cat# CBP60825
Experimental models: Organisms/strains		
Mouse: C57BL/6	Jackson Lab	Cat# 000664
Mouse: BALB/c Nude	Beijing HFK Bioscience	Cat# 13001A
Oligonucleotides		
Ero1a sgRNA1 Forward	Zhang Lab; MIT	TTGGACTCCTGGGCGTCGTG
Ero1a sgRNA2 Forward	Zhang Lab; MIT	CTTAACCCTGAGCGCTACAC
Ero1a sgRNA3 Reverse	Zhang Lab; MIT	CTCCATATCCTCCAAGCGTC
RT-qPCR primers	MGH CCIB	Table S1
Recombinant DNA		
FV034-sgRNA	This paper	N/A
FV115-*Ero1a*-zsGreen	This paper	N/A
Software and algorithms		
Aperio ImageScope-12.4.6	Leica	https://www.leicabiosystems.com/zh-cn/digital-pathology/manage/aperio-imagescope/
CellChat	Jin et al. (2021)[40]	https://github.com/sqjin/CellChat

(*Continued on next page*)

Continued

REAGENT or RESOURCE	SOURCE	IDENTIFIER
Cell Ranger-5.0.1	10X Genomics	https://support.10xgenomics.com/singlecell-gene-expression/software
DESeq2	Love et al. (2014)[41]	https://bioconductor.org/packages/release/bioc/html/DESeq2.html
FlowJo v10.9	FlowJo LLC	https://www.flowjo.com
GraphPad Prism 9	GraphPad	https://www.graphpad.com/scienti?csoftware/prism/
ImageJ	ImageJ	https://imagej.nih.gov/ij/
iTALK	Wang et al. (2019)[42]	https://github.com/Coolgenome/iTALK
Limma-3.48.3	Ritchie et al. (2015)[43]	http://bioinf.wehi.edu.au/limma
Monocle3	Cao et al. (2019)[44]	https://cole-trapnell-lab.github.io/monocle3
R software-4.1.0	R Core Team (2008)[45]	http://www.r-project.org/
Seurat-3.2.0	Stuart et al. (2019)[46]	https://satijalab.org/seurat/
Zen-3.3	Carl Zesis AG	https://portal.zeiss.com/download-center/softwares/mic

RESOURCE AVAILABILITY

Lead contact

Further information and requests regarding this manuscript should be directed to and will be fulfilled by the lead contact Jie Wang (zlhuxi@163.com).

Materials availability

The authors declare that all the results supporting the findings of this study are available within the paper and its Supplemental Figures.

Data and code availability

- The single-cell RNA sequencing dataset in this study has been deposited to the NCBI Gene Expression Omnibus database and the accession number is GSE224525. Survival analyses of patients received immunotherapy were based on the transcriptome profiling combined with corresponding clinical data of ICI-treated patients with NSCLC (GEO: GSE190265) and melanoma (ENA: PRJEB23709).
- Correlations between the mRNA levels of ERO1A and immune markers were performed with data extracted from the TCGA project (https://portal.gdc.cancer.gov/). The correlations between ERO1A mRNA levels and patient survival were performed with data acquired from the Kaplan-Meier Plotter database (http://kmplot.com/analysis/). Correlation analyses of ERO1A mRNA levels and tumor-infiltrated lymphocytes were performed using the TIMER2.0 database (http://timer.comp-genomics.org). Additional codes used for processing and analysis is available upon request.
- Any additional information required to reanalyze the data reported in this paper is available upon request.

EXPERIMENTAL MODEL AND STUDY PARTICPANT DETAILS

Human lung cancer tissue imaging and specimens

This study was approved by the Ethical Research Committee of the National Cancer Center/National Clinical Research Center/Cancer Hospital, Chinese Academy of Medical Sciences and Peking Union Medical College (NCC2022C-804). All patients were research-consented for providing archival formalin-fixed and paraffin-embedded (FFPE) tissue blocks and radiological images. Inclusion criteria for the cohort includes patients pathologically confirmed with resectable lung cancer and received primary lung cancer surgical resection after two cycles of neoadjuvant immunotherapy at National Cancer Center between 2018 and 2022. All patients were treatment-naïve before receiving neoadjuvant treatment and were with three representative tissue blocks each.

H&E and IHC staining were performed in the Histopathology Department and Translational Lung Cancer Research Laboratory of National Cancer Center. Sections of 4-5 μm thickness were cut from FFPE tissue blocks for H&E and IHC staining. Slides were scanned using the Aperio Pathology Imaging System (Leica) and were viewed with ImageScope (Leica) which allows for 20× magnification of image captures. Slides were assessed for tumor region and pathological response by pathologists in National Cancer Center. Slides were scored as -\+\++\+++ by two pathologists independently, and any discrepancies were resolved through discussions

with the other pathologist. Slides with -/+ expression levels were characterized as ERO1A low expressors (15 patients) while ++/+++ expression levels were characterized as ERO1A high expressors (22 patients). The slides were also evaluated by IHC Profiler using ImageJ to independently confirm the manual scoring. Clinical response was evaluated after two cycles of neoadjuvant immunotherapy based on radiologic images by oncologists.

Mouse models and treatment

C57BL/6 and BALB/cA-nu mice were purchased from the Jackson Laboratory and bred in the specific pathogen-free animal facility at National Cancer Canter, Cancer Hospital. All animal experiments were approved by the Animal Care and Use Committee of National Cancer Center. 7-week-old female mice were selected at random and used for subsequent *in vivo* experiments. About 5×10^5 tumor cells were suspended with 100 μL PBS and then subcutaneously injected into the right flank of mice. Tumor volumes were measured every 2 days *via* vernier caliper and calculated with $0.5 \times \text{length} \times \text{width}^2$. The diameter of each single tumor was < 2.0 cm. Tumors were collected, washed, and weighted on the indicated days, and used for FCM, IHC, WB, and RNA-sequencing. For secondary-tumor challenge, C57BL/6 mice were first injected with PBS, $Ero1a^{WT}$, or $Ero1a^{KO}$ cells. Tumor cells were subcutaneously injected into the right flank of mice at a dose of 1×10^5 cells, and further challenged the mice with 5×10^5 $Ero1a^{WT}$ cells on the left flank after 10 days. From day 10, tumor size was measured every 2 days, and survival rate was determined every day by tumor length > 2.0 cm or animal death. For $CD8^+$ depletion, CD8 antibody (5 mg/kg per mouse) or the isotype control antibody (BioXCell, Cat# BP0117) was injected intraperitoneally for 4 consecutive days starting from day 1 after tumor transplantation, and every 5 days thereafter.

For MC-38 therapeutic models, MC-38 $Ero1a^{WT}$ or $Ero1a^{KO}$ tumors were subcutaneously transplanted into C57BL/6 mice. Mice were left for 10 days for tumor development and then allocated into 2 groups for isotype or aPD-1 treatment. For *in vivo* immunotherapy, 200 μg of anti-PD-1 antibody (BioXCell, Cat# BP0273) was injected intraperitoneally per mouse when xenograft tumor reached a palpable size, and every 3 days thereafter. Mice were sacrificed and analyzed after 6 cycles of treatment. In terms of Kira6 treatment, 10 mg/kg of Kira6 (Selleck Chemicals, Cat# S8658) was daily given through intraperitoneal injection for 14 days.

Cell culture

MC-38 (purchased from Nanjing COBIOER Biosciences, Cat# CBP60825), LLC (purchased from ATCC, Cat# CRL-1642), and B16-F10 (purchased from ATCC, Cat# CRL-6475) were cultured at 37°C with 5% CO_2 in RPMI-1640 (Biological Industries, Cat# 01-100-1A) supplemented with 10% fetal bovine serum (Gibco, Cat# 12662029) and 1% Penicillin-Streptomycin (Gibco, Cat# 15140-122). Cell lines were routinely tested for Mycoplasma by PCR.

METHOD DETAILS

IHC and mIHC staining

Slides with 4–5 μm tissue sections were baked at 60°C, deparaffinized with xylene and rehydrated through a graded series of ethanol solutions (100%, 95%, 85% and 75%). Tissue slides were then treated with microwave to induce epitope retrieval by boiling slides in critic acid solution for 15 min. Protein blocking was performed using blocking buffer (ZSGB-BIO, Cat# GT101510) for 20 min at room temperature. Primary antibodies for anti-ERO1L (Abcam, Cat# ab177156), anti-IRE1α (Abcam, Cat# ab37073), anti-PanCK (Abcam, Cat#ab7752), anti-CD4 (Abcam, Cat#ab133616), anti-CD8 (Abcam, Cat#ab4055), and anti-CD68 (Abcam, Cat#ab213363) were used. The slides were then incubated with secondary antibodies (HRP-anti-rabbit IgG, ZSGB-BIO, Cat# PV-6001; HRP-anti-mouse IgG, ZSGB-BIO, Cat# PV-6002) for 30 min at room temperature. Each slide was evaluated by 3 pathologists. For mIHC analysis of human samples, heat-induced epitope retrieval was performed to remove all the antibodies including primary and secondary antibodies after each cycle of staining. Multiplex immunofluorescence staining was performed using the AlphaTSA® Multiplex IHC Kit (AlphaX, Cat# AXT36100031). Slides were counterstained for nuclei with DAPI (ZSGB-BIO, Cat# ZLI-9957) for 10 min and mounted in mounting medium. Images were scanned and captured using ZEISS AXIOSCAN 7.

Construction of ERO1A knockout and expression plasmids

ERO1A was knocked out by CRISPR/Cas9 genome editing in mouse tumor cell lines, including MC-38, LLC, and B16-F10. Three sgRNAs were cloned into the lentiviral vector FV034. A CRISPR/Cas9 vector with non-targeting sgRNA (sgScramble) was used to establish corresponding control $Ero1a^{WT}$ tumor cells. HEK293T cells were transfected with lentiviral and helper vectors *via* Lipofectamine 2000 (Invitrogen, Cat# 11668500). Supernatant containing lentivirus was collected after 48h and used to infect tumor cells. Single clones were picked and expanded following puromycin (Selleck, Cat# S7417) selection. T7E1 (Vazyme, Cat# EN303-01) was performed for mutation validation. The HEK 293T cells (ATCC, Cat# CRL-1573) were routinely tested for Mycoplasma by PCR. sgRNAs sequences are as follows: sgEro1a1 (forward, TTGGACTCCTGGGCGTCGTG); sgEro1a2 (forward, CTTAACCC TGAGCGCTACAC); sgEro1a3 (reverse, CTCCATATCCTCCAAGCGTC). The mouse ERO1A cDNA (NM-015774.3) was used to generate full length ERO1A. Mouse ERO1A expression plasmids were used to rescue Ero1a expression in $Ero1a^{KO}$ tumors. cDNA expression validation was performed by qPCR and WB assays.

In vitro treatment and cell-death assays

For *in vitro* Kira6 treatment, MC-38 cells were first treated with 0.3 μg/mL Tunicamycin (Sigma-Aldrich, Cat# HY-A0098) to induce ER stress. After 12 h culturing, ER-stressed MC-38 cells were then treated 0.6 μM Kira6. The viability of cells was quantified using Cell Count Kit-8 (CCK-8) after treated for 24 or 48 h and calculated by normalizing to DMSO group (Dojindo, Cat# CK04). Cell death was measured through CytoTox 96® non-radioactive cytotoxicity assay (Promega, Cat# G1780) according to the manufacturer's instructions. Culture medium was collected and LDH release was measured at indicated time points.

Cell proliferation and migration assays

MC-38, LLC, or B16-F10 cells were seeded densely in a 6-well plate and cultured to confluence. Further, a 200-μL sterile tip was used to scratch a wound line across the monolayer cells. The detached cells were then washed away with phosphate-buffered saline. Cells were cultured in RPMI-1640 and photographed at 0- and 24-hour post-wounding. Images were captured using a phase-contrast microscope (OLYMPUS). Each assay was replicated thrice.

Migration assay was performed with a 24-well transwell chamber without Matrigel (Corning, Cat# 356237) coated in the upper chamber, while invasion assay was performed with its upper chamber coated with Matrigel. A total of 2 × 10^5 (invasion) or 10^5 (migration) MC-38, LLC, or B16-F10 tumor cells were seeded in the upper chamber with 200 μL of serum-free RPMI-1640. Then 700 μL of medium containing 10% FBS was added in the lower chamber. Cells on the upper membrane were carefully removed with a cotton swab after incubation for 24 h, and the invaded cells that had traversed the membrane were identified by crystal violet staining and photographed. Invaded cells were counted manually and confirmed by using ImageJ software (NIH).

T cell isolation and cytotoxic analysis

Lymphocytes were first collected from C57BL/6 naive mouse spleen and $CD8^+$ T cells were then isolated with $CD8^+$ negative selection kit (STEMCELL, Cat# 19853) according to the manufacturer's instructions. The isolated T cells were stimulated with anti-CD3 (2 μg/mL; Tonbo, Cat# 35-0032) and anti-CD28 (2 μg/mL; Tonbo, Cat# 20-0041) antibodies for 24 h in the presence of IL-2 (10 mg/mL; Gibco, Cat# 212-12-1MG), and the function of $CD8^+$ T cells was measured by quantifying the release of IFN-γ (Tianjin ANRC Bioscience, Cat# TAE-366m), TNF-α (Tianjin ANRC Bioscience, Cat# TAE-569m), and GzmB (Tianjin ANRC Bioscience, Cat# TAE-318h) by enzyme linked immunosorbent assay assays.

To further assess the cytotoxicity of $CD8^+$ T cells, about 1 × 10^6 MC-38 cells were dissociated and first stained with 1mL CFDA-SE (10 μM; Invitrogen, Cat# V12883) according to the manufacturer's instructions. The labelled MC-38 cells (1 × 10^5) were then co-cultured with T cells (5 × 10^6) for 24 h. To measure cell death in MC-38 cells, samples were collected and then stained with propidium iodide (BD Bioscience, Cat# 556463) and analyzed by LSR II (BD Biosciences) using FlowJo V10.9 software. CFDA-SE and PI dual-positive cells were determined as dead MC-38 cells.

Western blotting

Cell or tissue lysates were extracted in RIPA buffer (Beyotime, Cat# P0013B) Protein concentration was evaluated by bicinchoninic acid (BCA) Protein Assay Kit (Thermo Fisher Scientific, Cat# 23227) and then analyzed by SDS–PAGE gel electrophoresis and blotting onto PVDF membranes. Primary antibodies used included: anti-ERO1L (Abcam, Cat# ab177156), anti-IRE1α (Abcam, Cat# ab37073), anti-pIRE1α (Abcam, Cat# ab48187), anti-XBP1s (Cell Signaling Technology, Cat# 12782), anti-PERK (Abcam, Cat# ab229912), anti-pPERK (Cell Signaling Technology, Cat# 3179), anti-eIF2α (Cell Signaling Technology, Cat# 9722), anti-peIF2α (Cell Signaling Technology, Cat# 9721), anti-ATF4 (Cell Signaling Technology, Cat# 11815), anti-ATF6α (Santa Cruz, Cat# sc-166659), anti-CHOP (Santa Cruz, Cat# sc-7351), and anti-β-Actin. Primary antibodies were applied in 5% non-fatty milk or primary antibody dilution (Beyotime, Cat# P0256) in TBST and incubated overnight at 4°C, followed by HRP-conjugated secondary antibodies incubation at room temperature for 2 h. Secondary antibodies used included: Goat anti-Rabbit HRP-conjugated IgG (Abcam, Cat# ab6721), Rabbit anti-Mouse HRP-conjugated IgG (Abcam, Cat# ab6728). Images were collected by Amersham Imager 600 (General Electric).

Antibody staining and flow cytometry

To quantify the abundance of TILs in tumor samples, fresh tumor lysates were stained with conjugated antibodies and isotype controls. Antibodies used for FCM are listed in the key resource table. For intracellular staining, cells were first stained with antibodies to cell-surface markers for 30 min, then fixed and permeabilized with fixation/permeabilization buffer and stained with Foxp3-APC (Tonbo, Cat# 20-0191). After staining, immunocytes analysis was performed on LSR II (BD Biosciences).

Quantitative RT-PCR

Total RNA was isolated from T cells using TRIzol reagent (Invitrogen, Cat# 15596026). Reverse transcription was performed using M-MLV Reverse Transcriptase (Invitrogen, Cat# 28025013) under the manufacturer's instructions. RT-qPCR reactions were performed using PowerUp™ SYBR™ Green master mix (Applied Biosystems, Cat# A25741) in QuantStudio™ 5 (Applied Biosystems, Cat# A28140).

Enzyme-linked immunosorbent assay

To assess the function of T cells, the release of IFN-γ, TNF-α, and GzmB were analyzed by ELISA. After co-culturing of tumor cells with activated $CD8^+$ T cells for 24 h, the supernatant was collected and filtered to prepare the samples to be tested. Protein concentrations were measured in the supernatant using the BCA methods. ELISA was performed through mouse-IFN-γ, -TNF-α, and -GzmB ELISA Kits according to the manufacturer's instructions. Binding signals were detected at 450 nm using a 96-well plate reader (Thermo Fisher Scientific).

Luminex-based multiplexing of cytokines/chemokines

To profile the cytokines and chemokines in tumor samples, the 17-Plex ProcartaPlex™ immunoassay (Invitrogen, Cat# EPX170-26087-901) was performed under the manufacturer's instructions. Fresh tumor tissues were weighted and prepared for extraction of suspension proteins. Tumor tissues were then subjected to homogenization with 1 mm glad beads of 60 s in ProcartaPlex™ Cell Lysis Buffer (Invitrogen, Cat# EPX-99999-000) in the FastPrep-24 5G benchtop reciprocating homogenizer (MP Biomedicals, Cat# 116005500). Protein concentration was measured in the tumor lysates using the BCA methods. Values were normalized based on protein concentration.

Single-cell RNA-seq and bioinformatic analyses

The scRNA library of samples was prepared and constructed by using Chromium Single Cell 3′ Reagent kits followed by the manufacturer's protocol (10X Genomics). The Illumina NovaSeq 6000 platform was used to sequence the libraries of scRNA samples with pair-end 150 base pairs. The CellRanger (v 5.0.1) was used to align the clean reads with mm10. The *Seurat*[46] (v 3.2.0) pipeline was integrated into analysis and visualization, including clustering, dimension reduction, and cell type identification. The genes expressed in less than three cells were removed. The cells that expressed less than 200 genes were not included in the analysis. The miQC, which considers the correlations between UMIs and mitochondrial gene ratio in scRNA data, was used to filter low-quality single-cell data with parameters of model_type = "spline" and posterior_cutoff = 0.75. The DoubletFinder was used to remove the doublet single-cell data with parameters of PCs = 1:20, pN = 0.25, and pK = 0.09. The 29,820 cells finally were retained for further analysis.

The harmony was used to remove the batch effects between samples. The dimension reducing of tsne, phate and umap were calculated based on 20 harmony components. The phate module was implemented by the package of reticulate and s2a. The cell types were identified by classical molecular markers and visualized by *ComplexHeatmap*. The density of distribution of scRNA was performed by stat_density_2d function with the parameters of geom = "tile", aes (fill = ..density..), contour = FALSE. The significantly up-regulated genes in the specific sample and/or subpopulation were identified by FindMarkers and FindAllMarkers. The *clusterProfiler* was used to annotate the top markers of each subpopulation with the KEGG database. The gene set enrichment analysis was also performed by the GSEA function implemented in *clusterProfiler*. The slingshot was used to estimate the development trajectory and order the single-cell data. The differentialGeneTest implemented in *monocle2* was used to identify the dynamics expressing genes among the development trajectories. The genes whose q-value $< 1 \times 10^{-10}$ were retained for analysis. The *genSmoothCurves* was used to downsample and smooth the expression data for accelerating the visualization and gene module cluster among the development trajectories. The *ggplot2* was used to display the expression patterns of gene modules.

The *iTALK*[42] and *CellChat*[40] were jointly used to quantify and exhibit the cell-cell interaction of scRNA data. The *filterCommunication* was used to filter the interaction of ligands and receptors with the parameter of min.cells = 10. The SPP1 signaling interaction was annotated and generated in CellChatDB.mouse. The *ggpubr* was used to generate bar plots and box plots for displaying the expression pattern of gene expression and gene set signatures with p value calculation. The statistical significance was denoted as *$p < 0.05$, **$p < 0.01$, and ***$p < 0.001$.

QUANTIFICATION AND STATISTICAL ANALYSIS

Two-tailed Student's t-tests were used to determine mean differences between two groups. Two-sided Chi-square tests were conducted to compare the difference in rate between two groups. Two-way ANOVA was used to compare differences among multiple groups. Survival curves were analyzed by log-rank analysis. Statistical analyses were performed using GraphPad Prism (GraphPad Software, Inc.). Data are presented as mean ± s.e.m or mean ± SDs. Statistical significance was determined as indicated in the figure legends. P values of less than 0.05 were considered significant; *$p < 0.05$, **$p < 0.01$, and ***$p < 0.001$.

Cell Reports Medicine, Volume *4*

Supplemental information

Ablation of ERO1A induces lethal endoplasmic reticulum stress responses and immunogenic cell death to activate anti-tumor immunity

Lihui Liu, Sini Li, Yan Qu, Hua Bai, Xiangyu Pan, Jian Wang, Zhijie Wang, Jianchun Duan, Jia Zhong, Rui Wan, Kailun Fei, Jiachen Xu, Li Yuan, Chao Wang, Pei Xue, Xue Zhang, Zixiao Ma, and Jie Wang

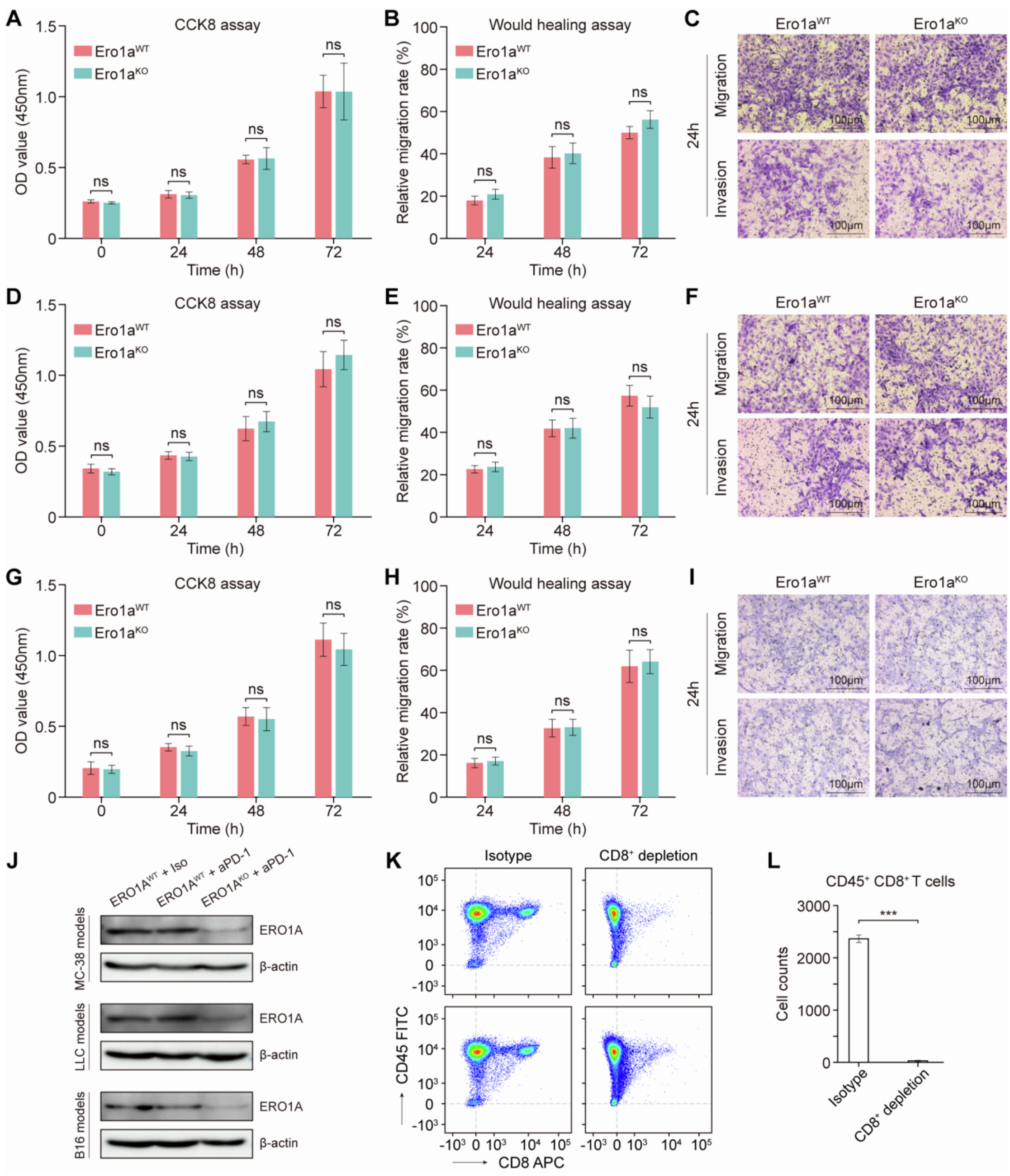

Figure S1: ERO1A deficiency affects the anti-tumor efficacy of immunotherapy but not the proliferation of tumor cells, related to Figure 1.

(A-C) Deletion of *Ero1a* in MC-38 cells did not impede cell proliferation (A), migration (B), and invasion (C) *in vitro* (n = 3 independent repeats). Data presented as means ± SEMs. ns, not significant. Two-sided Student's t test. Scale bars, 100 μm.

(D-F) Deletion of *Ero1a* in B16 cells did not impede cell proliferation (D), migration (E), and invasion (F) *in vitro* (n = 3 independent repeats). Data presented as means ± SEMs. ns, not significant. Two-sided Student's t test. Scale bars, 100 μm.

(G-I) Deletion of *Ero1a* in LLC cells did not impede cell proliferation (G), migration (H), and invasion

(I) *in vitro* (n = 3 independent repeats). Data presented as means ± SEMs. ns, not significant. Two-sided Student's t test. Scale bars, 100 μm.

(J) Western blotting plots of ERO1A expression levels in MC-38, LLC, and B16 therapeutic models. Result is a representative finding for 3 experiments.

(K and L) Flow cytometry (FCM) analysis of periphery blood from immunocompetent C57 mice after depletion of $CD8^+$ via antibody-based approaches. FCM plots of $CD8^+$ T cells in tumor-bearing mice treated with anti-$CD8^+$ antibody or isotype control (K). Box plot showing the quantitative cell numbers of $CD8^+$ T cells based on the FCM analysis (L). Representative of n = 3 mice/group. Data presented as means ± SEMs. ***$p < 0.001$. Two-sided Student's t test.

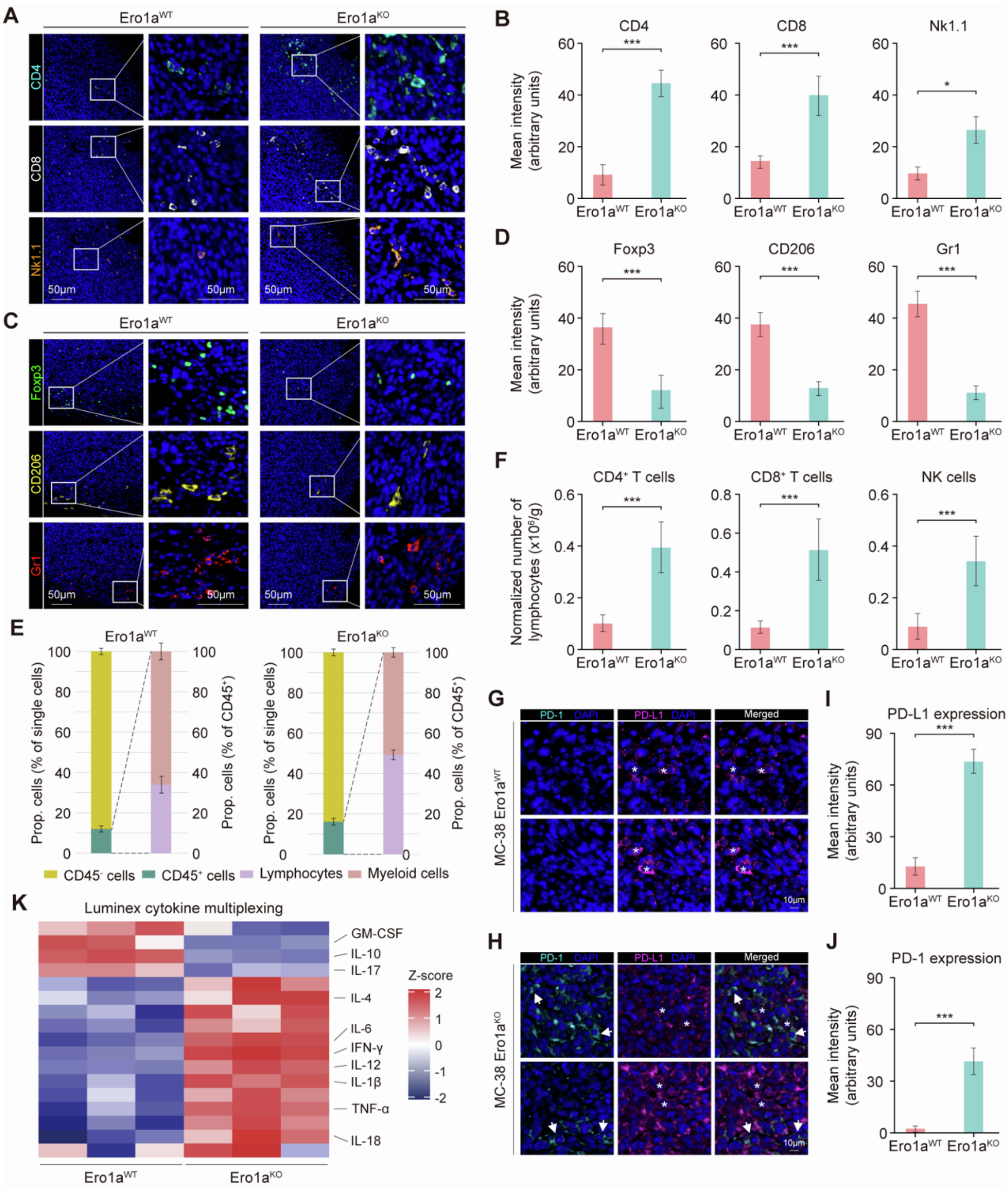

Figure S2: Immunophenotyping of ERO1A^{WT} and ERO1A^{KO} tumors in MC-38 therapeutic models, related to Figure 1.

(A and B) Immunofluorescence (IF) staining of CD4^{+} T cells (cyan), CD8^{+} T cells (white), Nk1.1^{+} NK cells (orange), and DAPI (blue) in the core region of Ero1a^{WT} and Ero1a^{KO} tumors (A). Box plots comparing the mean intensity of CD4^{+} T cells, CD8^{+} T cells, and NK cells in Ero1a^{WT} and Ero1a^{KO} tumors based on the IF staining (B). Data presented as means ± SDs from 6 randomly selected fields. *$p < 0.05$, ***$p < 0.001$. Two-sided Student's t test. Scale bars, 50 μm.

(C and D) IF staining of Foxp3^{+} Tregs (green), CD206^{+} macrophages (yellow), Gr1^{+} MDSCs (red), and DAPI (blue) in the core region of Ero1a^{WT} and Ero1a^{KO} tumors (C). Box plots comparing the mean

intensity of $Foxp3^+$ Tregs, $CD206^+$ macrophages, and $Gr1^+$ MDSCs in $Ero1a^{WT}$ and $Ero1a^{KO}$ tumors based on the IF staining (D). Data presented as means ± SDs from 6 randomly selected fields. ***$p < 0.001$. Two-sided Student's t test. Scale bars, 50 μm.

(E and F) FCM analysis of $CD45^+$ immunocytes, lymphocytes, and myeloid cells in $Ero1a^{WT}$ and $Ero1a^{KO}$ tumors (E). Box plots showing the quantitative cell numbers of tumor-infiltrating lymphocytes normalized by tumor weight, including $CD4^+$ T, $CD8^+$ T, and NK cells, based on FCM analysis (F). Representative of n = 6 mice/group. Data presented as means ± SEMs. ***$p < 0.001$. Two-sided Student's t test.

(G and H) IF staining of PD-L1 (violet), PD-1 (cyan) and DAPI (blue) in $Ero1a^{WT}$ (G) and $Ero1a^{KO}$ tumors (H). Tumors were collected after 2 rounds of aPD-1 treatment. Data represented for 3 randomly selected fields. Scale bars, 10 μm.

(I and J) Box plots comparing the mean intensity of PD-L1 (I) and PD-1 (J) in $Ero1a^{WT}$ and $Ero1a^{KO}$ tumors based on the IF staining. Data presented as means ± SDs from 6 randomly selected fields. ***$p < 0.001$. Two-sided Student's t test.

(K) Heatmap showing the secretion levels of cytokines/chemokines in $Ero1a^{WT}$ and $Ero1a^{KO}$ tumors, measured by Luminex-based multiplexing (n = 3 mice/group).

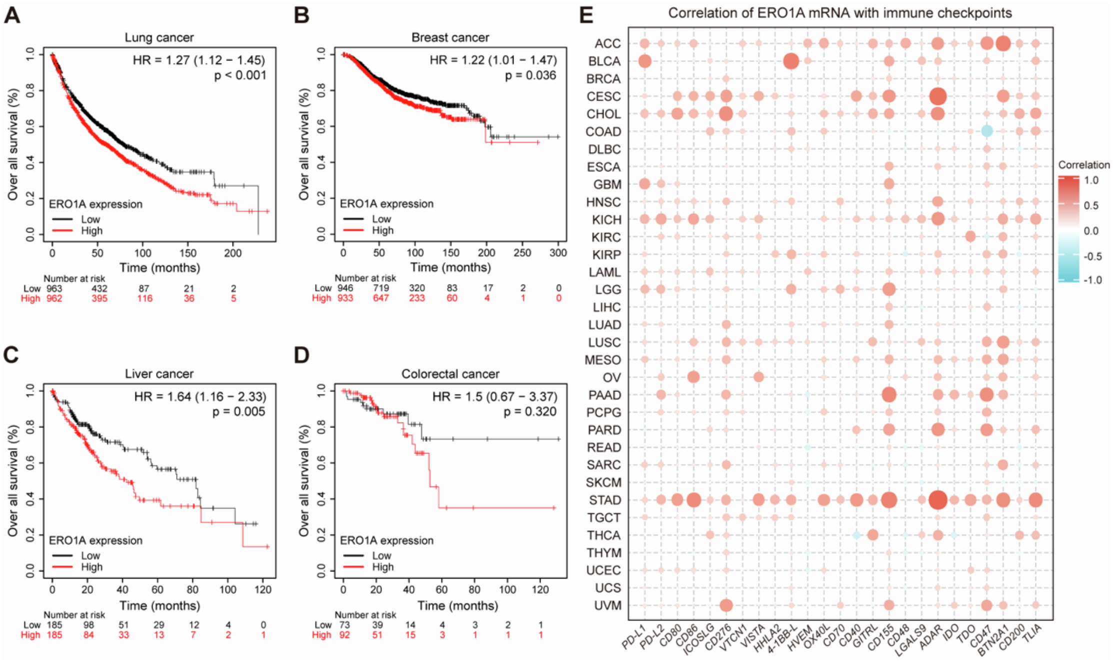

Figure S3: Correlation between ERO1A expression and clinical outcomes in patients with solid tumors, related to Figure 2.

(A-D) Survival curves showing that ERO1A was associated with worse overall survival (OS) of TCGA patients with lung cancer (A), breast cancer (B), liver cancer (C), and colorectal cancer (D). Log-rank test.

(E) Scatter plots showing the correlations of ERO1A mRNA and expressions of immune checkpoints in pan-cancers, according to the TCGA-based analyses. ACC, adrenocortical carcinoma. BLCA, bladder urothelial carcinoma. BRCA, breast invasive carcinoma. CESC, cervical squamous cell carcinoma. CHOL, cholangio carcinoma. COAD, colon adenocarcinoma. DLBC, diffuse large B-cell lymphoma. ESCA, esophageal carcinoma. GBM, glioblastoma multiforme. HNSC, head and neck squamous cell carcinoma. KICH, kidney chromophobe. KIRC, kidney renal clear cell carcinoma. KIRP, kidney renal papillary cell carcinoma. LAML, acute myeloid leukemia. LGG, brain lower grade glioma. LIHC, liver hepatocellular carcinoma. LUAD, lung adenocarcinoma. LUSC, lung squamous cell carcinoma. MESO, mesothelioma. OV, ovarian serous cystadenocarcinoma. PAAD, pancreatic adenocarcinoma. PCPG, pheochromocytoma and paraganglioma. PRAD, prostate adenocarcinoma. READ, rectum adenocarcinoma. SARC, sarcoma. SKCM, skin cutaneous melanoma. STAD, stomach adenocarcinoma. TGCT, testicular germ cell tumors. THCA, thyroid carcinoma. THYM, thymoma. UCEC, uterine corpus endometrial carcinoma. UCS, uterine carcinosarcoma. UVM, uveal melanoma. Pearson correlation test.

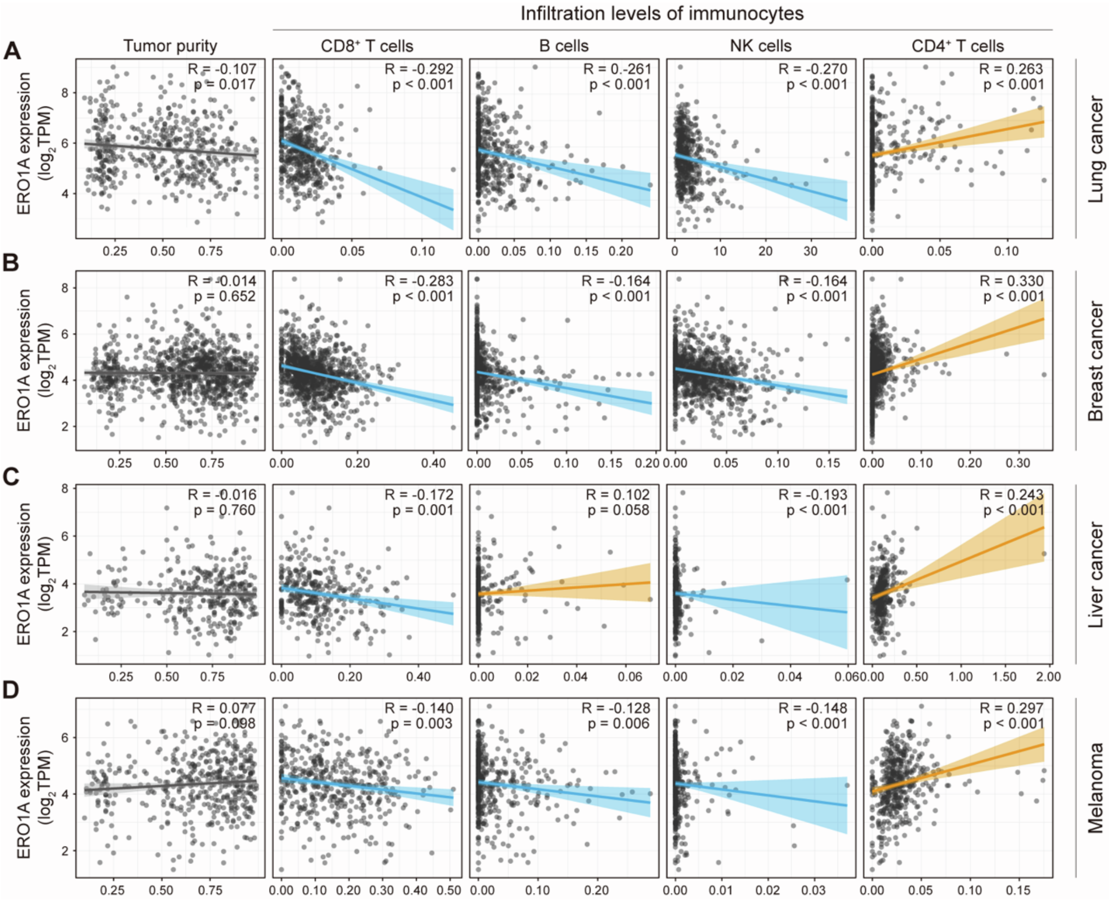

Figure S4: ERO1A is correlated with immune-suppressive tumor microenvironment in patients with solid tumors, related to Figure 2.

(A) Scatter plots showing correlations of ERO1A expression with $CD4^+$ T cells, $CD8^+$ T cells, B cells, and NK cells in TCGA LUAD data based on their signature gene expressions. Pearson correlation test.

(B) Scatter plots showing correlations of ERO1A expression with $CD4^+$ T cells, $CD8^+$ T cells, B cells, and NK cells in TCGA BRCA data based on their signature gene expressions. Pearson correlation test.

(C) Scatter plots showing correlations of ERO1A expression with $CD4^+$ T cells, $CD8^+$ T cells, B cells, and NK cells in TCGA LIHC data based on their signature gene expressions. Pearson correlation test.

(D) Scatter plots showing correlations of ERO1A expression with $CD4^+$ T cells, $CD8^+$ T cells, B cells, and NK cells in TCGA SKCM data based on their signature gene expressions. Pearson correlation test.

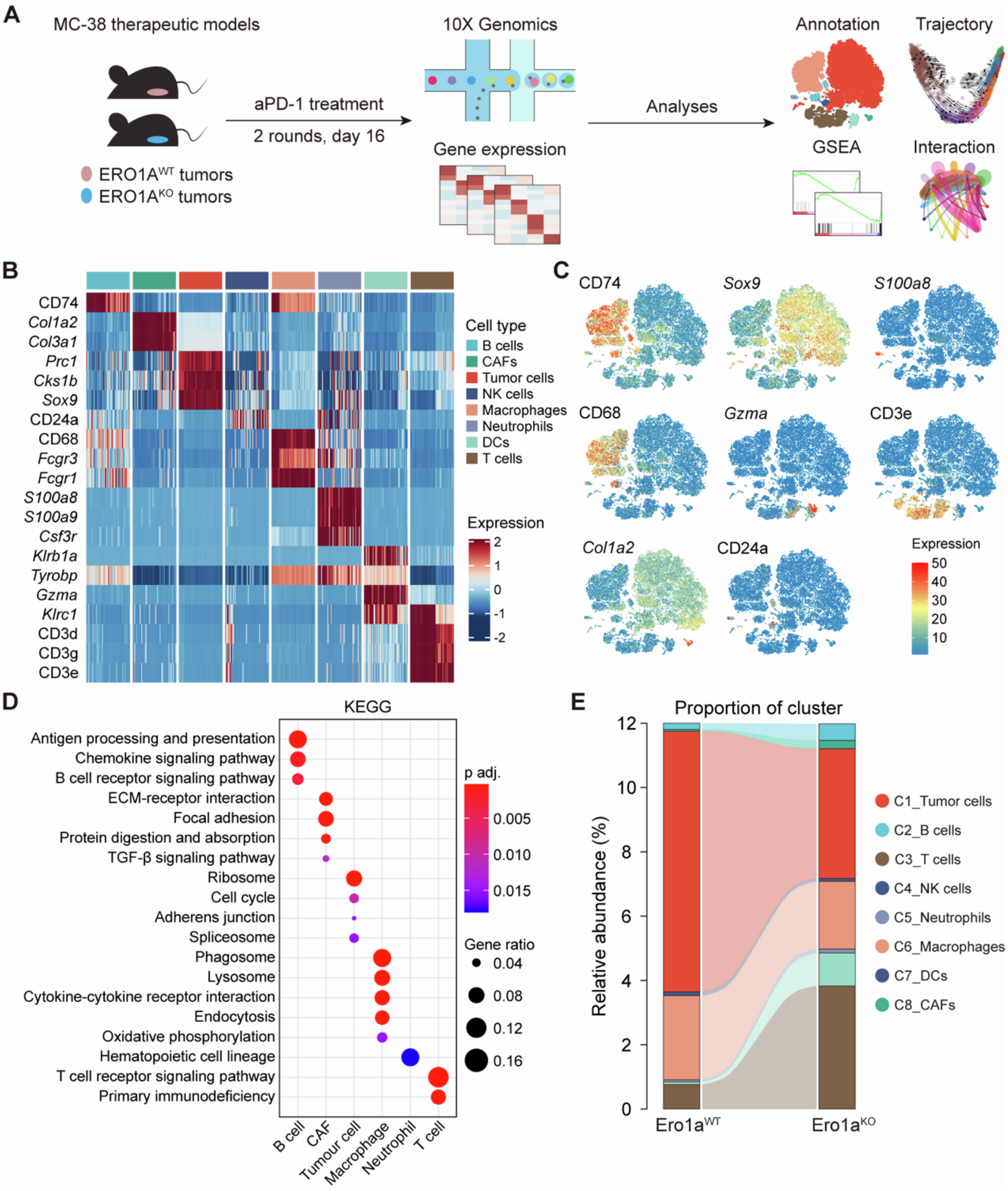

Figure S5: Single-cell RNA-seq analyses of cell populations in ERO1A^{WT} and ERO1A^{KO} tumors treated with aPD-1 blockade, related to Figure 2.

(A) Schematic overview of the scRNA-seq experimental design. Tumors were transplanted on day 0 and aPD-1 treatment was administered every 3 days from day 10. Ero1a^{WT} and Ero1a^{KO} tumors from MC-38 therapeutic models were collected after 2 rounds of aPD-1 treatment on day 16.

(B) Heatmap showing the expression levels of marker genes across major cell types, measured by scRNA-seq. CAFs, cancer-associated fibroblasts. DCs, dendritic cells. NK, natural killer.

(C) t-SNE map showing the marker gene expressions of major cell types based on the scRNA-seq data. Colored by cell subtypes.

(D) KEGG enrichment plot of major cell types based on scRNA-seq data.

(E) Sankey plot comparing the relative abundance (%) of major cell types between Ero1a^{WT} and Ero1a^{KO} tumors based on scRNA-seq data, colored by cell subtypes. CAFs, cancer-associated fibroblasts. DCs, dendritic cells. NK, natural killer.

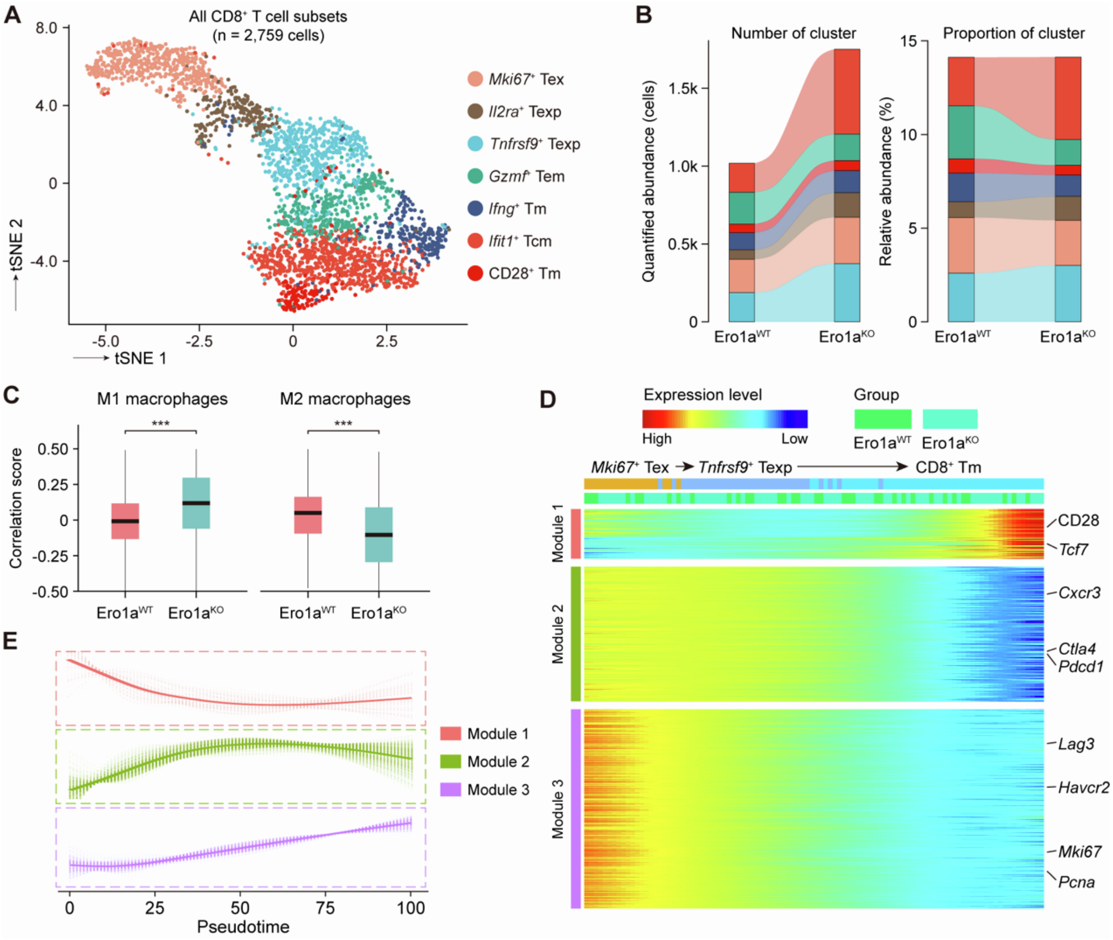

Figure S6: Immunophenotyping of ERO1A-associated tumor microenvironment remodeling in response to immunotherapy, related to Figure 2.

(A) t-SNE map showing the CD8$^+$ T cell clusters based on the scRNA-seq data. Colored by cell subtypes.

(B) Sankey plots comparing the quantified numbers (cells) relative abundance (%) of major cell types between ERO1A^{WT} and ERO1A^{KO} tumors based on scRNA-seq data, colored by cell subtypes.

(C) Box plots comparing the correlation scores of M1 and M2 macrophage signatures in Ero1a^{WT} and Ero1a^{KO} tumors based on their signature gene expressions, measured by scRNA-seq. Data presented as means ± SEMs. ***$p < 0.001$. Two-sided Student's t test.

(D and E) Trajectory analysis of CD8$^+$ T cells based on the scRNA-seq data in MC-38 therapeutic models. The dynamically expressed gene on the trajectory of CD8$^+$ T cells in Ero1a^{WT} and Ero1a^{KO} tumors were identified and group into 3 modules (D). The pseudotime expression changes of each module in CD8$^+$ T cells (E).

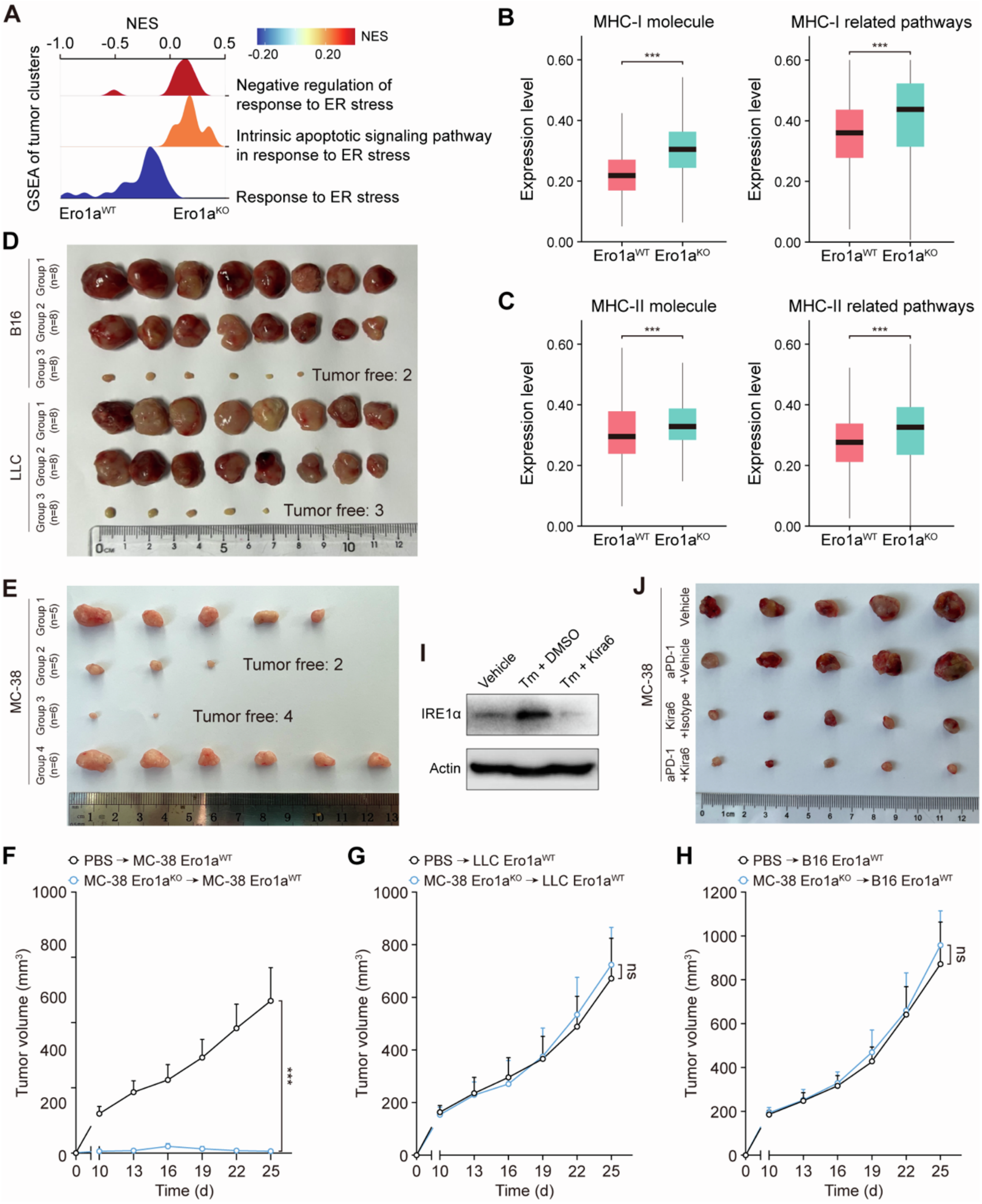

Figure S7: ERO1A deficiency triggers immunogenic cell death due to unresolved ER stress, related to Figures 3 and 4.

(A) GSEA of tumor cell subsets showing higher enrichment of negative regulation of ER stress response and apoptotic signaling pathway in Ero1a^{KO} tumors, compared to those in Ero1a^{WT} tumors.

(B) Box plots showing the comparisons of MHC-I molecule expression and MHC-I related pathways in Ero1a^{WT} and Ero1a^{KO} tumors, based on the scRNA-seq data in MC-38 therapeutic models. Data presented as means ± SEMs. ***p < 0.001. Two-sided Student's t test.

(C) Box plots showing the comparisons of MHC-II molecule expression and MHC-II related pathways in Ero1a^{WT} and Ero1a^{KO} tumors, based on the scRNA-seq data in MC-38 therapeutic models. Data

presented as means ± SEMs. ***p < 0.001. Two-sided Student's t test.

(D) Right flank tumor growth from rechallenged mice bearing B16 $Ero1a^{WT}$ (upper) or LLC $Ero1a^{WT}$ tumors (bottom). First challenge was with PBS (Group 1), $Ero1a^{WT}$ (Group 2), or $Ero1a^{KO}$ (Group 3) tumor cells on the left flank of C57CL/6 mice and rechallenge was performed after 10 days with $Ero1a^{WT}$ tumor cells on the right flank (n = 8 mice/group).

(E) Right flank tumor growth from rechallenged mice bearing MC-38 $Ero1a^{WT}$ tumors. First challenge was with PBS (Group 1, n = 5), $Ero1a^{WT}$ (Group 2, n = 5), $Ero1a^{KO}$ (Group 3, n = 6), or $Ero1a^{OE}$ (Group 4, n = 6) tumor cells on the left flank of C57CL/6 mice and rechallenge was performed after 10 days with MC-38 $Ero1a^{WT}$ tumor cells on the right flank.

(F) Tumor volume in mice bearing MC-38 $Ero1a^{WT}$ tumors in naïve mice (PBS pre-treated) or mice that had previously injected with MC-38 $Ero1a^{KO}$ cells (n = 6 mice/group). Data presented as means ± SEMs. ***p < 0.001. Two-sided Student's t test.

(G and H) Tumor volumes in mice bearing LLC $Ero1a^{WT}$ (G) and B16 $Ero1a^{WT}$ (H) tumors in naïve mice (PBS pre-treated) or mice that had previously injected with MC-38 $Ero1a^{KO}$ cells (n = 6 mice/group). Data presented as means ± SEMs. ns, not significant. Two-sided Student's t test.

(I) Western blotting plots of IRE1α in MC-38 $Ero1a^{WT}$ tumor cells treated with Kira6 *in vitro*. Result is a representative finding for 3 experiments.

(J) Tumor growth in C57BL/6 mice bearing MC-38 $Ero1a^{WT}$ tumors, treated with vehicle, aPD-1 plus vehicle, Kira6 plus isotype, or Kira6 plus aPD-1 blockade (n = 5 mice/group).

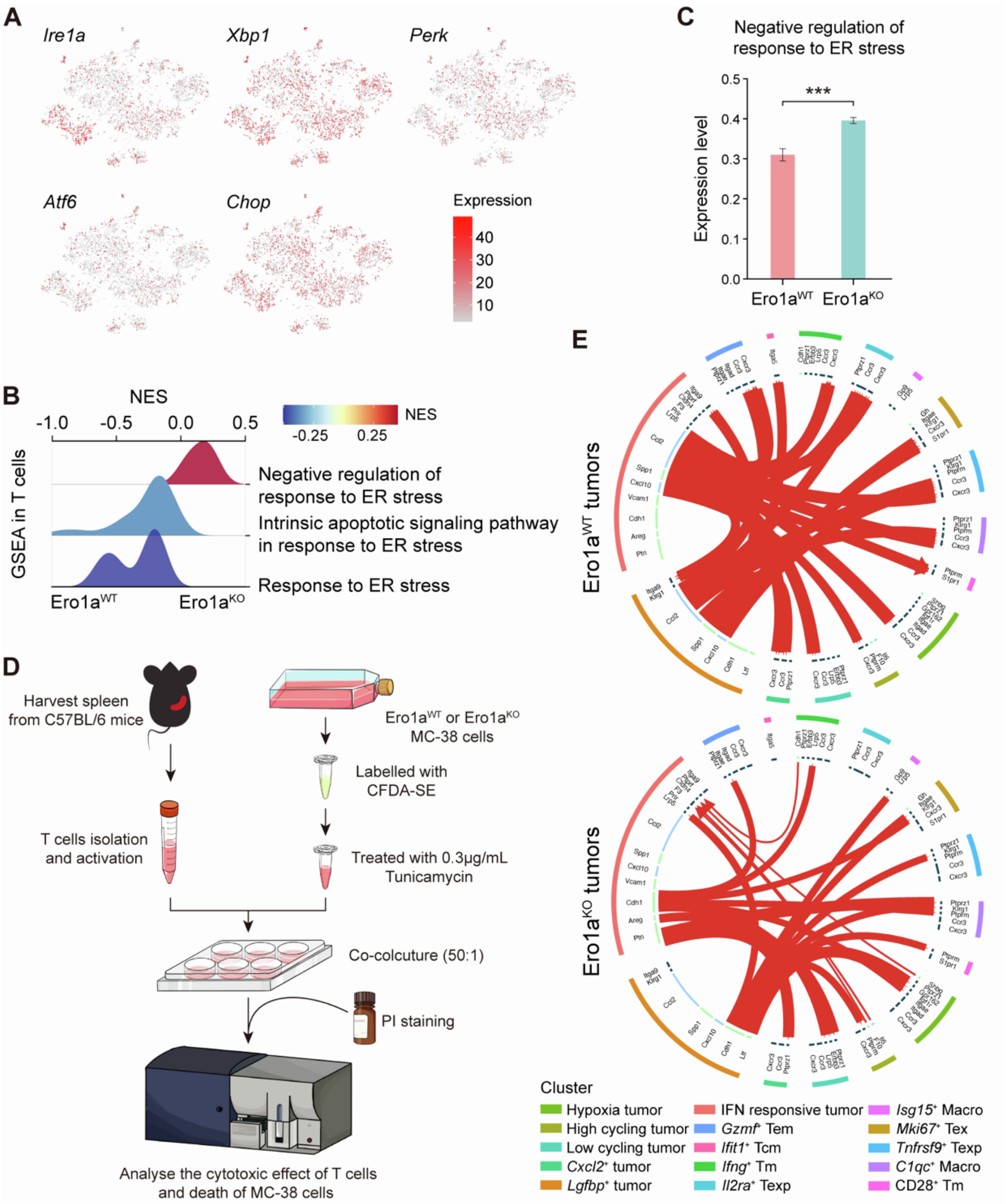

Figure S8: ERO1A induces T cell dysfunction by transmissible ER stress, related to Figure 5.

(A) t-SNE map of unfolded protein response-related genes in T cells, based on the scRNA-seq data in MC-38 therapeutic models. Colored by expression levels.

(B) GSEA of T cell subsets showing lower enrichment of response to ER stress response and intrinsic apoptotic signaling pathway in T cells from Ero1a^{KO} tumors compared with counterpart from WT controls, based on the scRNA-seq data in MC-38 therapeutic models.

(C) Bar plot comparing the signature of negative regulation of response to ER stress in Ero1a^{WT} and Ero1a^{KO} tumors, based on the scRNA-seq data in MC-38 therapeutic models. Data presented as means ± SDs. ***p < 0.001. Two-sided Student's t test.

(D) Schematic diagram of the T cell cytotoxic functional assay.

(E) Cell-to-cell interaction analyses within Ero1a^{WT} or Ero1a^{KO} tumors by iTALK algorithm, based on the scRNA-seq data in MC-38 therapeutic models.

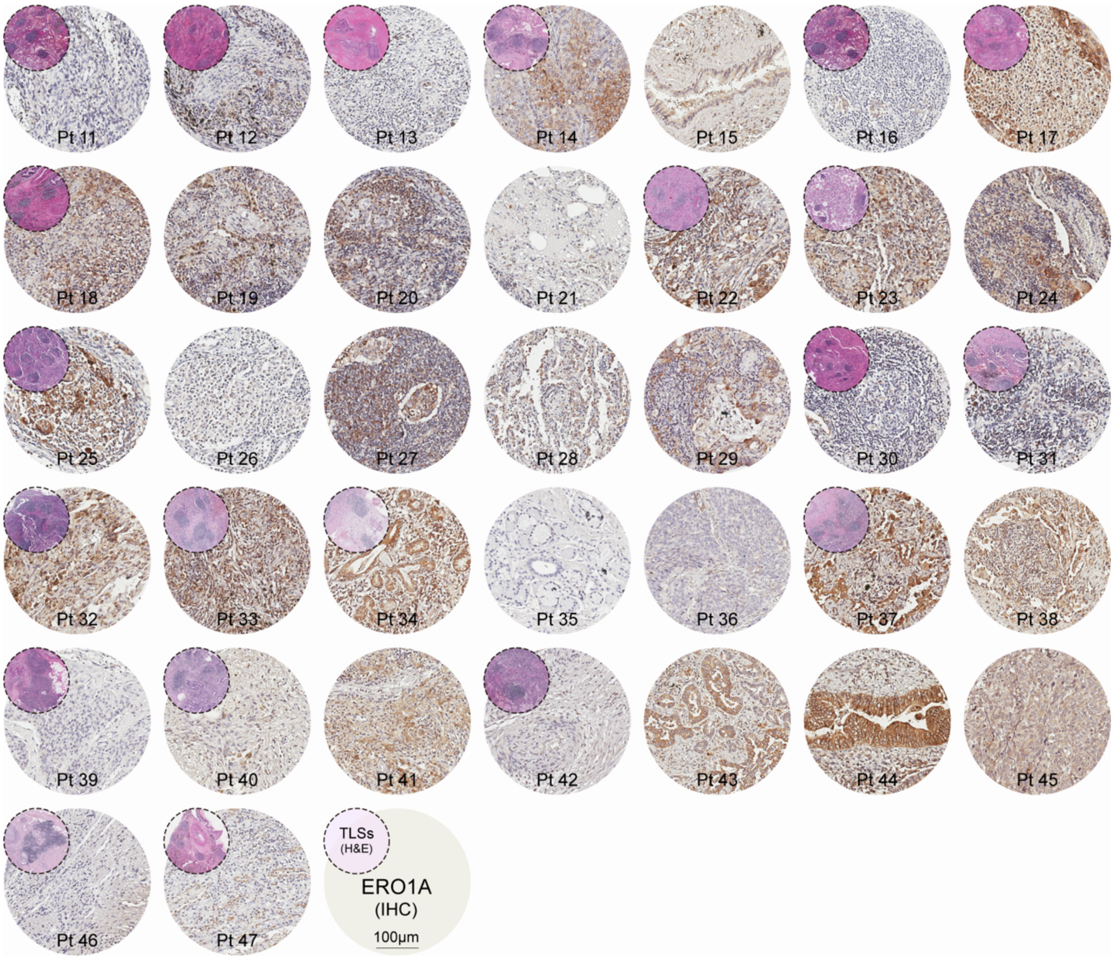

Figure S9: ERO1A expression levels in the included NSCLC patients, related to Figure 6. Representative immunohistochemistry staining of ERO1A in the 37 lung tumors treated with neoadjuvant immunotherapy. IHC staining distinguished 15 patients with low ERO1A expression (Pt 11, Pt 12, Pt 13, Pt 16, Pt 21, Pt 26, Pt 30, Pt 31, Pt 35, Pt 36, Pt 39, Pt 40, Pt 42, Pt 46, and Pt 47) and 22 patients with high ERO1A expression (Pt 14, Pt 15, Pt 17, Pt 18, Pt 19, Pt 20, Pt 22, Pt 23, Pt 24, Pt 25, Pt 27, Pt 28, Pt 29, Pt 32, Pt 33, Pt 34, Pt 37, Pt 38, Pt 41, Pt 43, Pt 44, and Pt 45). Tertiary lymphoid structures are identified by H&E staining and denoted by dotted line. IHC, immunohistochemistry. TLSs, tertiary lymphoid structures. Scale bar = 100μm.

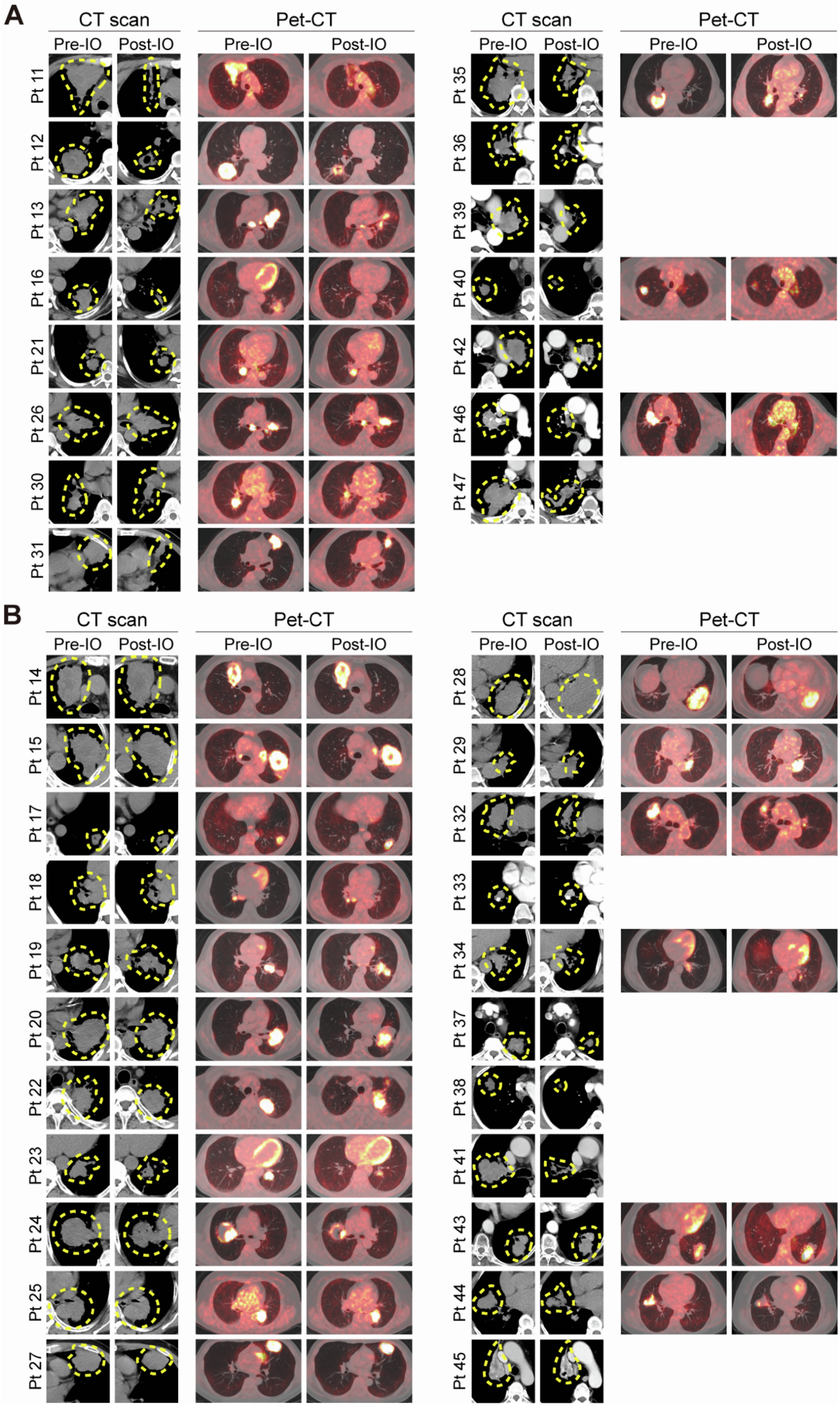
A
CT scan
Pre-IO
Post-IO
Pet-CT
Pre-IO
Post-IO
Pt 11
Pt 12
Pt 13
Pt 16
Pt 21
Pt 26
Pt 30
Pt 31
Pt 35
Pt 36
Pt 39
Pt 40
Pt 42
Pt 46
Pt 47
B
Pt 14
Pt 15
Pt 17
Pt 18
Pt 19
Pt 20
Pt 22
Pt 23
Pt 24
Pt 25
Pt 27
Pt 28
Pt 29
Pt 32
Pt 33
Pt 34
Pt 37
Pt 38
Pt 41
Pt 43
Pt 44
Pt 45

Figure S10: ERO1A attenuates anti-tumor efficacy of aPD-1 blockade in patients with lung cancer, related to Figure 6.

(A) Computed tomography (CT) scan images and available corresponding PET-CT scan images of the 15 patients in ERO1A^{low} group. CT scan was performed before (pre-IO) and after immunotherapy (pos-IO), respectively. IO, immunotherapy. Tumor is denoted by dotted line.

(B) Computed tomography (CT) scan images and available corresponding PET-CT scan images of the 22 patients in ERO1A^{high} group. CT scan was performed before (pre-IO) and after immunotherapy (pos-IO), respectively. IO, immunotherapy. Tumor is denoted by dotted line.

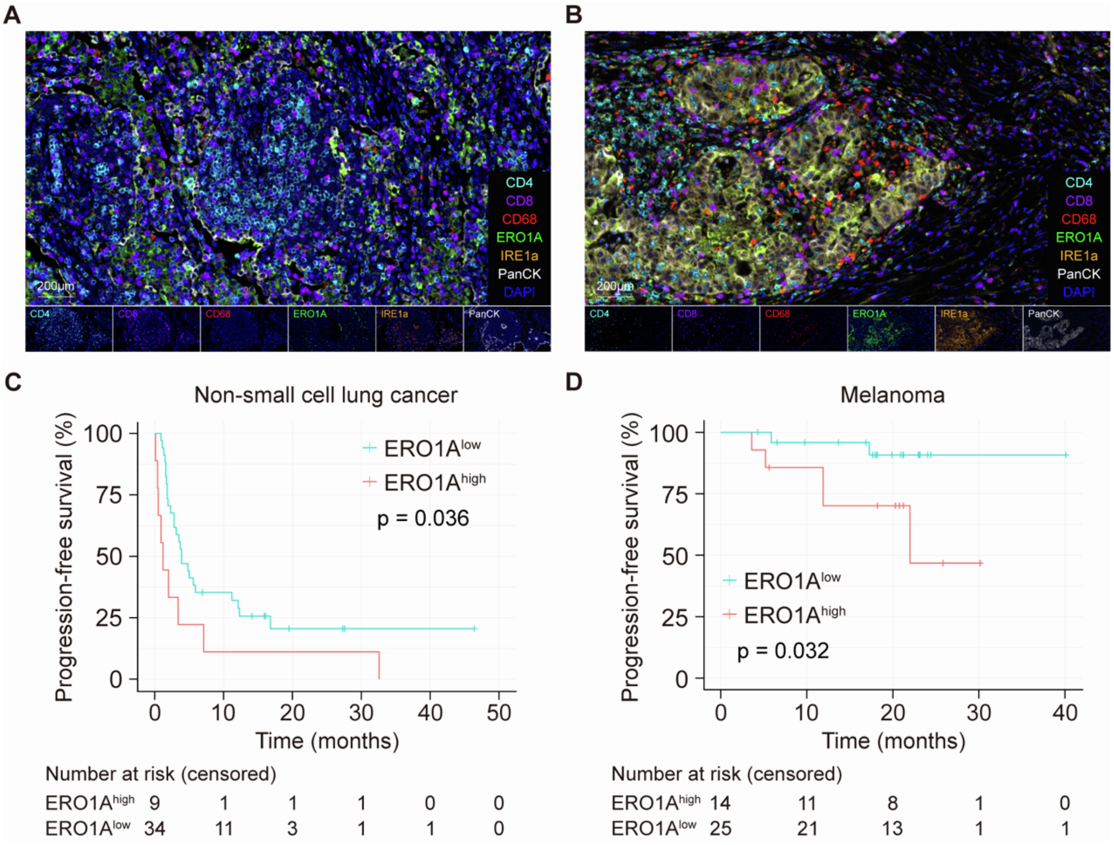

Figure S11: ERO1A as a biomarker in patients treated with immunotherapy, related to Figure 6. (A and B) Multiplex IHC (mIHC) staining of CD4 (cyan), CD8 (violet), CD68 (red), ERO1A (green), IRE1a (orange), PanCK (white), and DAPI (blue) of lung tumors treated with neoadjuvant immunotherapy. Representative mIHC staining of resected tumor samples from patients with ERO1A low (A) or ERO1A high (B) expression (n = 3 patients). Scale bars, 200 µm.

(C) Progression-free survival (PFS) for NSCLC patients stratified by the ERO1A expression based on the GEO: GSE190265 dataset. Kaplan-Meier curves of PFS for ERO1A high (n = 9 patients) and low (n = 34 patients). Log-rank test.

(D) PFS for melanoma patients stratified by the ERO1A expression based on the ENA: PRJEB23709 dataset. Kaplan-Meier curves of PFS for ERO1A high (n = 14 patients) and low (n = 25 patients). Log-rank test.

Table S1. Primers used for RT-qPCR, related to the STAR Methods.

**Gene**	**NCBI ID**	**Size (BP)**	**Sequence**	**Tm (℃)**	**GC (%)**
β-actin	NM_007393.3	287	F: GTGACGTTGACATCCGTAAAGA	58.7	45.5
			R: GTAACAGTCCGCCTAGAAGCAC	61	54.6
Ire1a	NM_023913.2	166	F: GAGAATCAGACGAGCACCCAA	60.07	52.38
			R: GTCTGATGAAGCAGGGTGATGG	61	54.55
Xbp1	NM_001271730.1	178	F: ATTCTGAGTCTGATATCCTTTTGGG	58.4	40
			R: TCCAGCTTGGCTGATGAGGT	61.2	55
Atf4	NM_001287180.1	126	F: AGACACCGGCAAGGAGGATG	61.9	60
			R: AAGAGCTCATCTGGCATGGTTT	60.29	45.45
Atf6	M_001081304.1	133	F: CAAATAGCCAACAGAAAGCCC	57.76	47.62
			R: TAATACACTTGCAGCTCACTCCC	60.37	47.83
Ddit3	NM_001290183.1	133	F: CTGCCTTTCACCTTGGAGACG	61.21	57.14
			R: TTGATTCTTCCTCTTCGTTTCCTG	59	41.67
Perk	NM_001313918.1	85	F: AGTGGGATTTGGACGTGGG	59.62	57.89
			R: TCTTTTGAGGAAGTTTTGTGGGTG	59.84	41.67
